# Predicting the unpredicted … brain response: A systematic review of the feature-related visual mismatch negativity (vMMN) and the experimental parameters that affect it

**DOI:** 10.1371/journal.pone.0314415

**Published:** 2025-02-27

**Authors:** Alie G. Male

**Affiliations:** Department of Psychiatry and Human Behavior, School of Medicine, University of California, Irvine, Irvine, CA, United States of America; University of Hamburg, GERMANY

## Abstract

In this systematic review and meta-analysis, I consider aspects of experimental design that affect the visual mismatch negativity (vMMN)—an electrophysiological (neural) correlate of prediction error in vision that is typically largest between 150 ms and 300 ms in the event-related potential (ERP) at occipito-parietal regions on the scalp. I compiled data from 145 published studies investigating changes in a single property or feature of visual input. This review provides a concise summary of the vMMN literature on unexpected changes in features of visual input, outlining the most used (according to review) and optimal (following discussion on theoretical and practical implications) parameters of experiments investigating feature deviance for posterity as well as contemporary research. The data compiled was analysed to reveal meaningful relationships between aspects of experimental design and vMMN mean amplitude and peak latency. Results suggest that whether a control for adaptation is used, whether attention is towards vs. away from the stimulus of interest, and stimulus presentation time determines mean amplitude. Whether attention is towards vs. away from the stimulus of interest, the time between the stimulus of interest, deviant probability, and the number of standards separating deviants determines peak latency. There is also some indication that magnitude of deviance affects mean amplitude in studies exploring orientation deviance. This review and its findings elucidate potentially fruitful areas of future research.

## 1. Introduction

This review considers the evidence for aspects known (and perhaps unknown) for affecting the visual mismatch negativity (vMMN)—a canonical signature for prediction error in the visual system. To appreciate the factors that may affect the vMMN, this review considers how the vMMN is evoked and how it is measured.

Näätänen, Gaillard, and Mäntysalo [1] discovered an electrophysiological index of extra brain processing for unexpected changes in auditory input—the mismatch negativity (MMN). Näätänen [2] initially conceptualised the MMN as a mismatch signal, evoked by a physical mismatch between the memory trace of a repeated stimulus and a current stimulus—hence the *mismatch* in MMN. This conceptualization has evolved and the MMN is now widely regarded as a neural correlate of deviance detection/prediction error—an integral component in predictive coding theory [3–9].

The MMN is a ubiquitous phenomenon, occurring in sleeping infants [10, 11], comatose patients [12, 13], and in animals, such as cats [14] and mice [15]. The MMN occurs for many kinds of unanticipated change in auditory input, including auditory input that violates a category or rule established by the regularly occurring auditory input. For example, irregular tonal repetitions heard in sequences of rising or falling tones evoke the MMN [16]. Similarly, if a rule dictates one pairing of features (e.g., the higher the frequency, the louder the tone), a tone that does not adhere to the rule (e.g., a higher frequency paired with a quieter tone) will evoke the MMN [17]. These kinds of violations represent abstract irregularities and their capacity to evoke the MMN support the conceptualisation of the MMN as a prediction error rather than a mismatch signal.

The MMN provides valuable insights into how the brain processes and detects changes in auditory stimuli without conscious awareness, revealing unconscious processing, predictive coding, and how the brain adapts to its environment. The MMN is also altered in various neurological and psychiatric conditions, therefore, studying these alterations can provide valuable insights into the underlying neural mechanisms of these disorders and potentially serve as biomarkers to guide treatment [6, 7, 18]. Given the powerful utility of the MMN, researchers inevitably explored and discovered analogues of the MMN in other sensory modalities, including olfaction [19], touch [20], and vision [21]. This review is concerned with the vMMN.

Historically, two paradigms have dominated the field of vMMN/MMN research (for a discussion of evolving paradigms see [22]). The oddball paradigm is the traditional and still most popular paradigm [23]. In the oddball paradigm, there are regularly appearing standard stimuli that are interrupted by infrequent deviant stimuli that differ from the standard. In the roving variant of the oddball paradigm, the standards following the deviant are identical to the deviant and, therefore, the deviant of the prior sequence becomes the first standard of the next sequence.

The second paradigm is the multi-feature paradigm [24]. In the multi-feature paradigm, every second trial (i.e., the trial after every standard) is a deviant because a single property of visual input, as opposed to the whole stimulus, determines whether the stimulus represents a standard or a deviant, and while all other properties are unchanged, one deviant can represent a standard with respect to another property. This allows one to test different deviants within a short time, making it an attractive alternative to the oddball paradigm.

The common means of visualizing vMMN is by subtracting the event-related potential (ERP) for the standard from the ERP to the deviant, producing a deviant-minus-standard difference wave with a negativity between 150 ms and 300 ms after stimulus onset at parieto-occipital regions. This is known as the classic vMMN. However, amplitude differences revealed in this way also contain adaptation-related differences, because deviants are physically different from standards and standards occur more frequently than deviants. Adaptation effects refer to changes in neural responses based on recent sensory input and its relation to current stimuli. A key result of adaptation is repetition suppression, where neural responses decrease for repeated versus new stimuli (reviewed in [25]). The increased negativity owing to deviance detection/prediction error *and* adaptation are known as deviant-related negativity (DRN, (e.g., [26–28]). There are two popular methods for controlling for and separating adaptation-related differences (for a review of the mechanisms which underscore adaptation-related differences see [29]. These are the equiprobable control [30] and the cascadic control [31]. While there has been recent discussion about whether the current control paradigms can effectively isolate the effects which make up the adaptation-related differences [32], these paradigms are currently the most popular methods for distinguishing adaptation-related differences from genuine deviance detection.

In an equiprobable control, all standards are replaced with equally probable random stimuli. The deviant is left unchanged and is renamed control. The cascadic control is similarly constructed; however, where there is no regularity in the equiprobable control, there is an uninterrupted regularity in the cascadic control. In both controls the deviant and control are (i) identical to each other, (ii) different from all other stimuli, and (iii) equally infrequent in their respective sequences. Therefore, the ERP for the control provides a measure of adaptation-related difference so that any remaining difference is because there is an irregularity in the oddball sequence but no such violation in the control sequence (i.e., prediction error). Any activity that remains is thought to reflect deviance detection/prediction error and is revealed by subtracting the ERP for the control from the ERP to the deviant, producing a deviant-minus-control difference wave, revealing the genuine vMMN free from adaptation-related differences. Paavilainen et al. distinguished the “genuine” MMN from the classic MMN contributed to by adaptation [33]. A similar distinction is made in the vMMN field [34].

The vMMN has been reported for a variety of visual irregularities, including, but not limited to, faces of different genders [35, 36], changes in facial expressions [37–46], food among non-food items [47], hand laterality [48, 49], asymmetrical patterns appearing among symmetrical patterns [50], object-based irregularities [51, 52], numeric irregularities (i.e., number of items expected to appear [53]), and even semantic irregularities based on word pairings [54]. These findings illustrate how readily the visual system encodes higher-order regularities.

Relatively simple deviants have also been used to evoke the vMMN. I refer to these as feature deviants because they differ from standards in some physical property or dimension (i.e., feature) of the visual stimulus. Some of the earliest publications of the vMMN were to changes in orientation (e.g., [55]), luminance (e.g., [56]), spatial frequency (e.g., [57]), colour (e.g., [58]), and shape or size (e.g., [26]). Interestingly, some recent studies have failed to find a vMMN for isolated changes in physical properties of visual stimuli, particularly those processed at the earliest stages of the visual system’s processing hierarchy, such as orientation and contrast [34, 59], encouraging one to consider what aspects of experimental design, including the feature itself, predicts vMMN characteristics or presence.

Accordingly, the current work’s objectives are to delineate which aspects of experimental design affect the vMMN or its presence. To that end, I reviewed the vMMN literature in which feature deviants were used to evoke the vMMN. This tabulated summary of the literature summarises the state of this research and allows for exploratory analyses of the aggregated vMMN data, potentially uncovering overarching trends that individual studies, limited by methodological differences or sample sizes, may fail to show.

## 2. Methods

### 2.1. Literature search and study selection

A systematic search was conducted on 3 April 2024. Published studies were identified in Scopus, Web of Science, and Google Scholar using the following Boolean search: (“mismatch negativity” OR vMMN) and (vision OR visual OR “visual feature” OR “deviant feature”) and (spatial frequency OR color OR colour OR orientation OR size OR shape OR phase OR luminance OR location OR "motion direction" OR motion OR contrast OR duration OR omission) by author AM.

The following criteria were necessary for inclusion: (i) original peer-reviewed articles written in English (e.g., the data is original and not published elsewhere such as reviews, books, protocols, and conference abstracts), (ii) data from at least one healthy adult sample is reported, (iii) ERP data is reported and (iv) brain activity was isolated to visual processing in at least one condition.

Studies were excluded if (i) in all conditions the change in the visual stimulus was relatively complex such that higher-order processing would become necessary such as facial or emotional expressions, associative relationships, and lexical stimuli, or (ii) the vMMN or negativity for deviants in the vMMN time-window was not reported/shown. Studies in which authors described the increased negativity to deviants as something other than vMMN were not excluded. For example, Kenemans et al. [36] described the response as rareness-related negativity (see also [37]). Others have described it as change-related negativity (e.g., [38]), N200/N2 difference (e.g., [39–41]), or N270 [42].

After removing duplicates, abstracts were screened for inclusion criteria (original peer-reviewed articles written in English, visual ERP data from at least one healthy adult sample). The remaining publications were examined (whole text) for exclusion criteria (brain activity was not isolated to visual processing in at least one condition and/or the change in the visual stimulus was relatively complex such that higher-order processing would become necessary). Quality and bias assessment of included studies was conducted via the Review Manager (RevMan version 5.3) software for Cochrane reviews.

### 2.2. Data extraction and tabulation

For consistency, outcome measures were mean amplitude (not peak amplitude) and peak latency. These were estimated from figures where possible. I denote where peak latencies were calculated as the midpoint of the time-window of interest because (i) peak latencies were not reported, and (ii) there are no difference waves from which to derive an accurate peak latency estimate. Where authors report no significant negativity for an experiment or condition but there are no values or figures from which to obtain a value, a mean amplitude of “0” appears italicized in red and boldface. Otherwise, where there was a significant negativity but mean amplitude was not reported and could not be estimated from figures, I leave a blank. Where there is no significant vMMN, peak latency is blank.

I give details of separate conditions of a single experiment where multiple feature deviants were tested (one row for each feature). I give details of all conditions that did not produce a vMMN. Where multiple conditions of an experiment produced a vMMN, I give details of the condition that produced the largest negativity. When a significant deviant-minus-standard difference exists, yet there is no deviant-minus-control difference, I give details of both deviant-minus-standard and deviant-minus-control mean amplitudes (e.g., [60]). When both are significant, I give the deviant-minus-control mean amplitude only. Mean amplitudes appear in red where negativity was not significant (i.e., no vMMN reported for that condition).

In addition to mean amplitude and peak latency, the following were extracted for each entry. I leave a blank where a piece of information was absent.

- Authors.
- Publication year.
- Deviant feature.
- The number of participants in the corresponding data for a given condition or experiment (N).
- The stimulus of interest (SOI) and whether it appeared centrally or peripherally.
- SOI height (H) and width (W) in degrees of visual angle (°VA).
- Background colour (BG).
- Whether the participant’s task was visual, auditory, or manual.
- The stimulus that occupied the participant’s attention.
- The difference between the standard and the deviant (D–S) in the units measured, for example, degrees (°), candles per meter squared (cd/m^2^), or cycles per degree (cpd).
- SOI duration and their inter-stimulus-interval (ISI) in milliseconds (ms) rounded to the nearest whole integer. If SOI duration or ISI was variable or jittered, the shortest SOI duration and longest ISI appears italicized in boldface.
- Deviant probability (D %) within the oddball sequence (as a proportion of 1) if stated.
- The minimum number of standards (Min. S) between deviants if stated.
- Whether the authors compared physically identical stimuli.
- Whether there was a control for adaptation.
- The reference for processing the EEG data.
- Method of ocular artifact rejection or correction.
- Low-pass (LP) and high-pass (HP) filters used to process the data (in Hz).
- Electrode or region of interest (ROI) from which mean and/or latency was reported.
- The vMMN time-window used to extract mean amplitudes (in ms).
- Mean amplitude of the genuine vMMN or classic vMMN where there was no control (to two decimal places in μV).
- The time of vMMN peak latency (nearest whole integer in ms).

### 2.3. Meta-analytic procedure

Statistical analyses were performed in SPSS. One-way ANOVAs were used to explore the effect of *deviant feature*. I performed forward linear regression analyses on vMMN amplitude and peak latency separately. Including deviant feature a total of 10 predictors were:

1. SOI duration (in ms rounded to the nearest whole integer).
2. ISI duration (in ms rounded to the nearest whole integer).
3. Deviant probability (as a proportion of 1).
4. The minimum number of standards between deviants (0 is none stated).
5. Whether a minimum number of standards was specified or not.
6. Whether attention was towards vs. away from the SOI.
7. Whether there was a fixation task or not.
8. Whether there was a physical control or not.
9. Whether there was a control for adaptation or not.
10. Deviant feature.

Because magnitude of deviance standard vs. deviant differences for most properties of visual input were not easily standardized, the aforementioned analysis did not include magnitude of deviance as a predictor. Instead, magnitude of deviance effect was examined in orientation and spatial frequency entries separately, post-standardization. For orientation entries, magnitude of deviance for orientation was standardized as the difference between the deviant and the standard orientation, divided by 90 if ≤ 90° or minus 90 and then divided by 90 if > 90° (e.g., if the deviant had an orientation of 90° and the standard had an orientation of 180°, the difference is 1 or if the deviant had an orientation of 155° and the standard had an orientation of 45°, the difference is 0.22). The calculation ensures orientation changes that are >90° are not represented as larger magnitudes of deviance than same orientation changes that are <90°.

For spatial frequency entries, magnitude of deviance for spatial frequency was standardized as the (absolute) difference between deviant and standard and divided by the larger spatial frequency (e.g., if the deviant had a spatial frequency of 1.2 cpd and the standard had a spatial frequency of 0.6 cpd, the difference is 0.5). Initially, analyses included predictors identified as influencing the vMMN amplitude or latency in their respective analysis. Thereafter, analyses included all 10 predictors (excluding deviant feature and including magnitude of deviance).

## 3. Results

### 3.1. Included studies

After removing duplicates, 948 records were screened for inclusion criteria (original peer-reviewed articles written in English). The remaining 850 records were examined for further inclusion criteria. Of these, 250 had visual ERP data. Of these, 243 also had at least one healthy adult sample.

A further 98, were excluded because there was no vMMN data and/or the change in the visual stimulus was relatively complex such that higher-order processing would become necessary. Systematic review data and flow chart in supplementary materials (**S1 Data**).

Ultimately, 145 published peer-reviewed articles reported at least one condition in which they manipulated a relatively non-complex element of visual stimuli, such as orientation, contrast, luminance, spatial frequency, colour, shape/size, location, motion direction, duration/omission, or phase. The summary of risk of bias suggests a low proportion of vMMN studies indicate effect sizes (details in supplementary materials S2 and S3 Texts). To increase review sensitivity, studies were not excluded based on presence/absence of effect size or any of the captured variables including mean and peak amplitudes.

**Table 1** typifies the existing approach to vMMN research while also illustrating the kinds of feature deviants that have been used to evoke the vMMN. Entries are chronologically ordered by deviant feature.

**Table 1:** Systematic review of studies in which the deviant is a single feature of the visual stimulus.

Study	N	SOI	W (°VA)	H (°VA)	BG Colour	Task Modality	Attention On	D-S	SOI (ms)	ISI (ms)	D%	Min. S	PC	AC	Ref	Ocular- artifact correction	HP (Hz)	LP (Hz)	Epoch	ROI	mean vMMN (μV)	peak vMMN (ms)	Time-window
**Orientation**																							
Czigler_1990 [61]	9	Chevrons within rectangular frame	1.27	0.77		Visual	Chevron deviants	180°	83	417	0.20	2	none	none	Linked- earlobes	rejection; EOG threshold exclusion	0.20	50	0–768	Oz	-3.76a	237a	210–270
Fu_2003 [62]	12	Central square-wave gratings	3.20	3.20	Black	Visual	Spatial Frequency	90°	100	***100***	0.10	1	Reverse roles	none	Right mastoid	rejection; discard epochs with eye blinks	0.10	40	-200–800	Occipital	-1.90a	192	180–220
Astikainen_2004 [63]	8	Central light bar		3.40	Black	Auditory	Words	90°	50	***450***	0.10	2	Deviant alone	none	Linked-mastoids	rejection; EOG/EEG threshold exclusion	0.10	30	-50–500	Pz	-1.28a	180b	160–200
Henderson_2006 [64]	12	Peripheral Gabor patches			Grey	Visual	Fixation square	90°	100	900	0.20		Reverse Roles	none	Right mastoid	rejection; EOG threshold exclusion	0.08	100	-100–800	P10	‐1.00a		180–320
Astikainen_2008 [Short SOA] [65]	10	Central dark bar		3.90	Grey	Auditory	Words	36°	100	***400***	0.10	2	Reverse roles	Equi-probable	Average	rejection; EOG/EEG threshold exclusion	0.10	30	-100–500	Occipital	-0.69a	195b	185–205
Astikainen_2008 [Long SOA] [65]	10	Central dark bar		3.90	Grey	Auditory	Words	36°	100	***1*,*100***	0.10	2	Reverse roles	Equi-probable	Average	rejection; EOG/EEG threshold exclusion	0.10	30	-100–500	Occipital	0.27a		185–205
Kimura_2009 [66]	12	Central grey bar	0.50	3.00	Grey	Visual	Bar edges	36°	100	***500***	0.20	2	Reverse roles	Equi-probable	Nose-tip	rejection; EOG/EEG threshold exclusion	0.10	30	-100–400	T6(P8)	-1.60a	225b	200–250
Czigler_2009 [67]	14	Peripheral grid pattern	13.10	9.30	Grey	Visual	Target quadrangle width	90°	68	***702***	0.07	10	none	none	Linked-mastoids	rejection; EOG/EEG threshold exclusion	0.10	30	-100–500	Right posterior	-1.01a	280b	270–290
Czigler_2010 [68]	24	Peripheral line segments	0.04	0.37	Grey	Visual	Central line segment	30°	70	***290***	0.15		Reverse roles	none	Average mastoids	rejection; EOG/EEG threshold exclusion	0.15	30	-100–500	Oz	-0.60	166	162–170
Sulykos_2011 [69]	12	Peripheral Gabor patches	0.80	0.80	Black	Visual	Spaceship	90°	80	480	0.18		Reverse roles	none	Linked-mastoids	rejection; EOG/EEG threshold exclusion	0.10	30	-100–400	Oz	-2.55	130	121–131
Tomio_2012 [Deviant 1] [70]	13	Ramachandran visual pattern	4.60	3.90	Grey	Auditory	Nursery stories	90°	100	1,000			none	none	Linked-earlobes	rejection; EOG/EEG threshold exclusion	0.50	60	-16.8–700	Pz	0.00		
Tomio_2012 [Deviant 2] [70]	13	Ramachandran visual pattern	4.60	3.90	Grey	Auditory	Nursery stories	180°	100	1,000			none	none	Linked- earlobes	rejection; EOG/EEG threshold exclusion	0.50	60	-16.8–700	Pz		275	
Kimura_2013 [28]	22	Peripheral grey bars	0.40	3.00	Grey	Visual	Fixation dot	33°	250	250	0.09	1	Reverse roles	Equi- probable	Nose-tip	rejection; EOG/EEG threshold exclusion	1.00	30	-100–500	PO8	-0.93a	226b	221–231
Sulykos_2013 [71]	12	Peripheral Gabor patches	1.00	1.00	Dark	Visual	Spaceship	30°	80	610	0.07	12	Equi- probable	Equi- probable	Average	rejection; EOG/EEG threshold exclusion	1.00		-100–400	Parieto- occipital	‐0.05a		140–350
Takács_2013 [Experiment 1] [72]	17	Peripheral Gabor patches	1.60	1.60	Grey	Visual	Fixation dot	50°	100	***500***	0.17	3	Reverse roles	none	Average	rejection; EOG/EEG threshold exclusion	0.10	30	-100–400	Parieto- occipital	-0.65	134	120–140
Takács_2013 [Experiment 2] [72]	19	Peripheral Gabor patches	1.60	1.60	Grey	Visual	Fixation dot	90°	100	***500***	0.17	3	Reverse roles	none	Average	rejection; EOG/EEG threshold exclusion	0.10	30	-100–400	Parieto- occipital	-0.84	148	116–176
Shi_2013 [73]	12	Peripheral red rectangles	0.10	0.30	White	Visual	Fixation cross	90°	50	550	0.10	1	none	none	Nose-tip	correction; corrected via EOG regression	0.10	100	-100–400	Occipito- temporal	-1.60	200b	150–250
Wu_2013 [74]	12	Peripheral rectangles	3.44	1.15		Visual	Central number task	90°	50	550	0.10	1	none	none	Nose-tip	correction; EOG artifact correction	0.10	100	-100–400			192	150–200
Kimura_2014 [Experiment 1] [75]	23	Central grey bar	1.00	6.00	Back	Manual	Visuo-motor task	22°	250	***603***	0.18		Reverse roles	none	Nose-tip	rejection; EOG/EEG threshold exclusion	1.50	30	-100–500	Right occipito- temporal	-1.19	225b	200–250
Kimura_2014 [Experiment 2] [75]	21	Central grey bar	1.00	6.00	Black	Manual	Visuo-motor task	22°	250	***610***	0.18		Reverse roles	none	Nose-tip	rejection; EOG/EEG threshold exclusion	1.50	30	-100–500	Right occipito- temporal	-0.92	265b	240–290
Noyce_2014 [76]	20	Peripheral Chevrons	1.40	1.40	Grey	Visual	Central Chevron	180°	50	1,000	0.10		none	none	Average	rejection; visual inspection	0.25	60	-236–464	Posterior	-0.36a	214b	144–284
Wang_2014a [77]	14	Peripheral facial models	3.68	3.42	White	Visual	Fixation cross	90˚	100	500	0.13	2	Equi- probable	Equi- probable	Nose-tip	correction; corrected via EOG regression	0.05	30	-100–600	P9/10	-1.90	225b	150–300
Wang_2014b [78]	16	Peripheral faces	3.42	3.68	Grey	Visual	Fixation cross	90°	100	500	0.20	2	Reverse Roles	none	Nose-tip	correction; corrected via EOG regression	0.05	30	-100–500	Occipito- temporal	-2.50	175a	140–200
Qian_2014 [79]	14	Peripheral red rectangles	0.10	0.30	White	Visual	Fixation cross	90°	50	550	0.10	1	none	none	Nose-tip	rejection; EOG/EEG threshold exclusion	0.10	100	-100–500	Occipital- temporal	1.07	200a	150–250
Farkas_2015 [80]	27	Peripheral Gabor patches	7.70	7.70	Grey	Visual	Fixation cross	90°	200	***480***	0.20		Reverse roles	none	Average	correction; ICA	0.10	30	-100–600	Sagittal parieto- occipital	-0.30	145b	90–200
Jack_2015 [81]	12	Annular sine-wave grating	3.20	3.20	Grey	Visual	Grating orientation	45°	500	16	0.20	3	Equi- probable	Equi- probable	Linked-earlobes	rejection; EOG/EEG threshold exclusion	0.10	35	-100–500	Right Posterior	-1.71a	250	240–290
Kimura_2015 [82]	22	Peripheral grey bars	0.40	3.00	Black	Visual	Fixation dot	33°	250	250	0.09	2	Reverse roles	Equiprobable	Nose-tip	rejection; EOG/EEG threshold exclusion	0.10	30	-100–500	PO8	-1.16	202b	197–207
Sulykos_2015 [83]	14	LoVF matrix of "N"	16.10	3.90	Black	Visual	Spaceship	90°	300	***617***	0.17	3	Reverse roles	none	Nose-tip	rejection; EOG/EEG threshold exclusion	0.10	30	-100–500	Parieto- occipital	-1.03	140	130–150
Sulykos_2015 [83]	14	LoVF matrix of oblique bars	16.10	3.90	Black	Visual	Spaceship	90°	300	***617***	0.17	3	Reverse roles	none	Nose-tip	rejection; EOG/EEG threshold exclusion	0.10	30	-100–500	Parieto- occipital	-1.23	127	117–137
Meng_2015 [Supra-threshold condition] [84]	15	Central Tumbling E ototypes			Black	Auditory	/nə/ sound	90˚	300	500	0.13	2	Equi- probable	Equi- probable	Nose-tip	correction; EOG artifact correction	0.10	100	-200–800	Occipito- temporal	-2.58	332	270–430
Meng_2015 [Sub-threshold condition] [84]	15	Central Tumbling E ototypes			Black	Auditory	/nə/ sound	90˚	300	500	0.13	2	Equi- probable	Equi- probable	Nose-tip	correction; EOG artifact correction	0.10	100	-200–800	Occipito- temporal	‐0.06		270–430
Li_2017 [85]	12	Peripheral polygons	0.57	1.70	Grey	Manual	Visuo-motor task	90°	50	600	0.17	1	Equi- probable	Equi- probable		correction; removed influence of EOG on other leads			-100–400	Right occipito- temporal	-0.66a	195a	175–225
Durant_2017 [Deviant 1 D-C] [60]	15	Peripheral Gabor patches	2.45	2.45	Grey	Visual	Fixation dot	74°	120	***600***	0.17		Equi- probable	Equi- probable	Nose-tip	rejection; EOG/EEG threshold exclusion	0.10	30	-100–400	Right parieto- occipital	0.00		150–250
Durant_2017 [Deviant 1 D-S] [60]	15	Peripheral Gabor patches	2.45	2.45	Grey	Visual	Fixation dot	74°	120	***600***	0.17		none	none	Nose-tip	rejection; EOG/EEG threshold exclusion	0.10	30	-100–400	OZ	0.24a		150–250
Durant_2017 [Deviant 2 D-C] [60]	15	Peripheral Gabor patches	2.45	2.45	Grey	Visual	Fixation dot	86°	120	***600***	0.17		Equi- probable	Equi- probable	Nose-tip	rejection; EOG/EEG threshold exclusion	0.10	30	-100–400	Right parieto- occipital	0.00		150–250
Durant_2017 [Deviant 2 D-S] [60]	15	Peripheral Gabor patches	2.45	2.45	Grey	Visual	Fixation dot	86°	120	***600***	0.17		none	none	Nose-tip	rejection; EOG/EEG threshold exclusion	0.10	30	-100–400	Right parieto- occipital	-0.61	196a	150–250
File_2017 [Experiment 1—cascadic] [86]	15	Peripheral line texture	0.09	1.26	Black	Visual	Spaceship	36°	100	500	0.12	4	Reverse roles	Cascadic	Nose-tip	rejection; EOG/EEG threshold exclusion	0.10	30	-100–400	Parieto- occipital	‐0.09		105–190
File_2017 [Experiment 1—equiprobable] [86]	15	Peripheral line texture	0.09	1.26	Black	Visual	Spaceship	36°	100	500	0.12	4	Reverse roles	Equi- probable	Nose-tip	rejection; EOG/EEG threshold exclusion	0.10	30	-100–400	Parieto- occipital	0.03		105–190
Bodnár_2017 [87]	17	Peripheral line texture	0.09	1.26	Black	Visual	Spaceship	90°	100	400	0.12		Reverse roles and Standard only	none	Nose-tip	rejection; EOG/EEG threshold exclusion	0.10	30	-100–400	Parieto- occipital	-1.44	124b	112–132
Yan_2017 [88]	15	Peripheral black arrows	3.42	3.68	White	Visual	Fixation cross	90°	100	500	0.20	2	none	none	Nose-tip	correction; ICA	0.10	30	-300–600	Parieto- occipital	-2.60a	200b	100–300
Urakawa_2017 [89]	10	Peripheral line segments	0.10	1.90	Black	Visual	Central Necker cube	90˚	250	250			none	none	Linked- earlobes	rejection; EOG/EEG threshold exclusion	0.50	50	-200–600	Oz	-1.68a	300	280–406
Flynn_2017 [Deviant 1] [90]	9	Central black-white checkerboard	31.60	28.60	Complex coloured stimuli		Fixation dot	45˚	14	***500***	0.20		Deviant block	none	Right mastoid	rejection; online artifact rejection of eye blinks	1.00	30	-100–500	Occipital	‐0.95		155–195
Flynn_2017 [Deviant 2] [90]	9	Central black-white checkerboard	31.60	28.60	Complex coloured stimuli		Fixation dot	45˚	7	***500***	0.20		Deviant block	none	Right mastoid	rejection; online artifact rejection of eye blinks	1.00	30	-100–500	Occipital	‐0.06		155–195
Sulykos_2017 [Deviant 1] [91]	30	Peripheral diamonds with their diagonals	1.65	1.65	Grey	Visual	Central disc	90°	***480***	***560***	0.20		Reverse roles	none	Nose-tip	rejection; EOG/EEG threshold exclusion	0.10	30	-100–400	Occipital	-1.14	160a	120–219
Sulykos_2017 [Deviant 2] [91]	30	Peripheral diamonds with their diagonals	1.65	1.65	Grey	Visual	Central disc	90°	***480***	***560***	0.20		Reverse roles	none	Nose-tip	rejection; EOG/EEG threshold exclusion	0.10	30	-100–400	Occipital	0.00		120–219
Zhang_2018 [92]	24	Central black arrow	3.68	3.42	White	Visual	Fixation cross	90°	100	500	0.20	2	none	none	Nose-tip	correction; corrected via EOG regression	0.05	30	-100–600	Parieto- occipital	-2.44a	179	100–300
Kimura_2018 [93]	14	Central grey bar	0.90	5.70	Black	Visual	Central grey bar	45˚	250	600	0.19	3	none	none	Nose-tip	correction; ICA	1.00	30	-100–700	P8	-2.30	225b	200–250
Urakawa_2018 [94]	18	Peripheral line segments	1.90	0.10	Black	Visual	Fixation cross	90°	250	250			Equi- probable	Equi- probable	Average	none	0.50	30	-100–500	Oz	-0.19a	188	134–252
Kimura_2019 [95]	35	Central grey bar	1.00	6.00	Black	Manual	Visuo-motor task	90°	250	50	0.20		none	none	Nose-tip	correction; ICA	1.00	30	-100–500	Right occipito- temporal	-1.03	283a	270–350
Smout_2019 [No attention condition] [96]	24	Central Gabor patches	11.00	11.00	Grey	Visual	Fixation dot	20°	100	500	0.14	4	Equi- probable	Equi- probable	Average	correction; ICA	0.25	50	-100–500	PO8	0.17a		150–350
Smout_2019 [Attention condition] [96]	24	Central Gabor patches	11.00	11.00	Grey	Visual	Gabor patch spatial frequency	20°	100	500	0.14	4	Equi- probable	Equi- probable	Average	correction; ICA	0.25	50	-100–500	PO8	-0.63a	206a	150–350
File_2019 [Attend far condition] [97]	15	LoVF Peripheral hourglass shapes	2.31	2.31	Grey	Visual	Bars rotating task with bar above	90°	500	500	0.20		Reverse Roles	Equi- probable	Average	rejection; EOG/EEG threshold exclusion + Eye-tracking^c^	0.10	30	-100–500	PO4	‐0.33a		140–210
File_2019 [Attend close condition] [97]	15	LoVF Peripheral hourglass shapes	2.31	2.31	Grey	Visual	Bars rotating task with bar below	90°	500	500	0.20		Reverse Roles	Equi- probable	Average	rejection; EOG/EEG threshold exclusion + Eye-tracking^c^	0.10	30	-100–500	PO4	-1.76	187a	140–210
Pesonen_2019 [98]	16	Central dark bar		3.90	Grey	Auditory	Words	36°	100	1,100	0.10	2	none	none	Cz	rejection; only noise-free trials averaged	0.10	400	-100–400	Occipital	-1.02a	210	207–248
Czigler_2019 [Deviant 1 offset] [99]	18	Peripheral diamonds with their diagonals	0.33	9.90	Grey	Visual	Central disc	90°	***480***	***560***	0.20	1	Reverse Roles	none	Nose-tip	rejection; EOG/EEG threshold exclusion	0.10	30	-100–500	Occipital	-1.02	160	120–202
Czigler_2019 [Deviant 2 offset] [99]	18	Peripheral white lines	0.33	9.90	Grey	Visual	Central disc	90°	***480***	***560***	0.20	1	Reverse Roles	none	Nose-tip	rejection; EOG/EEG threshold exclusion	0.10	30	-100–500	Occipital	-0.76	162	120–202
Czigler_2019 [Deviant 1 onset] [99]	18	Peripheral diamonds with their diagonals	0.33	9.90	Grey	Visual	Central disc	90°	***480***	***560***	0.20	1	Reverse Roles	none	Nose-tip	rejection; EOG/EEG threshold exclusion	0.10	30	-100–500	Occipital	0.17		120–202
File_2020 [100]	13	LoVF Peripheral Diamonds	0.14	4.21	Grey	Visual	Bars rotating task and bar above	90°	500	500	0.20		Reverse Roles	none	Average	rejection; EOG/EEG threshold exclusion + Eye-tracking^c^	0.10	30	-100–500	PO4	-1.92	181	152–200
Male_2020 [Experiment 1: Gabor-fixation condition] [34]	24	Central Gabor patches	3.00	3.00	Grey	Visual	Fixation dot	36°	100	400	0.20	2	Equi- probable	Equi- probable	Nose-tip	correction; ICA + Eye-tracking	0.10	40	-100–400	P8	0.18		200–250
Male_2020 [Experiment 2] [34]	24	Central Gabor patches	3.00	3.00	Grey	Visual	Fixation dot	36°	100	400	0.13	2	Cascadic	Cascadic	Nose-tip	correction; ICA + Eye-tracking	0.10	40	-100–400	P8	0.25		200–250
Male_2020 [Experiment 1: bar-fixation condition] [34]	24	Central grey bar	0.50	3.00	Black	Visual	Fixation dot	36°	100	400	0.20	2	Equi- probable	Equi- probable	Nose-tip	correction; ICA + Eye-tracking	0.10	40	-100–400	P8	0.62		200–250
Male_2020 [Experiment 1: bar-edge condition] [34]	24	Central grey bar	0.50	3.00	Black	Visual	Bar edges	36°	100	400	0.20	2	Equi- probable	Equi- probable	Nose-tip	correction; ICA + Eye-tracking	0.10	40	-100–400	P8	0.79		200–250
Male_2020 [Experiment 1: Gabor-edge condition] [34]	24	Central Gabor patches	3.00	3.00	Grey	Visual	Gabor patch edges	36°	100	400	0.20	1	Equi- probable	Equi- probable	Nose-tip	correction; ICA + Eye-tracking	0.10	40	-100–400	P8	‐0.13		200–250
Petro_2020 [Experiment 1: task direction = orientation diff] [101]	24	Peripheral line segments	0.77	0.11	Grey	Manual	Visuo-motor task	144˚	100	***491***	0.13		Equi- probable	Equi- probable	Nose-tip	rejection; EOG/EEG threshold exclusion	0.10	30	-100–450	Occipital	0.05		200–350
Petro_2020 [Experiment 1: task direction ~ orientation diff] [101]	24	Peripheral line segments	0.77	0.11	Grey	Manual	Visuo-motor task	144˚	100	***491***	0.13		Equi- probable	Equi- probable	Nose-tip	rejection; EOG/EEG threshold exclusion	0.10	30	-100–450	Occipital	0.15		200–350
Petro_2020 [Experiment 2: task direction = orientation diff] [101]	26	Peripheral line segments	0.77	0.11	Grey	Manual	Visuo-motor task	144˚	100	***491***	0.13		Equi- probable	Equi- probable	Nose-tip	rejection; EOG/EEG threshold exclusion	0.10	30	-100–450	Occipital	0.01		200–350
Petro_2020 [Experiment 2: task direction ~ orientation diff] [101]	26	Peripheral line segments	0.77	0.11	Grey	Manual	Visuo-motor task	144˚	100	***491***	0.13		Equi- probable	Equi- probable	Nose-tip	rejection; EOG/EEG threshold exclusion	0.10	30	-100–450	Occipital	‐0.14		200–350
Kurita_2021 [102]	16	Central blue/red grating	4.30	4.30	Black	Visual	Grating color	90˚	200	100			none	none	Linked- earlobes	rejection; EOG/EEG threshold exclusion	0.50	300	-100-500	Oz	-1.86a	237	100–250
Tse_2021 [103]	22	Peripheral grey bars	0.40	3.00	Black	Visual	Fixation cross	33°	250	368	0.09	11	Equi- probable	Equi- probable	Nose-tip	correction; corrected via EOG regression	0.10	30	-100–500	O2	-0.45a	226a	150–250
Schlossmacher_2022 [104]	25	Peripheral white lines in matrix	10.00	10.00	Black	Visual	Shape change	90°	100	700	0.20	1	none	none	Average	rejection; visual inspection	0.10	25	-200–600	Posterior	-0.45a	262	150–350
Csikós_2023 [105]	21	LoVF Diamonds with their diagonals	1.57	1.57	Grey	Visual	Fixation cross	90°	400	400	0.15		Reverse roles	none	Nose-tip	correction; ICA	0.10	30	-100–400	Right parieto- occipital	-1.05	135b	120–150
Male_2023 [Deviant 1] [106]	21	Central Gabor patches	22.00	22.00	Grey	Visual	Letters	15°	80	280	0.18	2	Equi- probable	Equi- probable	Average	correction; ICA	0.10	40	-50–350	Middle parieto- occipital	0.16		197–207
Male_2023 [Deviant 2] [106]	21	Central Gabor patches	22.00	22.00	Grey	Visual	Letters	30°	80	280	0.18	2	Equi- probable	Equi- probable	Average	correction; ICA	0.10	40	-50–350	Middle parieto- occipital	0.25		197–207
Male_2023 [Deviant 3] [106]	21	Central Gabor patches	22.00	22.00	Grey	Visual	Letters	60°	80	280	0.18	2	Equi- probable	Equi- probable	Average	correction; ICA	0.10	40	-50–350	Middle parieto- occipital	0.22		197–207
Male_2024 [CVF condition] [59]	18	Central Gabor patches	4.00	4.00	Grey	Visual	Fixation cross	33°	100	400	0.17	3	Reverse roles	Equi- probable	Average	correction; ICA	0.01	40	-100–400	Right parieto- occipital	‐0.18a		197–207
Male_2024 [LoVF condition] [59]	16	LoVF Gabor patches	4.00	4.00	Grey	Visual	Fixation cross	33°	100	400	0.17	3	Reverse roles	Equi- probable	Average	correction; ICA	0.01	40	-100–400	Right parieto- occipital	0.00		197–207
**Contrast**																							
Nyman_1990 [107]	9	Peripheral square-wave gratings	2.00	2.00		Visual	Fixation dot	-0.48 M	100	490	0.10		Reverse roles	none	Right mastoid	rejection; EOG/EEG threshold exclusion	0.10	100	0–300	Oz	‐0.28		100–200
Luo_1997 [108]	12	Coloured scenery	4.30	2.80	Grey	Visual	Contrast increment	Contrast increment	28	***672***	0.18		none	none	Nose-tip	none	0.10	40	-50–550	Oz	-1.75	168	90–220
Wei_2002 [109]	12	Coloured scenery	2.46	1.60	Stimulus	Visual	Contrast increment	Contrast increment		***700***	0.15		none	none	Nose-tip	correction; EOG automatically corrected by NeuroScan	0.10	40	-52–600	Oz	-1.65a	152a	150–200
Male_2020 [Experiment 2] [34]	24	Central Gabor patches	3.00	3.00	Grey	Visual	Fixation dot	+0.33 M	100	400	0.13	1	Cascadic	Cascadic	Nose-tip	correction; ICA + Eye-tracking	0.10	40	-100–400	P8	1.00		200–250
Male_2024 [CVF condition] [59]	18	Central Gabor patches	4.00	4.00	Grey	Visual	Fixation cross	-0.453 M	100	400	0.17	3		Equi- probable	Average	correction; ICA	0.01	40	-100–400	Right parieto- occipital	0.07a		150–200
Male_2024 [oLVF condition] [59]	16	LoVF Gabor patches	4.00	4.00	Grey	Visual	Fixation cross	-0.453 M	100	400	0.17	3		Equi- probable	Average	correction; ICA	0.01	40	-100–400	Right parieto- occipital	0.00		150–200
**Luminance**																							
Stagg_2004 [56]	12	Peripheral white vertical bars	0.34	2.23	Black	Visual	Fixation square	2.76 cd/m2	200	***642***	0.06		Reverse roles	none	Fz	rejection; EOG/EEG threshold exclusion	0.05	100	0–512	Occipital	-1.67a	305b	210–400
Kimura_2010a [110]	12	Peripheral discs	1.40	1.40	Black	Visual	Fixation letter	127.36 cd/m2	150	450	0.20	4	Reverse roles	none	Nose-tip	rejection; EOG/EEG threshold exclusion		35	-100–700	PO7	-5.25	240b	220–260
Kimura_2010b [111]	12	Peripheral discs	2.50	2.50	Black	Visual	Fixation cross	76 cd/m2	80	***720***	0.20	1	Reverse roles	none	Nose-tip	rejection; EOG/EEG threshold exclusion	1.00	35	-100–400	Oz	-5.29	175b	150–200
Sulykos_2014 [112]	14	3-ring concentric annuli	2.29	2.29	Blue-violet	Visual	Fixation cross	6.3 cd/m2	400	***348***	0.20	2	Reverse roles	none	Average	rejection; EOG/EEG threshold exclusion	0.10		-100–400	Posterior	-0.64	201	180–220
Jack_2017 [113]	10	Annular sine-wave grating	3.20	3.20	Black	Visual	Report binocular rivalry	14.51 cd/m2	100	100	0.06	10	Reverse roles	none	Average	rejection; EOG/EEG threshold exclusion	0.10	40	-100–400	Right posterior	-1.60a	250	230–274
Sulykos_2018 [114]	15	LoVF Peripheral Checkerboard pattern	2.00	6.00	Grey	Visual	Fixation dot	81.3 cd/m2	***480***	***560***	0.20	2	Reverse roles	none	Nose-tip	rejection; EOG/EEG threshold exclusion	0.10	30	-100–500	O2	-3.26	167	154–174
Petro_2021 [115]	20	Peripheral Checkerboard patterns	2.00	6.00	Grey	Visual	Fixation dot	81.3	***480***	***580***	0.20	2	Reverse roles	none	Nose-tip	rejection; EOG/EEG threshold exclusion	0.10	30	-100–500	Occipital	-2.28a	129a	100–150
Nie_2021 [116]	30	Peripheral Wifi symbols	3.68	3.42	White	Visual	Fixation cross	grey-black	100	500	0.20	2	Reverse roles	none	Nose-tip	rejection; EOG/EEG threshold exclusion		30	-100–500	Occipital	-0.65	195a	170–220
**Spatial Frequency**																							
Tales_1999 [Experiment 1] [117]	12	Peripheral white vertical bars	0.68	2.20	Black	Visual	Fixation square	+0.6 cpd	200	***642***	0.06	2	none	none	Fz	rejection; EOG/EEG threshold exclusion		70	0–512	O2	-4.00a	325b	250–400
Tales_1999 [Experiment 2] [117]	12	Peripheral white vertical bars	0.68	2.20	Black	Visual	Fixation square	-0.6 cpd	200	***642***	0.06	2	none	none	Fz	rejection; EOG/EEG threshold exclusion		70	0–512	O1	-1.86a	325b	250–400
Tales_2002 [118]	24	Peripheral white vertical bars	0.68	2.20	Black	Visual	Fixation square	-0.6 cpd	200	***642***	0.06	2	none	none	Fz	rejection; EOG/EEG threshold exclusion		70	0–512	O2	-3.40a	325b	250–400
Heslenfeld_2003 [119]	14	Peripheral vertical square-wave gratings	16.00	5.60	Black	Visual	Visuo- motor task	1.72 cpd	17	***433***	0.20	1	Reverse roles	none	Average mastoids	correction; corrected via EOG regression	0.08	35	-60–452	Oz	-1.10	150b	100–200
Kenemans_2003 [120]	12	Peripheral vertical square-wave gratings	5.40	5.20	Black	Visual	Fixation cross	1.8 cpd	17	***450***	0.20		Deviant alone	none	Right mastoid	correction; corrected via EOG regression	0.05	40	-50–250	Oz	-1.19a	140a	60–200
Stagg_2004 [56]	12	Peripheral white vertical bars	0.34	2.23	Black	Visual	Fixation square	0.6 cpd	200	***642***	0.06		Reverse roles	none	Fz	rejection; EOG/EEG threshold exclusion	0.05	100	0–512	Occipital	-3.73a	305b	210–400
Maekawa_2005 [57]	7	Central windmill-pattern	5.80	5.80	Grey	Auditory and visual	Story and target windmill	18 vanes	200	800	0.10		Reverse roles	none	Nose-tip	rejection; EOG/EEG threshold exclusion	0.05	50	-72–542	Oz	-3.47a	245a	230–320
Tales_2006 [121]	10	Peripheral white vertical bars	0.68	2.20	Black	Visual	Fixation square	+0.6 cpd	200	***642***	0.06	2	none	none	Fz	rejection; EOG/EEG threshold exclusion		70	0–512	T6(P8)	-3.90a	300b	250–400
Tales_2008 [122]	10	Peripheral white vertical bars	0.68	2.20	Black	Visual	Fixation square	+0.6 cpd	200	***642***	0.06	2	none	none	Fz	rejection; EOG/EEG threshold exclusion		70	0–512	O2	-0.63a	325b	250–400
Maekawa_2009 [Experiment 1] [123]	10	Central windmill-pattern	5.80	5.80	Grey	Auditory	Story	18 vanes	200	800	0.10	7	Deviant block	none	EGI average [126, 127]	rejection; discarded epochs contaminated by EOG artifacts	0.50	30	-100–500	Oz	-6.33a	252a	150–300
Maekawa_2009 [Experiment 2] [123]	8	Central windmill-pattern	5.80	5.80	Grey	Auditory	Story	18 vanes	200	800	0.10	7	Standard block	none	EGI average [126, 127]	rejection; discarded epochs contaminated by EOG artifacts	0.50	30	-100–500	Oz	-2.19a	232a	150–300
Kenemans_2010 [124]	16	Peripheral vertical square-wave gratings	5.40	5.20	Black	Visual	Fixation cross	1.8 cpd	17	***450***	0.10		Reverse roles	none	Right mastoid	rejection; EOG/EEG threshold exclusion	0.05	30	-50–550	Oz	-0.50a	160b	150–170
Chang_2011 [125]	14	Peripheral white vertical bars	0.34	2.23	Black	Visual	Fixation square	0.6 cpd	200	612	0.12	2	Reverse roles	Equi- probable	Nose-tip	correction; corrected via EOG regression	0.10	30	-100–500	Parieto- occipital	-1.25a	200b	150–250
Maekawa_2011 [126]	9	Central windmill-pattern	5.80	5.80	Grey	Auditory	Story	18 vanes	200	800	0.10		Reverse roles	none	EGI average [126, 127]	rejection; discarded epochs contaminated by EOG artifacts	0.50	30	-100–500	Oz	-2.40	274	150–350
Sulykos_2011 [69]	12	Peripheral Gabor patches	0.80	0.80	Black	Visual	Spaceship	4 cpd	80	480	0.18		Reverse roles	none	Linked- mastoids	rejection; EOG/EEG threshold exclusion	0.10	30	-100–400	Oz	-1.18	136	135–145
Stothart_2013a [127]	17	Peripheral white vertical bars	10.50	10.50	White	Visual	Small Blue Frame	0.6 cpd	200	***642***	0.18	2	none	none	Average	correction; BESA automatic artifact correction		40	-100–500	Parieto- occipital	-1.15a	230a	170–264
Stothart_2013b [128]	39	Peripheral white vertical bars	1.37	4.47	Black	Visual	Fixation square	0.6 cpd	200	***642***	0.06	2	none	none	Average	correction; BESA automatic artifact correction		40	-300–600	Parieto- occipital	-0.66a	222a	100–600
Maekawa_2013 [129]	20	Central windmill-pattern	5.80	5.80	Grey	Auditory	Story	18 vanes	200	800	0.10		Reverse roles	none	EGI average [126, 127]	rejection; EOG/EEG threshold exclusion	0.05	30	-100–500	Oz	-1.25a	280b	150–350
Cleary_2013 [130]	20	Background horizontal square-wave gratings	10.88	14.48	Stimulus	Visual	Fixation cross	6 cpd	750	1,000	0.15		none	none	Right mastoid	correction; corrected via EOG regression	0.05	30	-200–1000	O1	-2.06a	162	130–200
Stothart_2014 [131]	26	Peripheral white vertical bars	1.50	4.46	Black	Visual	Fixation square	0.6 cpd	200	***642***	0.06		none	none	Average	correction; BESA automatic artifact correction		40	-100–500	Left parieto- occipital	-1.25a	195a	170–250
Hedge_2015 [Deviant 1] [132]	20	Peripheral vertical square-wave gratings	0.34	2.23	Black	Visual	Fixation square	+0.6 cpd	200	***700***	0.06	2	Reverse roles	none	Average	correction; BESA automatic artifact correction		40	-100−600	Parieto- occipital	-0.94	265b	161–329
Hedge_2015 [Deviant 2] [132]	20	Peripheral vertical square-wave gratings	0.34	2.23	Black	Visual	Fixation square	-0.6 cpd	200	***700***	0.06	2	Reverse roles	none	Average	correction; BESA automatic artifact correction		40	-100−600	Parieto- occipital	‐0.22		161–329
File_2017 [Experiment 2] [86]	23	Central windmill-pattern	13.82	13.82	Grey	Visual	Spaceship	6 vanes	200	800	0.10	7	Reverse roles	Equi- probable	Average	rejection; EOG/EEG threshold exclusion	0.10	30	-100–400	Occipital	-1.49	269	200–340
Bodnár_2017 [87]	19	Central windmill pattern	13.82	13.82	Grey	Visual	Centre tracking task	6 vanes	100	400	0.12		Reverse roles	none	Nose-tip	rejection; EOG/EEG threshold exclusion	0.10	30	-100–400	Occipital	-1.73	203	198–218
Male_2020 [Experiment 2] [34]	24	Central Gabor patches	3.00	3.00	Grey	Visual	Fixation dot	0.3 cpd	100	400	0.13	1	Cascadic	Cascadic	Nose-tip	correction; ICA + Eye-tracking	0.10	40	-100–400	P8	1.16		200–250
Dang_2023 [133]	31	Peripheral windmill-patterns			Grey	Visual	Fixation cross	+6 vanes	300	***600***	0.20		none	none	Average	correction; ICA	1.00	30	-200–500	O2	-0.91a	257a	230–330
Molnár_2024 [Deviant 1] [134]	39	Central windmill-pattern	32.28	16.14	Grey	Visual	Spaceship	+6 vanes	200	600	0.16	3	Reverse roles	none	Average	rejection; eliminated by ADJUST plugin	0.03	40	-100−600	Right occipital	0.15a	180b	160–200
Molnár_2024 [Deviant 2] [134]	39	Central windmill-pattern	32.28	16.14	Grey	Visual	Spaceship	-6 vanes	200	600	0.16	3	Reverse roles	none	Average	rejection; eliminated by ADJUST plugin	0.03	40	-100−600	Right occipital	0.08a		260–300
**Colour**																							
Horimoto_2002 [135]	11	Central coloured square				Auditory	Tones	blue-green blue	1,000	***1*,*750***	0.10		none	none	Linked- earlobes	rejection; EOG/EEG threshold exclusion + visual inspection	0.05	50	-100–900	Pz	-4.80	250	0–550
Czigler_2002 [Deviant 1] [58]	8	Peripheral square-wave grating	10.90	14.50	Stimulus	Visual	Fixation cross	red-pink	17	***450***	0.12		Reverse roles	Equi- probable	Nose-tip	rejection; EOG/EEG threshold exclusion	0.10	30	-100–650	Occipital	‐0.07a		128–142
Czigler_2002 [Deviant 2] [136]	8	Peripheral square-wave grating	10.90	14.50	Stimulus	Visual	Fixation cross	red-green	17	***450***	0.12		Reverse roles	Equi- probable	Nose-tip	rejection; EOG/EEG threshold exclusion	0.10	30.0	-100–650	Occipital	-0.36a	136	128–142
Czigler_2004 [137]	12	Peripheral coloured-black checkerboard			Dark	Visual	Fixation cross	green-red	17	***400***	0.10		Reverse roles	none	Linked- mastoids	none	0.10	30	-100–450	Oz	-1.36	170b	140–200
Mao_2005 [138]	25	Central coloured dots	2.86	2.86	Black	Visual	Dot colour	red-green, purple-white	300	700	0.25		none	none	Linked- earlobes	rejection; excluded trials containing EOG artifacts	0.05	100	-200–2200	P3	-3.00a	304	250–350
Kimura_2006 [139]	12	Peripheral discs	2.50	2.50	Black	Visual	Fixation shape	red-blue	70	350	0.20		Reverse roles and deviant block	none	Nose-tip	rejection; EOG/EEG threshold exclusion	0.10	30	-50–400	PO8	-1.29a	160b	150–170[58]
Czigler_2007 [Experiment 1: long ISI] [140]	11	Peripheral coloured-black checkerboard	8.07	6.06	Stimulus	Visual	Fixation cross	green-red	14	160	0.20		none	none	Nose-tip	rejection; EOG/EEG threshold exclusion	0.15	30	-50–300	Oz	-1.32	132	112–152
Czigler_2007 [Experiment 1: short ISI] [140]	11	Peripheral coloured-black checkerboard	8.07	6.06	Stimulus	Visual	Fixation cross	green-red	14	0	0.20		none	none	Nose-tip	rejection; EOG/EEG threshold exclusion	0.15	30	-50–300	Oz	‐0.46		112–152
Czigler_2007 [Experiment 2: short ISI] [140]	11	Peripheral coloured-black checkerboard	8.07	6.06	Stimulus	Visual	Fixation cross	green-red	14	26	0.20		none	none	Nose-tip	rejection; EOG/EEG threshold exclusion	0.15	30	-50–300	Oz	-1.06	124	104–144
Czigler_2007 [Experiment 2: short ISI] [140]	11	Peripheral coloured-black checkerboard	8.07	6.06	Stimulus	Visual	Fixation cross	green-red	14	13	0.20		none	none	Nose-tip	rejection; EOG/EEG threshold exclusion	0.15	30	-50–300	Oz	0.19		104–144
Liu_2008 [141]	15	Central red square	3.70	3.70	Grey	Visual	Target flower image	red-lighter red	100	900	0.20	2	none	none	Nose-tip	rejection; EOG/EEG threshold exclusion	0.05	30	-100−600	Posterior areas	-0.59	179	110–180
Grimm_2009 [142]	16	Peripheral green triangles	4.20	3.40	Grey	Visual	Fixation target	green-red	150	1,450	0.11	2	Reverse roles	none	Nose-tip	rejection; EOG/EEG threshold exclusion	0.75	35	-100–900	Right occipital	-0.87	280b	265–295
Thierry_2009 [143]	20	Central disc	2.00	2.00	Grey	Visual	Shape change	green-green or blue-blue	200	800	0.10	3	Reverse roles	none	Average	correction; EOG mathematical correction	0.10	20	-100–1000	Parieto- occipital	-0.91a	197a	162–232
Berti_2009 [144]	8	Peripheral triangle			Grey	Visual	Triangle orientation	red-green	200	1,100	0.18	3	Reverse roles	none	Nose-tip	rejection; excluded epochs with excessive EOG activity	1.00	25	-200–400	P3	0.52a		150–210
Czigler_2010 [68]	24	Peripheral line segments	0.04	0.37	Grey	Visual	Central line segment	turquoise-yellow-green	70	280	0.15		Reverse roles	none	Average mastoids	rejection; EOG/EEG threshold exclusion	0.15	30	-100–500	POz	-0.60	141	137–145
Clifford_2010 [Deviant 1] [27]	18	LoVF Peripheral squares	4.09	4.09	Grey	Visual	Fixation cross	blue-blue	200	***1*,*200***	0.20	1	none	none	Average earlobes	rejection; EOG threshold exclusion	0.10	40	-100–700	Posterior	‐0.27a		100–250
Clifford_2010 [Deviant 2] [27]	18	LoVF Peripheral squares	4.09	4.09	Grey	Visual	Fixation cross	blue-green	200	***1*,*200***	0.20	1	none	none	Average earlobes	rejection; EOG threshold exclusion	0.10	40	-100–700	Posterior	-1.76a	200b	100–250
Mo_2011 [145]	30	Peripheral squares	2.50	2.50	Grey	Visual	Fixation cross	blue-green	200	900	0.20		Reverse roles	none	Nose-tip	correction; EOG mathematical correction	0.80	20	-100–800	Parieto- occipital	-1.03a	160	130–190
Stefanics_2011 [Experiment 1]	13	Peripheral discs			Grey	Visual	Fixation cross	red-green	100	***800***	0.10		Reverse roles	none	Average	rejection; EOG/EEG threshold exclusion	0.10	30	-100–500	Occipital	-0.66	248a	232–254
Stefanics_2011 [Experiment 2]	15	Peripheral discs			Grey	Visual	Fixation cross	red-green	100	***800***	0.20		Reverse roles	none	Average	rejection; EOG/EEG threshold exclusion	0.10	30	-100–500	Occipital	-1.16	214a	142–286
Müller_2012 [146]	15	LoVF Peripheral squares	0.92	0.92	Grey	Visual	Fixation cross	green-red	120	600	0.10	1	Reverse roles	none	Nose-tip	rejection; EOG/EEG threshold exclusion	1.00	35	-100–600	Occipital	-1.57	260	240–280
Shi_2013 [73]	12	Peripheral red rectangles	0.10	0.30	White	Visual	Fixation cross	red-blue	50	550	0.10	1	none	none	Nose-tip	correction; corrected via EOG regression	0.10	100	-100–400	Occipito- temporal	-0.59a	200a	150–250
Wu_2013 [147]	12	Peripheral rectangles	3.44	1.15		Visual	Central number task	red-green	50	550	0.10	1	none	none	Nose-tip	correction; EOG artifact correction	0.10	100	-100–400			164	150–200
He_2014 [Deviant 1] [148]	12	Peripheral green circles	4.20	4.20	White	Visual	Fixation cross	red-green	100	400	0.09	2	none	none	Nose-tip	correction; corrected via EOG regression	0.05	30	-50–400	Left parieto- occipital	-2.03	187a	172–194
He_2014 [Deviant 2] [148]	12	Peripheral green circles	4.20	4.20	White	Visual	Fixation cross	green-sea-green	100	400	0.09	2	none	none	Nose-tip	correction; corrected via EOG regression	0.05	30	-50–400	Left parieto- occipital	0.06		172–194
Zhong_2015 [Deviant 1 RVF] [149]	26	RVF Peripheral squares	1.27	1.27	Grey	Visual	Fixation cross	BLUE-GREEN	200	900	0.20	1	none	none	Nose-tip	correction; EOG mathematical correction	0.80	20	-100–800	Parieto- occipital	-0.46a	160	130–190
Zhong_2015 [Deviant 2 RVF] [149]	26	RVF Peripheral squares	1.27	1.27	Grey	Visual	Fixation cross	blue-green	200	900	0.20	1	none	none	Nose-tip	correction; EOG mathematical correction	0.80	20	-100–800	Parieto- occipital	‐0.09a		130–190
Zhong_2015 [Deviant 1 LVF] [149]	26	LVF Peripheral squares	1.27	1.27	Grey	Visual	Fixation cross	BLUE-GREEN	200	900	0.20	1	none	none	Nose-tip	correction; EOG mathematical correction	0.80	20	-100–800	Parieto- occipital	‐0.18a		130–190
Zhong_2015 [Deviant 2 LVF] [149]	26	LVF Peripheral squares	1.27	1.27	Grey	Visual	Fixation cross	blue-green	200	900	0.20	1	none	none	Nose-tip	correction; EOG mathematical correction	0.80	20	-100–800	Parieto- occipital	‐0.18a		130–190
Sysoeva_2015 [UVF short ISI] [150]	12	UVF Peripheral discs	4.23	4.23	Black	Visual	Line orientation within discs	red-green		***1*,*100***	0.10		Reverse roles	none	Linked- mastoids	rejection; EOG/EEG threshold exclusion	1.00	30	-100–500	O1	-1.01a	141	120–160
Sysoeva_2015 [LoVF short ISI] [150]	12	LoVF Peripheral discs	4.23	4.23	Black	Visual	Line orientation within discs	red-green		***1*,*100***	0.10		Reverse roles	none	Linked- mastoids	rejection; EOG/EEG threshold exclusion	1.00	30	-100–500	O1	‐0.21a		120–160
Sysoeva_2015 [UVF long ISI] [150]	12	UVF Peripheral discs	4.23	4.23	Black	Visual	Line orientation within discs	red-green		***1*,*610***	0.10		Reverse roles	none	Linked- mastoids	rejection; EOG/EEG threshold exclusion	1.00	30	-100–500	O1	‐0.13a		120–160
Sysoeva_2015 [LoVF long ISI] [150]	12	LoVF Peripheral discs	4.23	4.23	Black	Visual	Line orientation within discs	red-green		***1*,*610***	0.10		Reverse roles	none	Linked- mastoids	rejection; EOG/EEG threshold exclusion	1.00	30	-100–500	O1	‐0.10a		120–160
Li_2017 [85]	12	Peripheral polygons	0.57	1.70	Grey	Manual	Visuo-motor task	green-red	50	600	0.17	1	Equi- probable	Equi- probable		correction; removed influence of EOG on other leads			-100–400	Right occipito- temporal	0.43a	224a	175–225
Maciejewska_2017 [151]	9	Central checkerboards			Stimulus	Visual	Fixation cross	red-green/ additional stimulus	20	400	0.10	4	Equi- probable	Equi- probable	AFz	rejection; EOG/EEG threshold exclusion	1.00	30.0	-100–400	Cp1	-1.18	165b	160–170
Stefanics_2018 [152]	34	Peripheral faces	3.80	5.40	Grey	Visual	Fixation cross	red-green	200	***700***	0.50	5	Reverse roles	none	Average	correction; BESA automatic artifact correction	0.05	30	-100–500	Occipito- temporal	-0.64a	200a	196–228
Hilger_2023 [153]	50	Peripheral matrix of discs	9.76	8.82	Black	Visual	Fixation cross	red-green	300	300	0.20	1	Reverse roles	none	Average	rejection; EOG threshold exclusion	0.10	10	-100–400	POz	-0.12	221a	125–275
Suzuki_2024 [154]	39	Five Chevrons	11.42	2.43	Grey	Visual	Middle chevron orientation	black-red	100	***1*,*500***	0.20	1	Reverse roles	Equi- probable	Nose-tip	correction; ICA	0.10	50	-100–800	Posterior clusters	-0.85	205b	180–230
Qian_2014 [79]	14	Peripheral red rectangles	0.10	0.30	White	Visual	Fixation cross	red-blue	50	550	0.10	1	none	none	Nose-tip	rejection; EOG/EEG threshold exclusion	0.10	100	-100–500	Occipito- temporal	-1.11	205a	150–250
**Size/shape**																							
Czigler_1990 [No attention condition] [61]	9	Chevrons within rectangular frame	1.27	0.77		Visual	Central chevrons	+Frame thickness	83	417	0.20	2	none	none	Linked- earlobes	rejection; EOG threshold exclusion	0.20	50.0	0–768	Oz	‐0.09a		210–270
Czigler_1990 [Attention condition] [61]	9	Chevrons within rectangular frame	1.27	0.77		Visual	Frame deviants	+Frame thickness	83	417	0.20	2	none	none	Linked- earlobes	rejection; EOG threshold exclusion	0.20	50	0–768	Oz	‐1.94a		210–270
Woods_1992 [Attention condition] [155]	12	Peripheral vertical gratings	3.90	4.40	Black	Visual	Visual deviants	-0.80° height	50	***400***	0.10		none	none	Non-cephalic	correction; EOG artifact correction+CCTV	0.10	40	-200–800	PTc	-1.79a	260b	220–300
Woods_1992 [No attention condition] [155]	12	Peripheral vertical gratings	3.90	4.40	Black	Auditory and Visual	Auditory deviants	-0.80° height	50	***400***	0.10		none	none	Non-cephalic	correction; EOG artifact correction+CCTV	0.10	40	-200–800	PTc	‐0.56a		230–330
Alho_1992 [26]	12	Central white vertical gratings	3.90	4.40	Black	Auditory and Visual	Deviants	-0.50° height	50	***400***	0.20		Deviant alone	none	Non-cephalic	rejection; excluded trials with excessive eye movements	0.10	40	-200–800	Oz	-2.00a	120	90–290
Kaplan_2007 [No attention condition] [156]	13	Peripheral pattern of red light-emitting diodes	9.20	18.20		Visual	Fixation red light-emitting diode	-4.6° width			0.14		Reverse roles	none	Linked- earlobes	none			-100−700	P4	0.00		340–460
Kaplan_2007 [Attention condition] [156]	13	Peripheral pattern of red light-emitting diodes	9.20	18.20		Visual	Fixation red light-emitting diode	-4.6° width			0.14		Reverse roles	none	Linked- earlobes	none			-100−700	P4		400	340–460
Kimura_2008 [Deviant 1] [157]	12	Peripheral discs	1.70	1.70	Black	Visual	Fixation target	+0.9° diameter	200	1,600	0.16		Deviant block	none	Nose-tip	rejection; EOG/EEG threshold exclusion	0.03	30	-100–900	Oz	-1.69a	130b	120–140
Kimura_2008 [Deviant 2] [157]	12	Peripheral discs	1.70	1.70	Black	Visual	Fixation target	-0.9° diameter	200	1,600	0.16		Deviant block	none	Nose-tip	rejection; EOG/EEG threshold exclusion	0.03	30	-100–900	Oz	0.01a		120–140
Grimm_2009 [142]	16	Peripheral green triangles	4.20	3.40	Grey	Visual	Fixation target	Hexagon	150	1,450	0.11	2	Reverse roles	none	Nose-tip	rejection; EOG/EEG threshold exclusion	0.75	35	-100–900	Right occipital	-0.70	260b	245–275
Froyen_2010[158]	16	Central letter	1.20	0.80	Black	Visual	Target image	letter-non-letter	500	750	0.10		none	none	Nose-tip	correction; corrected for vertical eye movements	1.00	30	-50–800	Oz	-3.30	273	248–334
Bubrovszky_2011 [159]	10	Central“O”				Visual	Fixation target	“X”	200	***1*,*200***	0.10		none	none		rejection; inspection for artifacts exclusion	0.10	45	-100–700	Posterior		208b	185–232
Cléry_2013 [160]	13	Central ellipse	2.00	2.00	Black	Visual	Fixation cross	Horizontal-vertical ellipse	140	650	0.18		Reverse roles	none	Nose-tip	correction; NeuroScan spatial filter transform	0.30	70	-100–600	Right occipito- parietal- temporal	-1.60	210	180–240
Shi_2013 [Shape deviant] [73]	12	Peripheral red rectangles	0.10	0.30	White	Visual	Fixation cross	red-ellipses	50	550	0.10	1	none	none	Nose-tip	correction; corrected via EOG regression	0.10	100	-100–400	Occipito- temporal	-1.00a	180a	150–250
Shi_2013 [Size deviant] [73]	12	Peripheral red rectangles	0.10	0.30	White	Visual	Fixation cross	+0.06° width	50	550	0.10	1	none	none	Nose-tip	correction; corrected via EOG regression	0.10	100	-100–400	Occipito- temporal	-0.60	190a	150–250
Wu_2013 [Shape deviant] [74]	12	Peripheral rectangles	3.44	1.15		Visual	Central number task	circle	50	550	0.10	1	none	none	Nose-tip	correction; EOG artifact correction	0.10	100	-100–400			174	150–200
Wu_2013 [Size deviant] [74]	12	Peripheral rectangles	3.44	1.15		Visual	Central number task	width	50	550	0.10	1	none	none	Nose-tip	correction; EOG artifact correction	0.10	100	-100–400			162	150–200
Bottari_2014 [161]	12	Circle	2.00	2.00	Black	Visual	Fixation cross	circle-ellipse	107	580	0.14	3	Reverse roles	none	Nose-tip	correction; ICA	0.10	40	-200–600	Right posterior	-0.64a	193	157–292
Qian_2014 [Shape deviant] [79]	14	Peripheral red rectangles	0.10	0.30	White	Visual	Fixation cross	rectangles-ellipses	50	550	0.10	1	none	none	Nose-tip	rejection; EOG/EEG threshold exclusion	0.10	100	-100–500	Occipito- temporal	-0.64	200a	150–250
Qian_2014 [Size deviant] [79]	14	Peripheral red rectangles	0.10	0.30	White	Visual	Fixation cross	+0.06° width	50	550	0.10	1	none	none	Nose-tip	rejection; EOG/EEG threshold exclusion	0.10	100	-100–500	Occipito- temporal	-0.55	180a	150–250
Li_2017 [85]	12	Peripheral polygons	0.57	1.70	Grey	Manual	Visuo-motor task	rectangle-hexagon	50	600	0.17	1	Equi- probable	Equi- probable		correction; removed influence of EOG on other leads			-100–400	Right occipito- temporal	0.30a	220a	175–225
Maciejewska_2017 [151]	9	Central checkerboards			Stimulus	Visual	Fixation cross	+1.5°	20	400	0.10	4	Equi- probable	Equi- probable	AFz	rejection; EOG/EEG threshold exclusion	1.00	30.0	-100–400	POz	-0.84	170b	140–200
Yu_2017 [Deviant 1] [162]	23	RVF Peripheral novel polygons			White	Visual	Fixation cross	Most different polygon	200	900	0.20	1	none	none	Nose-tip	rejection; EOG/EEG threshold exclusion	0.80	40	-100–800	Lateral parieto- occipital	-0.76a	160b	130–190
Yu_2017 [Deviant 2] [162]	23	RVF Peripheral novel polygons			White	Visual	Fixation cross	Least different polygon	200	900	0.20	1	none	none	Nose-tip	rejection; EOG/EEG threshold exclusion	0.80	40	-100–800	Lateral parieto- occipital	‐0.10a		130–190
Kojouharova_2019 [163]	19	Peripheral matrix of black lines or shapes	1.74	1.74	Grey	Visual	Central tracking task	Shape-bar	200	400	0.20	2	Reverse Roles	Equi- probable	Average	rejection; EOG/EEG threshold exclusion	0.10	30	-100–500	Oz	-0.92a	124.4	122–178
Schlossmacher_2020 [164]	52	Peripheral white lines forming square/rectangle	10.00	10.00	Black	Visual	Shape omissions	Square-rectangle	100	700	0.10	4	Reverse Roles	none	Average	rejection; visual inspection	0.10	25	-200–600	Posterior	-0.57a	261a	200–300
Nie_2020 [165]	30	Peripheral Wifi symbols	3.68	3.42	White	Visual	Fixation cross	rounded-horizontal bars	100	500	0.20	3	Reverse roles	none	Nose-tip	rejection; EOG/EEG threshold exclusion	0.05	100	-100–500	Occipital	-0.44	220a	180–300
**Location**																							
Berti_2004 [Experiment 1] [166]	10	Green square with central grey triangles			Grey	Visual	Stimulus duration	moved within green square	200	1,100	0.12		none	none	Nose-tip	rejection; excluded epochs with excessive EOG activity	1.00	20	-200–400	P8	-1.19	224a	220–300
Berti_2004 [Experiment 2] [166]	6	Green square with central grey triangles			Grey	Visual	Stimulus duration	moved within green square	200	1,100	0.12		Equi- probable	Equi- probable	Nose-tip	rejection; excluded epochs with excessive EOG activity	1.00	20	-200–400	P8	-1.50	245a	220–300
Nagai_2005 [167]	14	Four white capital letters	0.29	0.23	Black	Visual	Letters	0.63° lateral shift	150	40	0.33	2	none	none	Linked- earlobes	rejection; visual inspection		50	-480−800	Pz		350b	300–400
Berti_2006 [168]	8	Green square with central grey triangles			Grey	Visual	Stimulus duration	moved within green square	200	1,300	0.12		Position Equi- probable	Position Equi- probable	Nose-tip	rejection; excluded epochs with excessive EOG activity	1.00	20	-200–600	Posterior occipital	-2.01	220	170–300
Grimm_2009 [142]	16	Peripheral green triangles	4.20	3.40	Grey	Visual	Fixation target	2.4° Vertically shifted	150	1,450	0.11	2	Reverse roles	none	Nose-tip	rejection; EOG/EEG threshold exclusion	0.75	35	-100–900	Right occipital	-0.93	245b	230–260
Boll_2009 [169]	11	Peripheral green triangles			Grey	Auditory and Visual		0.42°↑	200	***1*,*700***	0.19		none	none	Nose-tip	rejection; EOG/EEG threshold exclusion	1.00	20	-200–800	P8	-0.86	200	170–250
Berti_2009 [Experiment 1] [144]	8	Peripheral green triangle			Grey	Visual	Triangle orientation	Laterally shifted	200	1,300	0.18	3	Reverse roles	none	Nose-tip	rejection; excluded epochs with excessive EOG activity	1.00	25	-200–400	P3	-2.74	190a	150–210
Berti_2009 [Experiment 2] [144]	8	Central green triangle			Grey	Visual	Number task	Laterally shifted	200	1,300	0.18	3	Reverse roles	none	Nose-tip	rejection; excluded epochs with excessive EOG activity	1.00	25	-200–400	P3	-2.37	200a	150–210
Berti_2011 [170]	10	Upper-case consonants			Grey	Visual	Target detection	Vertically shifted	100		0.40	5	none	none	Nose-tip	rejection; excluded epochs with extensive eye movements	1.00	30	-100–400	O2	-2.36	280a	260–300
He_2014 [148]	12	Peripheral green circles	4.20	4.20	White	Visual	Fixation cross	90°	100	400	0.09	2	none	none	Nose-tip	correction; corrected via EOG regression	0.05	30	-50–400	Right parieto- occipital	-3.54	199a	196–218
Berti_2018 [LVF] [171]	12	LVF Peripheral (2.86°) digits			Grey	Visual	Fixation cross	Outward Peripherally	200	1,100	0.12	3	none	none	Nose-tip	rejection; excluded epochs with excessive EOG activity	1.00	25	-200–600	Right parieto- occipital	‐0.41		200–250
Berti_2018 [RVF] [171]	12	RVF Peripheral (2.86°) digits			Grey	Visual	Fixation cross	Outward Peripherally	200	1,100	0.12	3	none	none	Nose-tip	rejection; excluded epochs with excessive EOG activity	1.00	25	-200–600	Left parieto- occipital	-0.65	225b	200–250
**Motion**																							
Lorenzo-López_2004 [172]	7	Peripheral horizontal sinusoidal grating	4.13	4.13		Visual	Fixation number	1.95°/s OD	133	665	0.20	1	none	none	Nose-tip	rejection; visual inspection	0.10	30	-100–700	Parieto- occipital	-2.26	180a	165–205
Pazo-Alvarez_2004a [173]	7	Peripheral sinusoidal grating	4.13	4.13		Visual	Fixation number	1.95°/s OD	133	665	0.20	1	Reverse roles	Equi- probable	Nose-tip	rejection; EOG/EEG threshold exclusion	0.10	30	-100–700	Occipital	-1.78a	155b	145–165
Pazo-Alvarez_2004b [174]	12	Peripheral sinusoidal grating	4.13	4.13		Visual	Fixation number	1.95°/s OD	133	665	0.20	1	Reverse roles	none	Nose-tip	rejection; visual inspection	0.05	100	-50–500	Occipito- temporal	-0.56a	150a	100–225
Kremláček_2006 [175]	9	Peripheral horizontal sinusoidal grating	42.00	30.00	Stimulus	Visual	Fixation target	50°/s OD	200	600	0.06	3	none	none	Right earlobe	correction; PCA	0.30	30	0–500	Occipital	-2.72a	191a	145–260
Amenedo_2007 [176]	12	LoVF Peripheral sinusoidal grating	4.13	4.13	Stimulus	Visual	Fixation number	1.95°/s OD	133	665	0.20	1	none	none	Nose-tip	rejection; excluded trials with eye blinks	0.10	30	-50–500	Posterior	-3.31	198b	145–225
Hosak_2008 [177]	17	Peripheral sinusoidal grating	42.00	30.00	Stimulus	Visual	Fixation target	50°/s OD	200	600	0.06	3	none	none	Right earlobe	correction; PCA	0.30	30	0–1000	Pz	-1.46a	180b	120–240
Urban_2008 [178]	24	Peripheral horizontal sinusoidal gratings	40.00	30.00	Stimulus	Visual	Central grating motion	50°/s ↓ peripheral motion	200	600	0.06		none	none	Right earlobe	none; CCD camera	0.30	30	0–1000	Pz	-1.41a	150b	100–200
Langrová_2012 [179]	21	Peripheral horizontal sinusoidal grating	37.00	28.00	Black	Visual	Central motion target	50°/s OD	500	1,000	0.06		none	none	Right earlobe	rejection; visual inspection	0.30	100		Pz		180b	120–240
Kuldkepp_2013 [180]	40	Peripheral vertical sinusoidal gratings	27.60	20.50	Stimulus	Visual	Target motion	1.6°/s OD	200	600	0.15	1	none	none	Linked- earlobes	rejection; EOG/EEG threshold exclusion	1.00	30	-100–600	Parietal	-0.47	167b	150–175
Kremláček_2013 [181]	12	Peripheral sinusoidal grating	47.00	36.00	Stimulus	Visual	Central number changes	12.5–25°/s OD	100	600	0.17		Reverse Roles	none	Right earlobe	rejection; EOG/EEG threshold exclusion	0.05	30	-99–400	Centro- parietal	-0.68	172a	142–198
Tugin_2016 [182]	18	Peripheral white circular spots	2.65	2.65	Black	Visual	Fixation cross	42rpm OD	1,400	***2*,*800***	0.14		none	none	Average	rejection; EOG/EEG threshold exclusion	0.50	40	-100–600	Occipital	‐0.23a		100–400
Schmitt_2018 [183]	8	Gabor pattern	3.20		Grey	Visual	Central or peripheral Gabor pattern	9.4° left or right	190	***210***	0.20		Reverse roles	Equi- probable	Average- mastoids	rejection; excluded trials with blinks or eye movements		50	-200–900	Parieto- occipital	-0.46a	160	110–190
Rowe_2020 [184]	19	Peripheral dot array	30.00	30.00	Black	Visual	Central letter	OD shift	500	0		5	none	none	Average	rejection; EOG/EEG threshold exclusion	0.50	40	-100–400	Left posterior	-0.19a	150	140–160
Schmitt_2021 [185]	12	Peripheral moving white dots	42.00	10.90	Black	Visual	Fixation dot	10° left or right	***350***	800	0.20		Reverse roles	none	Average- mastoids	rejection; excluded trials with blinks or eye movements	3.00	80	-50–500	Pz	-1.13a	135	110–160
Intartaglia_2022 [186]	22	4x4 blue LEDs on 12x16 LED panel	4.20	4.20	Light-emitting diode (LED) panel		Count events	moved ↓ vs. ↑	130	508	0.14	4	none	none	Average- mastoids	correction; ICA	0.10	40	-50–588	Posterior	-2.11a	182a	158–198
**Duration/omission**																							
Mao_2004 [187]	15	Central white spot	1.10	1.10		Visual	Stimulus duration	+300ms	300	1,100	0.25		none	none	Linked- earlobes	rejection; excluded trials with EOG artifacts	0.05	100	-200–2600	C3	-2.40	171b	156–185
Czigler_2006 [Experiment 1: short ISI] [188]	10	LoVF Peripheral matrix of hollow squares	5.24	4.29	Grey	Visual	Fixation cross and stimulus omissions	-17 ms	17	92	0.10	4	none	none	Nose-tip	rejection; EOG/EEG threshold exclusion	0.10	30	-100–800	Oz	-2.56	167	100–200
Czigler_2006 [Experiment 1: long ISI 1] [188]	10	LoVF Peripheral matrix of hollow squares	5.24	4.29	Grey	Visual	Fixation cross and stimulus omissions	-17 ms	17	162	0.10	4	none	none	Nose-tip	rejection; EOG/EEG threshold exclusion	0.10	30	-100–800	Oz	‐0.22		100–200
Czigler_2006 [Experiment 1: long ISI 2] [188]	10	LoVF Peripheral matrix of hollow squares	5.24	4.29	Grey	Visual	Fixation cross and stimulus omissions	-17 ms	17	194	0.10	4	none	none	Nose-tip	rejection; EOG/EEG threshold exclusion	0.10	30	-100–800	Oz	0.46		100–200
Czigler_2006 [Experiment 2: short ISI] [188]	12	LoVF Peripheral matrix of hollow squares	5.24	4.29	Grey	Visual	Fixation cross	-17 ms	17	58	0.10	4	none	none	Nose-tip	rejection; EOG/EEG threshold exclusion	0.10	30.0	-300–500	O2	-2.83	206b	186–226
Czigler_2006 [Experiment 2: long ISI 1] [188]	12	LoVF Peripheral matrix of hollow squares	5.24	4.29	Grey	Visual	Fixation cross	-17 ms	17	162	0.10	4	none	none	Nose-tip	rejection; EOG/EEG threshold exclusion	0.10	30	-300–500	O2	‐0.21		188–226
Czigler_2006 [Experiment 2: long ISI 2] [188]	12	LoVF Peripheral matrix of hollow squares	5.24	4.29	Grey	Visual	Fixation cross	-17 ms	17	194	0.10	4	none	none	Nose-tip	rejection; EOG/EEG threshold exclusion	0.10	30	-300–500	O2	0.16		188–226
Khodanovich_2010 [Deviant 1] [189]	10	Green light- emitting diodes	1.15	1.15		Visual	Deviant	-50 ms	200	***2*,*000***	0.20		Deviant block	none	Average earlobes	rejection; visual inspection	0.16	25	-200–800	Posterior temporal	0.00		250–350
Khodanovich_2010 [Deviant 2] [189]	10	Green light- emitting diodes	1.15	1.15		Visual	Deviant	-150 ms	200	***2*,*000***	0.20		Deviant block	none	Average earlobes	rejection; visual inspection	0.16	25	-200–800	Posterior temporal	-1.75a	300	250–350
Chen_2010 [190]	13	Central red disc	2.29	2.29	Black	Auditory	Stimulus duration	-80 ms	200	***700***	0.15		Reverse roles	none	Nose-tip	rejection; EOG threshold exclusion	0.10	24	-100–600	Parieto- occipital	-1.27a	316b	296–336
Qiu_2011 [191]	20	Peripheral black squares	4.00	3.80		Visual	Fixation cross	-100 ms	150	***450***	0.20		Reverse roles	none	Nose-tip	correction; EOG mathematical correction	0.10	100	-100–400	Occipital	-2.74	222a	200–250
Shi_2013 [73]	12	Peripheral red rectangles	0.10	0.30	White	Visual	Fixation cross	50	50	550	0.10	1	none	none	Nose-tip	correction; EOG artifact correction	0.10	100	-100–400	Occipito- temporal	-0.65a	190a	150–250
Wu_2013 [74]	12	Peripheral rectangles	3.44	1.15		Visual	Central number task	+100 ms	50	550	0.10	1	none	none	Nose-tip	correction; corrected via EOG regression	0.10	100	-100–400			152	150–200
He_2014 [148]	12	Peripheral green circles	4.20	4.20	White	Visual	Fixation cross	+80 ms	100	400	0.09	2	none	none	Nose-tip	correction; corrected via EOG regression	0.05	30	-50–400	Left parieto- occipital	0.86	240a	220–242
Si_2014 [192]	15	Peripheral red rectangles	4.50	4.50		Visual	Fixation cross	+50 ms	50	500	0.20	2	none	none	Nose-tip	correction; EOG artifact correlation method	0.10	30	-100–600	Occipito- temporal	-1.10	216	200–250
Qian_2014 [79]	14	Peripheral red rectangles	0.10	0.30	White	Visual	Fixation cross	50	50	550	0.10	1	none	none	Nose-tip	rejection; EOG/EEG threshold exclusion	0.10	100	-100–500	Occipito- temporal	-0.69	133a	150–250
Li_2016a [193]	24	Peripheral Squares	7.40	7.40	Grey	Visual	Fixation cross	+100 ms	50	500	0.10	2	none	none	Nose-tip	correction; corrected via EOG regression	0.05	100	-100–400	Fz, Cz, O1, O2, and Oz	-3.95	208	100–300
Li_2016b [194]	40	Central Circular Checkerboard (CCC)	32.00	32.00	Stimulus	Visual	Deviant	Omitted CCC	100	1,000	0.15	2	none	none	Average- mastoids	correction; EOG artifact correction	0.10	30	-200–800	Occipito- temporal	-6.73	225a	220–270
Yang_2016 [Deviant 1] [195]	21	Peripheral black squares	4.00	3.80		Visual	Fixation cross	+100 ms	50	600	0.20	2	none	none	Nose-tip	correction; EOG artifact correction	1.00	30	-100–400	Occipital	-1.61	218	180–260
Yang_2016 [Deviant 2] [195]	21	Peripheral black squares	4.00	3.80		Visual	Fixation cross	-100 ms	150	600	0.20	2	none	none	Nose-tip	correction; EOG artifact correction	1.00	30	-100–400	Occipital	0.87		180–260
Durant_2018 [Decrement deviant 1] [196]	20	Peripheral moving white dots	0.15	0.15	Grey	Visual	Spaceship	-80 ms	200	***600***	0.17	3	Reverse roles	none	Average	rejection; EOG/EEG threshold exclusion + visual inspection	0.50	30	-100–400	Parieto- occipital	0.00		220–270
Durant_2018 [Motion decrement deviant 1] [196]	20	Peripheral moving white dots	0.15	0.15	Grey	Visual	Spaceship	-80 ms	120	***600***	0.17	3	Reverse roles	none	Average	rejection; EOG/EEG threshold exclusion + visual inspection	0.50	30	-100–400	Parieto- occipital	0.00		220–270
Durant_2018 [Motion decrement deviant 2] [196]	20	Peripheral moving white dots	0.15	0.15	Grey	Visual	Spaceship	-80 ms	120	***600***	0.17	3	Reverse roles	none	Average	rejection; EOG/EEG threshold exclusion + visual inspection	0.50	30	-100–400	Parieto- occipital	0.00		220–270
Durant_2018 [Motion increment deviant 1] [196]	20	Peripheral moving white dots	0.15	0.15	Grey	Visual	Spaceship	+80 ms	120	***600***	0.17	3	Reverse roles	none	Average	rejection; EOG/EEG threshold exclusion + visual inspection	0.50	30	-100–400	Parieto- occipital	-0.57	231a	220–270
Durant_2018 [Motion increment deviant 2] [196]	20	Peripheral moving white dots	0.15	0.15	Grey	Visual	Spaceship	+80 ms	120	***600***	0.17	3	Reverse roles	none	Average	rejection; EOG/EEG threshold exclusion + visual inspection	0.50	30	-100–400	Parieto- occipital	‐0.17		220–270
Yang_2019 [197]	15	Peripheral black Squares	3.80	4.00	White	Visual	Fixation cross	+100 ms	50	150	0.20	2	Reverse Roles	none	Nose-tip	correction; EOG artifact correction	1.00	30	-100–400	Occipital	-1.92	179a	150–300
**Phase**																							
Male_2020 [Experiment 2] [34]	24	Central Gabor patches	3.00	3.00	Grey	Visual	Fixation dot	1.2π radians	100	400	0.13	1	Cascadic	Cascadic	Nose-tip	correction; ICA + Eye-tracking	0.10	40	-100–400	P8	0.41		200–250

*Note*. SOI = stimulus(i) of interest, W = width, H = height, BG = background, D = Deviant, S = Standard, PC = physical control, AC = adaptation control, Ref = Reference, HP = high-pass, LP = low-pass, LoVF = Lower visual field, UVF = Upper visual field, RVF = Right visual field, LVF = Left visual field, OD = Opposite Direction

^a^ Value was calculated from figures depicting the study or condition within study.

^b^ Value represents the midpoint of the time window used to calculate mean amplitude.

^c^ Eye movement tracking performed during no EEG recording

Where there are multiple entries for the same deviant type, a condition or experiment is denoted in square brackets in the study identifier. There are 76 entries for orientation, 6 entries for contrast, 8 for luminance, 28 for spatial frequency, 39 for colour, 27 for shape/size, 12 for location, 15 for motion, 26 for duration/omission, and 1 entry for phase, for a total of 238 entries.

Mean amplitude was estimated for 107 entries. Peak latency was estimated for 110 entries. Overall, vMMNs with later peak latencies were larger than vMMNs with earlier peak latencies, *r*(158) = −0.242, *p* = .002 (two-tailed, listwise exclusion). The average mean amplitude for these 160 entries was −1.52±1.2 μV. The average peak latency was 205±51 ms. Mean and standard deviation of the number of healthy control participants for all entries was 17 (±8), ranging from 6–52.

### 3.2. Linear regression analysis

The linear regression equation which best explained variance in the mean amplitude data, *R*^2^ = .134, *F*(3,219) = 11.182, *p* < .001, contained:

- whether a control for adaptation was used (ß = .282, *p* < .001), with larger amplitudes where a control was absent,
- whether attention was towards vs. away from the SOI (ß = −.142, *p* = .027), with larger amplitudes where attention was on the SOI, and
- SOI duration (ß = −.139, *p* = .030), with larger amplitudes associated with longer SOI durations,

The regression equation which best explained the variance in peak latency data, *R*^2^ = .141, *F*(4,164) = 6.544, *p* < .001, contained:

- whether attention was towards vs. away from the SOI (ß = .210, *p* = .005), with later peak latencies where attention was on the SOI,
- ISI (ß = .202, *p* = .008), with later peak latencies associated with longer ISIs,
- the minimum number of standards between deviants (ß = .169, *p* = .024), with later peak latencies associated with increasing number of standards between stimuli, and
- deviant probability, with later peak latencies associated with less frequent deviants (ß = −.170, *p* = .024).

### 3.3. Deviant feature

**Table 2** shows the means and standard deviations for mean amplitude and peak latency and non-binary parameters and for each deviant feature. These are ISI, stimulus duration, deviant probability (as a proportion of 1), and the minimum number of standards (as integers—where the minimum number of standards was not specified, a value of 0 was given).

**Table 2:** Means (M) and standard deviations (σ) of mean VMMN amplitude and peak latency for each deviant feature in Table 1.

Feature	Stimulus Duration (ms)	Inter-stimulus-interval (ms)	Deviant (%)	Minimum N of Standard	Mean Amplitude (M±σ) μV	Peak Latency (M±σ) (ms)
Orientation	173±140	502±206	0.16±0.04	2.7±2.39	-0.75±0.94	202±48
Contrast	86±32	510±141	0.15±0.03	2.33±1.15	-0.43±1.07	160±11
Luminance	249±175	488±194	0.17±0.06	3.29±3.09	-2.58±1.86	208±55
Spatial Frequency	192±128	643±143	0.11±0.05	2.88±2.03	-1.69±1.57	238±62
Colour	141±172	751±454	0.16±0.07	1.71±1.15	-0.84±0.94	194±46
Shape or Size	119±101	703±372	0.14±0.04	1.82±1.07	-0.89±0.83	205±63
Location	175±40	1081±470	0.17±0.1	2.88±0.99	-1.69±0.99	234±47
Motion Direction	300±330	732±616	0.15±0.06	2.22±1.56	-1.34±0.99	168±18
Stimulus Duration or Omission	97±76	597±486	0.15±0.05	2.67±1.06	-1.16±1.71	211±48
Phase	100±0	400±0	0.13±0.00	1.00±0.00	0.41±0.00	
Total	165±157	633±378	0.15±0.06	2.47±1.85	-1.07±1.24	206±53

Although deviant feature did not emerge as a significant predictor of amplitude or latency data, a one-way analysis of mean amplitudes, removing the single entry for phase to allow for Tukey’s post-hoc, revealed a significant main effect of deviant feature on mean amplitude, *F*(8,227) = 4.265, *p* < .001, η^2^_p_ = .135, and peak latency, *F*(8,170) = 3.138, *p* = .003, η^2^_p_ = .135.

Overall amplitudes were significantly smaller for orientation compared to luminance (*p* = .001) and spatial frequency (*p* = .011). Luminance also produced significantly larger amplitudes than contrast (*p* = .024), colour (*p* = .006) and shape/size (*p* = .016).

Peak latency was significantly later for spatial frequency deviants than for colour (*p* = .046) and motion (*p =* .002). Peak latency was also significantly later for location deviants than for motion deviants (*p =* .037).

### 3.4. Stimulus duration and ISI

There was a main effect of deviant feature on SOI duration, *F*(8,230) = 3.163, *p* = .002, η^2^_p_ = .103, due to longer SOI duration for motion deviants compared to colour (*p* = .022), shape/size (*p* = .010), and duration deviants (*p* = .002). SOI duration emerged as a significant predictor in regression analysis of mean amplitude data only, with larger amplitudes associated with longer SOI duration.

There was an effect on ISI, *F*(8,234) = 4.526, *p* < .001, η^2^_p_ = .139, due to longer ISIs for location deviants compared with orientation (*p* < .001), contrast (*p* = .048), luminance (*p* = .012), spatial frequency (*p* = .019), and duration/omission deviants (*p* = .006). The colour vs. orientation difference in ISIs was also significant (*p* = .020). ISI emerged as a significant predictor in regression analysis of latency data only, with later vMMN peak latencies associated with longer ISIs.

### 3.5. Oddball deviant probability and minimum number of standards preceding a deviant

Only 5% (n = 11) of **Table 1** entries did not declare a deviant probability of 20% or less. Deviant probabilities did not differ across deviant features. Over half of the entries (63%, n = 150) stipulated a minimum number of standards between deviants and 56% (n = 104) stipulated at least two standards must separate deviants. There was no effect of deviant feature on the number of standards separating deviants. Both deviant probability and minimum number of standards emerged as significant predictors in regression analysis of latency data, with later peak latencies associated with greater number of standards separating deviants and less frequent deviants.

### 3.6. Isolating and manipulating deviant features, equating physicality of stimuli, and adaptation

**Table 1** shows 59% compared physically identical stimuli (n = 141) to ensure that differences in processing did not reflect differences in the physical properties of a stimulus. Of the available methods used to parse differences in physical properties of stimulus in **Table 1**, only the equiprobable and cascadic control accounts for differences in the ERPs due to adaptation. Only 23% (n = 54) included a control for adaptation (38% of those that used a physical control). Regression analysis shows that whether an adaptation control is used is the strongest predictor of mean amplitude, with larger amplitudes where a control was absent than when a control for adaptation was present.

### 3.7. Attention and fixation

Of all **Table 1** entries, 79% (n = 189) directed attention away from the stimulus of interest. It appears that directing attention away from the stimulus of interest is common practice in feature-deviance vMMN research. Of all **Table 1** entries, 66% (n = 158) used a fixation target, dot, square, cross, or tracking task and 60% (n = 142) directed attention away from the stimulus of interest and while using fixation stimulus. Attention emerged as a significant predictor of mean amplitude and peak latency, with larger and later vMMNs produced where attention was on vs. away from the relevant stimulus.

### 3.8. Reference and filter frequency

Of the 234 entries with reference details, 54% (n = 126) used a nose-tip or non-cephalic reference, 10% (n = 23) used a unipolar reference, 20% (n = 46) used the average, and 17% (n = 39) used linked or averaged earlobes/ mastoids/electrodes, illustrating a clear preference for the nose-tip/non-cephalic reference in vMMN research. The general approach to selecting a low-pass (LP) filter frequency in vMMN research is relatively consistent. Of those who reported a LP filter (n = 231), 48% (n = 112) used a 30 Hz cut-off. High-pass (HP) filter cut-off frequencies are more variable, ranging from 0.03–1.5 Hz.

### 3.9. Visual MMN region of interest (ROI) or electrode

Of the 233 entries with ROI or electrode details, 99% (n = 228) reported their results from parietal (P), occipital (O), and/or occipito-temporal ROIs or electrodes. Of those that specified a left or right ROI or electrode (n = 82), 78% (n = 64) indicated a right hemisphere ROI or electrode—22% (n = 18) reported a left hemisphere ROI or electrode.

### 3.10. Magnitude of deviance

#### 3.10.1. Orientation

Regression analysis including those significant predictors of mean amplitude as well as standardized magnitude of deviance saw magnitude of deviance replace SOI duration as a significant predictor of mean amplitude. The regression equation which best explained the variance, *R*^2^ = .297, *F*(3,73) = 9.851, *p* < .001, contained:

- whether a control for adaptation was used (ß = .214, *p* = .080), with larger amplitudes where a control was absent,
- whether attention was towards vs. away from the SOI (ß = −.304, *p* = .005), with larger amplitudes where attention was on the SOI, and
- magnitude of deviance (ß = −.330, *p* = .009), with larger amplitudes associated with larger magnitudes of deviance.

Including all 10 predictors (not deviant feature), the regression equation that best explained the variance in amplitude, *R*^2^ = .333, *F*(4,72) = 8.273, *p* < .001, contained:

- whether a control for adaptation was used (ß = .310, *p* = .011), with larger amplitudes where a control was absent,
- ISI (ß = .297, *p* = .006), with larger amplitudes associated with shorter ISIs,
- magnitude of deviance (ß = −.291, *p* = .016), with larger amplitudes associated with larger magnitudes of deviance, and

whether there was a fixation task or not (ß = .249, p = .019), with larger amplitudes where there was no fixation.

The variation explained by (and the trend in) magnitude of deviance was significant (and in the same direction) in both results for orientation entries. Regression analyses did not provide support for an effect of magnitude of deviance on peak latency for orientation deviants.

#### 3.10.2. Spatial frequency

No regression analyses produced a regression equation in which magnitude of deviance was a significant predictor of either mean amplitude or peak latency.

## 4. Discussion

This review comprises a tabular summary of the vMMN feature deviance research. Meta-analyses suggest that various parameters of experimental design can have (intended or unintended) effects on the resulting vMMN.

Of the 10 predictors explored overall, whether a control for adaptation was used, whether attention was towards vs. away from the relevant stimulus, and relevant stimulus duration emerged as significant predictors of vMMN mean amplitude while whether attention was towards vs. away from the relevant stimulus, ISI, deviant probability, and the number of standards separating deviants emerged as significant predictors of vMMN peak latency.

Magnitude of deviance also emerged as a significant predictor of mean amplitude for orientation entries—the most investigated feature of visual input in vMMN research. And, when including all 10 predictors in a regression analysis of the data for orientation entries, fixation (towards vs. away from the relevant stimulus) replaced attention as a significant predictor of mean amplitude.

In the discussion that follows, I consider parameters that emerged as a significant determinant of either mean amplitude or peak latency as well as parameters that did not but have been reported for influencing the vMMN. All results are exploratory and should be interpreted in the context of studies that examine the effect of varying any of the following parameters in the same participants given the limitations associated with revealing effects across participants as opposed to within the same participants.

### 4.1. Deviant feature

Relatively few have examined various deviants within the same participants [69, 73, 79, 142, 148]. In one study, Qian et al. [79] manipulated five features of visual input. They reported the largest vMMN for orientation deviants compared to colour, duration, shape, and size deviants. Sulykos and Czigler [69] also reported a larger vMMN for orientation deviants than for spatial-frequency deviants. However, the current results suggest that orientation deviants (−0.75±0.94 μV) produce smaller mean amplitudes than spatial frequency deviants (−1.69±1.57 μV). Location deviants (−1.69±0.99 μV) produced larger amplitudes than colour (−0.84±0.94 μV) and shape/size (−0.89±0.83 μV). This is in line with Grimm et al. [142], however, regression analysis did not provide any evidence that the deviant feature predicted vMMN mean amplitude (or peak latency). One possibility is that the remaining predictors primarily influence the amplitude differences, and once this is accounted for, the variability between feature deviants may be less than expected.

Although the current review and meta-analysis goes some way toward addressing this possibility by comparing results across deviant features, continued comparisons of deviant features in the same participants seems prudent for delineating whether different deviants evoke different sized vMMNs as in Grimm et al. [142] and for confirming the reasons for this, such as perceived difference, as in Takács et al. [72].

### 4.2. ISI and stimulus duration

It is generally accepted that shorter ISIs produce larger vMMNs than longer ISIs [55, 62, 150]. Illustrating this effect within the same participants, Fu et al. [62] found that a 100 ms ISI evoked a larger vMMN than a 400 ms ISI. Astikainen et al. [55] proposed that the short duration of the sensory memory trace—less than 1000 ms according to behavioural research [198–200]—was responsible for the absence of a vMMN in their 1000 ms ISI condition [55]. This view suggests that the monotonic relationship between ISI and vMMN is explained by memory trace decay.

Another possibility is that ISI duration determines confidence in the encoded regularity and, by corollary, regularity-based predictions. Daikhin and Ahissar [201] found that jittering the frequency values of standard tones—within a 0.5% range of 1000 Hz—yielded smaller MMNs than if all standards had the same frequency (i.e., 1000 Hz). They argued that confidence in the predictive model was lower in the jittered condition than in the non-jittered condition. Therefore, the strength of prediction determines, at least to some extent, the size of prediction error [4]. Perhaps ISI similarly determines confidence in the predictive model. Consider that if confidence is high because ISI is short, then perhaps prediction error is larger, yielding larger vMMNs, than when ISI is long. The current regression analyses indicate that shorter ISIs are associated with shorter vMMN peak latencies for deviants overall and that shorter ISIs are associated with larger vMMN amplitudes for orientation deviants.

This may also explain the observed association between stimulus duration and vMMN amplitudes in the current review, where longer stimulus durations enhance confidence in the predictive model, thereby generating larger vMMNs. However, as noted elsewhere [34], longer stimulus durations can pose challenges in EEG research because the onset of a stimulus can trigger eye movements toward it. According to Westheimer [202], saccades can begin 120 ms after stimulus onset and reach peak acceleration at 160 ms, falling within the vMMN time-window. Therefore, a shorter stimulus duration (e.g., ≤100 ms) may be better for avoiding confounds associated with eye movement and stimulus durations as short as 14 ms [140] and 17 ms [203] have been used to successfully evoke a vMMN for feature deviants.

Although others have manipulated stimulus duration as the deviant feature, there are no known studies that have manipulated stimulus duration to delineate its effect on the vMMN to an unrelated (non-duration) feature deviant. The current regression analysis indicates that longer stimulus presentations predict larger vMMNs, providing compelling rationale for further investigation into this phenomenon. The association not only highlights the importance of stimulus duration in understanding vMMN amplitudes but also underscores the potential for refining predictive models in EEG research.

### 4.3. Oddball deviant probability and minimum number of standards separating deviants

A deviant must be infrequent to constitute an irregularity. Standards establish regularity, making it important to separate deviants by at least two standards; otherwise, the deviant may not constitute an irregularity. Consider, however, that measures of deviance reflect increased deviant processing as well as reduced standard stimulus processing and that repetition suppression tends to increase with the number of standards preceding the deviant [106]. As a result, greater repetition suppression can create the appearance of a stronger response to deviance [96, 106], emphasizing the need to consider the number of standards that separate deviants.

Most studies in the current review reported a deviant probability of 20% or less. This may relate to the well-established association between higher deviant probabilities and smaller MMNs [204–207]. In the visual domain, Stefanics et al. [208] found that deviants with a lower (10%) probability evoked larger vMMNs than deviants with a higher (30%) probability. The authors also observed that conditions with 30% deviants produced a significant difference at 134–160 ms (earlier) whereas conditions with 10% deviants produced a significant difference at 232–254 ms (later). The current results further demonstrate that a higher deviant probability predicts earlier vMMN peak latencies, supporting the findings of Stefanics et al. [208]. Relatedly, later peak latencies were associated with a greater number of standards between deviants.

One explanation regards greater decay in the memory trace of the standard (e.g., [206, 207]). That is, as the memory trace of the standard stimulus fades over time, the brain’s ability to detect deviations from that standard diminishes. In conditions with higher percentages of deviants (like 30%), the brain may rely more on immediate comparisons rather than on a decaying memory of the standard. Alternatively, reasoning reflects enhanced memory trace for the deviant [204, 205].

Alternatively, the number of standards between deviants might influence the vMMN by influencing the ratio of successful: unsuccessful vMMN trials. If only some deviant trials generate a vMMN (due to the presence or absence of two or more standards preceding the deviant on some trials but not others), the resulting vMMN amplitude will be smaller compared to situations where all deviant trials elicit the vMMN, due to the averaging process. Horváth et al. [209] discussed this phenomenon in the context of magnitudes of deviance near the discrimination threshold. They proposed that, as the audible discrimination of irregular tones approaches the discrimination threshold, the ratio of trials that fail to evoke the MMN increases, while the ratio of deviant trials that evoke the MMN remains constant when the deviant is easily distinguishable. Consequently, averaging the trials creates the appearance of a magnitude of deviance effect limited to differences near the discriminable threshold. Similarly, if some trials evoke the vMMN while others do not, the overall vMMN amplitude will be smaller compared to situations where all deviant trials consistently elicit the vMMN.

Another possibility is that the neural correlate of a violation will be more pronounced when the model of regularity is more precise [210] and the model is more precise when there are more standards separating deviants. Several factors can influence model precision, including the stability or volatility of the environment [211–214] which is indeed determined by the similarity among standards. Still, the reason for the impact of volatility remains unclear. However, one possibility is that repetition suppression is greater in less volatile sequences.

Ultimately, there are various mechanisms by which the number of standards separating deviants may affect the vMMN, making it important to consider. Given the strong theoretical basis for ensuring a minimum number of standards separate deviants together with findings that repetition suppression increases as the number of standards preceding the deviant increases, affecting vMMN size, a minimum number of two standards is recommended.

### 4.4. Isolating and manipulating deviant features, equating physicality of stimuli, and adaptation

Whether a control for adaptation-related differences was absent or present emerged as a significant predictor of mean amplitude, illustrating the importance of controlling for adaptation-related-differences. Logically, the combined adaptation-related and deviance-related differences should yield a larger DRN than a genuine vMMN that is free from adaptation. Meanwhile, whether a physical control was present did not emerge as a significant predictor for latency or amplitude data. This may reflect the variability in true isolation of physical features of the visual stimulus due to use of comparatively complex visual stimuli.

Consider that a visual stimulus is composed of a unique combination of visual features, the presence of these features, along with their interaction, determines which neurons respond. Altering even a single feature changes the corresponding ERP. Despite this, many visual stimuli in vMMN research lack the simplicity of manipulation of a single property of visual input without influencing others. To illustrate, in a study by Mazza et al. [215], ERPs were compared for red triangle stimuli and green disc stimuli. Although their roles as deviants and standards were reversed, stimuli not only varied in colour, but also in all features that distinguish their shapes (e.g., contour orientation, stimulated retinal areas, spatial frequency). Therefore, any observed differences in their ERPs could arise from one, multiple, or all these variations.

Conversely, a key advantage of a Gabor patch lies in the separability of their stimulus features. Specifically, modifying a single aspect of a Gabor patch, such as its orientation, has no impact on other features like spatial frequency or luminance. Potentially, the true isolation of low-level features of the visual stimulus may explain why changes in Gabor patches yield much smaller vMMNs than similar changes in non-Gabor patch stimuli (*t*(225) = −4.167, *p* < .001). Another advantage of Gabor patch stimuli is that they are physiologically plausible visual stimuli due to their profile resembling that of a receptive field in the visual cortex’s simple cells [216–218]. They are also highly favoured among visual psychophysicists [216], facilitating the seamless translation of research findings between visual psychophysics and visual neuroscience.

Notably, larger deviant-minus-standard differences often peak earlier due to the N1 difference. Some researchers classify early (i.e., 150–200 ms) and late (200–250 ms) time-windows to distinguish between N1 adaptation (early) vs. deviance related differences (late) [79, 87, 91, 93, 99, 101] This may account for some of the early peak latencies reported where there is no control for adaptation (e.g., 130 ms in [69] and 134 ms in [72]). However, this approach cannot discount the possibility that adaptation-related differences are still present in the later time periods and may explain why there was no supporting evidence for an effect of adaptation on peak latency.

### 4.5. Attention, fixation, and ocular artifacts

There are several compelling theoretical and practical reasons for diverting attention away from the stimulus of interest in vMMN research. Firstly, when researchers initially explored the existence of a visual counterpart to the MMN, a crucial prerequisite was that it must occur without attention [21]. Focusing attention on the stimulus of interest hinders the ability to draw definitive conclusions about whether the vMMN genuinely mirrors the MMN [1, 2].

Secondly, directing attention away from the stimulus of interest is essential because attention can influence the vMMN [109, 180, 190]. For example, Kuldkepp et al. [180] found that directing attention away from the stimulus of interest is necessary to evoke both early and late subcomponents of the vMMN. However, there is also evidence that attention toward the stimulus of interest can enhance a vMMN response to a deviant that might not otherwise elicit a vMMN (e.g., [219]).

One potential explanation for the latter phenomenon is that the process of detecting deviance in vision differs from that in audition, such that some level of attention or an additional cognitive resource is required to detect changes in visual input. Another possibility is that attention could influence vMMN amplitudes by enhancing ERP components within the vMMN time window in which case, alterations in negativity might reflect shifts in attention rather than the direct detection of deviance (e.g., N2b, [220]).

While many of the reviewed studies used a central fixation stimulus, **Table 1** shows various other means have been employed for directing attention away from the stimulus of interest. For example, Sulykos and Czigler [69] designed the Spaceship task to control participants’ attention. Participants navigated a spaceship through a canyon—a rectangular object vertically and horizontally segmented giving the impression of depth and a horizon—while avoiding/catching colour-determined targets. Targets were other spaceships [83] or coloured doors [69]. Although this task ensures attention is effectively diverted away from the relevant stimulus, it does not ensure consistent eye gaze position because the eyes must move to recenter the object being tracked. In visual experiments, eye movements have the capacity to convert simple orientation changes into more complex visual alterations. Therefore, fixation tasks are ideal for ensuring that attention is diverted away from the stimulus of interest *and* ensuring that the visual system is exposed to the intended change in visual input, mitigating risk that other changes contribute to the observed ERP differences.

The fixation method was also one of the primary methods introduced for reducing ocular-artifacts [221]. This was typically used in combination with the rejection technique—discarding epochs containing voltage fluctuations in EOG or both EEG/EOG channels that are larger than a preset value is discarded [221]. Historically, the rejection method was most common [222]. Criticisms of the method include considerable loss of data and arbitrary threshold selection, with EOG artifacts below threshold still contributing to ERPs. In response to these criticisms, methods of correction rather than rejection, including regression analysis [221, 223], PCA [224], and ICA [225]. While the rejection technique is still widely used, the ICA approach has become increasingly popular in recent years (e.g., [93, 105, 133]). Of 68 studies since 2014 in **Table 1**, 43% (n = 29) used corrections to deal with ocular artifacts compared with the 56% that used rejection technique (n = 38). For a review of methods evolved to correct EEG artifacts see [226].

Despite the importance of ensuring that participants’ eyes remain open and fixated, the use of eye-tracking in the field is sparse, with only four **Table 1** studies having implemented some measure of gaze position [26, 34, 100, 178]. When astimulus does not occupy a uniform area of the visual field on every iteration, gaze position is more variable, and this has the potential to change a simple feature deviant into a more complex deviant. I previously reported greater gaze variability in an experimental condition in which a central grey bar appeared on a black background compared with an experimental condition in which a central Gabor patch appeared on a grey background [34]. Although the bar condition was a replication of Kimura et al. [66], we did not replicate the previously reported vMMN. One possible explanation is the additional emphasis on fixation in the replication condition via (i) use of eye-tracking, and (ii) additional intermixed conditions that required fixation for task completion. While gaze position was most variable in the replication condition, we believe it would have been even more variable without the added conditions and eye tracking. This variability can affect the perception of a simple feature deviant, making it more complex. Supporting this idea, regression analysis revealed that mean amplitudes were larger when fixation was not maintained than when it was.

In summary, the current results suggest that attention towards the stimulus of interest evokes larger and later vMMNs. Together with the strong theoretical and practical reasons for controlling fixation, the current review underscores the importance of considering attention and fixation in vMMN research.

### 4.6. Magnitude of deviance

Few have examined the relationship between magnitude of deviance and the vMMN, despite a potential to gain further insight into prediction error. Consider, for example, that if the sole purpose of the prediction error is to update the predictive model, then a monotonic relationship between the magnitude of deviance and the vMMN, in which larger magnitudes of deviance evoke larger vMMNs, is unnecessary per Horváth et al. [209]. Alternatively, if larger magnitudes of deviance produce larger prediction errors, then perhaps this is because larger vMMNs also predict later processes, such as the re-orienting of attention. Evidence of a monotonic relationship between amplitudes of the MMN and P3a—a known neural correlate of attention capture [157, 227]—supports the view that the size of the prediction error predicts attention switch [47, 228]. Another possibility is that the size of prediction error represents the extent of violation and this determines whether the model is abandoned versus updated [219].

Still, it remains unclear whether a monotonic relationship exists between magnitude of deviance and the vMMN. Several factors contribute to this ambiguity. Firstly, assertions about the connection between deviance magnitude and prediction error size largely rely on findings from MMN research, where there is a tendency for larger deviance magnitudes to elicit larger and earlier MMNs [16, 201, 228–235]). That said, certain experimental manipulations, such as attention allocation, have been found to affect the vMMN and MMN differently [26]. Given this, it is plausible that other experimental manipulations, like magnitude of deviance, have distinct effects on the vMMN and MMN.

Secondly, the existing body of research on the relationship between magnitude of deviance and the vMMN is both limited and marked by contradictions [57, 58, 61, 68, 72, 236]. For example, Czigler and Sulykos [68] found no discernible effect when using orientation deviants whereas Takács et al. [72] observed an effect on vMMN amplitude but not latency. Meanwhile, Maekawa et al. [57] reported an effect on vMMN latency but not amplitude. Maekawa et al. [57] manipulated spatial frequency via the number of vanes in a windmill pattern. Moreover, Clifford et al. [27] and Mo et al. [145] found that between-category colour deviants elicited larger vMMNs than within-category colour deviants, even though both deviants were equally distant from the standard on the colour spectrum. This suggests that the effect may be influenced more by an abstract property of the input (i.e., the category) rather than the specific feature itself (i.e., the colour). Supporting this interpretation, Thierry et al. [143] and Athanasopoulos et al. [237] demonstrated that learned linguistic associations—higher order top-down effects—influenced the perceived magnitude of deviance.

Meanwhile, Czigler et al. [58] found that small colour deviants (red vs. pink) did not evoke a vMMN whereas large colour deviants (red vs. green) did. Although this could represent a magnitude of deviance effect, it might also imply the need for a higher deviance threshold for colour deviants. The concept of a threshold has been considered elsewhere [235] and is supported by a study in which Takács et al. [72] increased their orientation difference from 50° to 90° after finding that the 90° difference more consistently evoked the vMMN than the 50° deviance. Following their first experiment, the authors conducted a psychophysical assessment on the threshold for orientation change detection with stimuli similar to those used the oddball paradigm. Taken together, the authors concluded that unlike the MMN in the auditory modality, in the visual modality, significantly larger differences are needed to evoke the vMMN. The threshold required for any feature deviant is not yet known and there is yet to be a study in which thresholds of deviance detection revealed by the vMMN are explored across deviant features.

The third and arguably the most challenging hurdle in deciphering the relationship between the magnitude of deviance and the (v)MMN lies in the fact that a significant portion of the research examining this relationship did not incorporate a control for adaptation. This poses a particular challenge because larger disparities between adapted and unadapted stimuli result in more substantial ERP differences, creating an appearance of a deviance magnitude effect (e.g., [201]). Recently, researchers who incorporated controls for adaptation while manipulating magnitude of deviance have not found the vMMN, let alone an effect of magnitude of deviance [106]. For these reasons, drawing conclusions about the association between magnitude of deviance and the vMMN based on the current literature remains challenging.

To help remedy this, I explored the magnitude of deviance effect in the tabulated orientation and spatial frequency entries separately. Regression analyses indicated larger amplitudes for larger magnitudes of deviance, indicative of a monotonic relationship, perhaps for the purpose of determining processes that follow such as attention or model updating. However, this finding relates to orientation only and there was no evidence of an effect of magnitude of deviance on vMMN amplitude or peak latency for spatial frequency deviants [57]. This may reflect the fewer spatial frequency entries. Alternatively, it may indicate that the effects of magnitude of deviance differ across deviants, as suggested by the aforementioned studies. Further research on the effect of magnitude of deviance under conditions where a genuine vMMN is achieved is needed to reach a conclusive understanding of the relationship between deviance magnitude and the vMMN.

### 4.7. EEG processing parameters

Although there are various processing parameters to consider, I focus on reference and filter frequency because both are known to have downwind effects on ERP component measures. Specifically, chosen reference can affect a component’s peak (e.g., amplitude), time of maximum negativity (e.g., peak latency), where it is largest (e.g., electrode or ROI), and even its polarity [238, 239]. A unipolar reference (e.g., the left or right earlobe or mastoid) can bias data toward one hemisphere by producing enlarged ERP amplitudes opposite the recording site [240]. In one **Table 1** study, Tales et al. [122] described a possible disadvantage in using references like Fz in that this is sometimes accompanied by frontal positivity in both the visual and auditory modalities and this would appear as additional negativity, thereby exaggerating posterior components. Re-referencing the data to the average of left and right reference electrodes (e.g., earlobes or mastoids) can help to avoid this [241]. This has been called linked-earlobes or linked-mastoids. However, linked-earlobes or linked-mastoids can also mean forcing the two sites to have the same voltage and this process does not alleviate the problem. The version of re-referencing is not always clear [240]. Although vision researchers considered the nose-tip electrically neutral and optimal in EEG research [240] re-referencing to the average is now considered optimal, especially for a high-density recording montage (i.e., at least 32 electrodes) [239–242].

Temporal filtering is a process in which one attenuates signals (e.g., electrical noise or activity) oscillating at a given rate. Frequency cut-off values in Hertz (Hz) determine the range of frequencies to preserve. For example, power mains in Australia produce regularly oscillating electrical activity at approximately 50 times per second (50 Hz). A low-pass filter cut-off value of below 50 Hz (e.g., 40 Hz) will attenuate the effect of all signals at frequencies above this value. Ultimately, the goal of filtering is to increase the signal-to-noise ratio (SNR) in the electrophysiological data and is therefore common practice in EEG research [243]. Cut-off frequencies that are >0.1 Hz (e.g., 1 Hz) can produce artifactual early negativities as early as 100 ms after onset [243–246]. This could be problematic where DRN or vMMN calculations include all ERP values between before 150 ms (e.g., [26, 27, 80, 86–88, 92, 102, 107, 108, 115, 120, 128, 135, 140, 174, 178, 182, 188, 193]).

### 4.8. Replicability of vMMN for feature deviants and its implications for meta-analysis

The current meta-analysis aims to uncover overarching trends and reveal patterns across studies. However, it also illuminates inconsistent effects and contextual factors underlying varying results. For example, Kimura et al. [66] reported a vMMN more than twice the size of the vMMN reported by Astikainen et al. [55] (−1.60 μV vs. −0.69 μV). Both studies used single grey bar stimuli on a black background, 36° orientation deviants, at least two standards between deviants, 100 ms stimulus duration, 400 ms ISI, and the equiprobable control. Similarly, despite using similar windmill-like patterns, Maekawa et al. [57] reported a vMMN at 185 ms, whereas File et al. [86] reported a vMMN at least 70 ms later. This may be related to deviance detection, such as the magnitude of deviance (i.e., the difference in the number of windmill-panes in File et al. [86] was six compared to 18 in Maekawa et al. [57]), or differences in processes outside of deviance detection, such as attention or adaptation (i.e., File et al. [86] used the equiprobable control whereas Maekawa et al. [57] compared identical stimuli as deviants and standards—reverse roles condition).

Moreover, there are an increasing number of studies emerging that illustrate the absence of a vMMN to changes in low-level features of visual input, like contrast, orientation, and spatial frequency (red values in **Table 1**). These are categorically low-level features because they are integral dimensions for describing the visual appearance of images and are processed in the visual cortex (V1) or earlier [34]. While the absence of vMMN is easier to reconcile in studies exploring rarely tested features, such as contrast, it poses a more challenging issue for frequently tested features like orientation and spatial frequency. Potentially, the stimulus used is implicated. For example, Sulykos et al. [71] did not observe the vMMN when using Gabor patches on a black background. The authors attributed this to a failure in reactivating the memory trace of a previous standard. However, others using centrally presented Gabor patches on a grey background also reported no vMMN for orientation changes of 36° [34] or 60° [106]. File et al. [86] also did not observe orientation vMMN when using peripherally presented line textures. Interestingly, however, in the same study, when using comparatively more complex stimuli than either Gabors or line textures (i.e., windmill patterns), File et al. [86] found that increases (not decreases) in spatial frequency (i.e., more windmill panes) evoked the vMMN whereas decreases in spatial frequency (i.e., fewer windmill panes) did not. They proposed that the former represented a more complex change requiring prediction error whereas the latter did not. However, additional changes in stimuli, such as added orientation information for increases but not decreases in spatial frequency, may contribute to the observed effects. This raises uncertainty about the capability of low-level feature such deviants to evoke the vMMN, let alone reveal the true effects of design parameters. If truly isolated changes to low-level features fail to elicit the vMMN, investigating how experimental design parameters impact the vMMN may require testing with comparatively more complex stimuli, such as windmill panes. The paradox arises, as controls for fixation, adaptation, and physicality become even more critical when using complex stimuli, they are less frequently implemented. The recommendations for parameters outlined in this review may potentially aid in designing paradigms to delineate these effects with complex stimuli within participants.

### 4.9. Limitations

Although the current review has many strengths, there are limitations to consider. For one, although there are many experimental parameters/variables, the number of variables analysed was limited to those that were comparable across studies while ensuring at least 10 distinct studies per predictor [247]. For example, although useful for showing the different sizes of stimuli used in vMMN research, stimulus size was not entered into regression analyses given that the precise area of the monitor occupied by the stimulus is not so easily quantified by any stimulus that is not simply a square, rectangle, or circle.

Additionally, despite the importance of adequate sample sizes, the studies reviewed had an average of only 17 participants, with some conditions including as few as six. **Table 1** shows that small, potentially underpowered samples remain common, posing risks to conclusions across much of the neuroscience literature. Small samples are associated with low replication rates, exaggerated effect estimates when findings are statistically significant, and poor predictive power [248]. These may contribute to the contradictory results observed in some entries in **Table 1**, underscoring the need for larger samples in the field.

Standardization of variables is another consideration. The minimum number of standards separating deviants (as integers) was coded as 0 for entries in which a minimum number of standards was not specified. Consequently, if this is not accurate (e.g., the authors did indeed ensure that at least 1 or more standard separated all deviants), the result may not be a true reflection of the relationship. Lastly, due to a sparse number of reported effect sizes in this area of work (details in supplementary materials **S1 Text**), the traditional means of illustrating bias which rely on effect sizes (or the means of calculating them via measures of variance) are difficult. Instead, a qualitative approach is used to summarize instances where values or pieces of information were estimated so as to not discount the entry entirely. Although the qualitative approach was recently used by Liu et al. [249], it does not facilitate an easily quantifiable assessment of bias in the field. To promote progress in the field, it would be beneficial to report effect sizes across all vMMN conditions in future studies.

## 5. Summary

I examined the existing literature on feature deviants to reveal the impact of experimental design parameters on the only known neural correlate of prediction error in the visual system—the vMMN. I explored the implications associated with these parameters either having or lacking an effect within the framework of predictive coding theory and reflected on the parameters worth considering, emphasizing practical and theoretical basis for certain parameter choices. I highlight existing gaps in knowledge concerning the effects of many design parameters, possibly due to inconsistencies in the literature and challenges in replicating the vMMN response to unanticipated changes in low-level features of visual input. As is often the case, the results encourage more questions than answers. However, this review may help guide those intending to pursue such critical questions, including does the vMMN differ for different feature deviants or does magnitude of deviance affect the vMMN similarly for all feature deviants? By demonstrating that the vMMN is affected by various parameters of experimental design, this review emphasizes the importance of a well-controlled and considered experimental design, for which the current review can also assist.

## Supporting information

S1 Text Systematic review. (DOCX)

S2 Text Bias estimates. (DOCX)

S3 Text Assessing certainty level of body of evidence. Contains **S1 Table:** GRADE assessment of risk of bias, unexplained heterogeneity or inconsistency of results, indirectness of evidence, indirectness of evidence, imprecision of results, probability of publication bias.
(DOCX)

S1 Fig PRISMA flow diagram. Illustrates the database searches, abstract screenings, and full-text retrievals conducted in the systematic review.
(PDF)

S2 Fig Summary of risk of bias. Red: High risk * Indicates that the value/parameter/condition is absent from the study design; Yellow: Unclear risk * Indicates that the value was estimated rather than explicitly stated in the text; Green: Low risk * Indicates that these parameters were clearly stated in the studies.
(PDF)

S1 Data Systematic review records. Original 948 records returned with exclusion/inclusion metrics.
(PDF)
