# Correction: Predicting the unpredicted … brain response: A systematic review of the feature-related visual mismatch negativity (vMMN) and the experimental parameters that affect it

**DOI:** 10.1371/journal.pone.0350243

**Published:** 2026-05-27

**Authors:** The PLOS One Editors

The following section is added to the Acknowledgements of this article [1]: Robert P. O’Shea contributed to the methodology and conceptualization, as well as writing of the manuscript. Urte Roeber, Bradley N. Jack, and an anonymous member of the Research Team contributed to the methodology and conceptualization of the manuscript.

Additionally, data presentation and formatting inconsistencies identified in Table 1 may prevent replication of the data.

An updated version of Table 1, in the format of an Excel file, can be requested by contacting PLOS directly. Please note that the updated table has been adapted by the *PLOS One* Editors and has not been verified by the author.
