# Supplementary material for: Predicting the unpredicted … brain response: A systematic review of the feature-related visual mismatch negativity (vMMN) and the experimental parameters that affect it
Source: PLoS One. 2025 Feb 27;20(2):e0314415. doi: 10.1371/journal.pone.0314415 (PMC11867396; doi:10.1371/journal.pone.0314415)
Supplement: S1 Text — (DOCX) [file pone.0314415.s001.docx]

**Supplementary Materials**

S1. Systematic Review

**S1 Fig** is a PRISMA flow diagram illustrating database searches, abstract screening, and full text retrievals in the systematic review. It shows a total of 1355 records were identified by AGM in Scopus, Web of Science, and Google Scholar using the following Boolean search: (“mismatch negativity” OR vMMN) and (vision OR visual OR “visual feature” OR “deviant feature”) and (spatial frequency OR color OR colour OR orientation OR size OR shape OR phase OR luminance OR location OR "motion direction" OR motion OR contrast OR duration OR omission) on 3 April 2024. AGM previously identified 80 articles in a review of the literature. Removing duplicates, 948 records remained (these records are available in **S1 Data**).

Records were screened for original peer-reviewed articles written in English. The remaining 850 records were examined by AGM for exclusion criteria. Of these, 250 had visual ERP data. Of these, 243 had ERP data from at least one healthy adult sample.

A further 98 records were excluded because there was no vMMN data and/or the change in the visual stimulus was relatively complex such that higher-order processing would become necessary such as facial or emotional expressions, associative relationships (e.g., sequential regularities or pairings), and lexical stimuli. AGM performed record inspection and inclusion selection.

**S1 Fig. PRISMA flow diagram.** Shows database searches, abstract screening, and full text retrievals in the systematic review.
