# Supplementary material for: Predicting the unpredicted … brain response: A systematic review of the feature-related visual mismatch negativity (vMMN) and the experimental parameters that affect it
Source: PLoS One. 2025 Feb 27;20(2):e0314415. doi: 10.1371/journal.pone.0314415 (PMC11867396; doi:10.1371/journal.pone.0314415)
Supplement: S2 Text — (DOCX) [file pone.0314415.s002.docx]

**Supplementary Materials**

S2. Bias estimates

Quality and bias assessment of included studies was conducted via the Review Manager (RevMan version 5.3) software for Cochrane reviews. Given how few studies reported effect sizes and/or measures of variance, funnel plots are not shown. This tendency to eschew effect sizes is less common in meta-analyses comparing effect sizes following intervention (e.g., [[1]](https://paperpile.com/c/KEY471/tx77)). Instead, as in the meta-analysis by Liu et al. [[1]](https://paperpile.com/c/KEY471/tx77), **S2 Fig** illustrates the degree of potential bias associated with each published study given the absence of one or more pieces of information and/or the absence of a physical and adaptation control. Green is no–low risk, orange is unclear risk, red represents high risk.

**S2 Fig. Summary of risk of bias.** Red: High risk * Because the value/parameter/condition is not available/present in the study design; Yellow: Unclear risk * Because the value was estimated rather than stated in the text; Green: Low risk * Because the studies stated these parameters.

References

1. [Liu Z, Tao X, Chen Y, Fan Z, Li Y. Bed rest versus early ambulation with standard anticoagulation in the management of deep vein thrombosis: a meta-analysis. PLoS One. 2015;10: e0121388.](http://paperpile.com/b/KEY471/tx77)
