# Supplementary material for: Predicting the unpredicted … brain response: A systematic review of the feature-related visual mismatch negativity (vMMN) and the experimental parameters that affect it
Source: PLoS One. 2025 Feb 27;20(2):e0314415. doi: 10.1371/journal.pone.0314415 (PMC11867396; doi:10.1371/journal.pone.0314415)
Supplement: S3 Text — Contains S1 Table: GRADE assessment of risk of bias, unexplained heterogeneity or inconsistency of results, indirectness of evidence, indirectness of evidence, imprecision of results, probability of publication bias. (DOCX) [file pone.0314415.s003.docx]

**Supplementary Materials**

S3. Assessing certainty level of body of evidence

**S1 Table** shows the GRADE assessment of (1) Risk of bias, (2) Unexplained heterogeneity or inconsistency of results, (3) Indirectness of evidence, (4) Imprecision of results, (5) Probability of publication bias.

**S1 Table.** **GRADE assessment of risk of bias, unexplained heterogeneity or inconsistency of results, indirectness of evidence, indirectness of evidence, imprecision of results, probability of publication bias.**

| Domains for assessing certainty of evidence by outcome | Assessment | GRADE assessment of risk of bias or study limitations of study limitations |
| --- | --- | --- |
| (1) Risk of bias | The proportion of information from results at high risk of bias is sufficient to affect the interpretation of results. | High Risk of Bias  Downgrade certainty |
| (2) Inconsistency of results | One of the motivators of the current review is to delineate why large inconsistencies in the literature exist. | Moderate inconsistency Downgrade certainty |
| (3) Indirectness of evidence | While some of the reported studies explored elements examined in the current study (e.g., inter-stimulus-interval), none considered all elements included in the linear regression analysis. For this reason, all findings are interpreted second to those who examined those elements directly. | Moderate indirectness Downgrade certainty |
| (4) Imprecision of results | While the number of participants per study was relatively small (~17), the aggregated data across all 145 studies yielded 2499, allowing the current review to detect smaller effects than what is typically possible | Improved precision  Upgrade certainty |
| (5) High probability of publication bias | An increasing number of studies being published showing no/null effect suggests reduced publication bias. Noting however, those studies which report a condition in which there is no effect, a different condition is reported in which there is an effect. | Inclusion of null effects in analysis  Upgrade certainty |

References

1. [Liu Z, Tao X, Chen Y, Fan Z, Li Y. Bed rest versus early ambulation with standard anticoagulation in the management of deep vein thrombosis: a meta-analysis. PLoS One. 2015;10: e0121388.](http://paperpile.com/b/KEY471/tx77)
