# Supplementary material for: Predicting the unpredicted … brain response: A systematic review of the feature-related visual mismatch negativity (vMMN) and the experimental parameters that affect it
Source: PLoS One. 2025 Feb 27;20(2):e0314415. doi: 10.1371/journal.pone.0314415 (PMC11867396; doi:10.1371/journal.pone.0314415)
Supplement: S1 Fig — Illustrates the database searches, abstract screenings, and full-text retrievals conducted in the systematic review. (PDF) [file pone.0314415.s004.pdf]

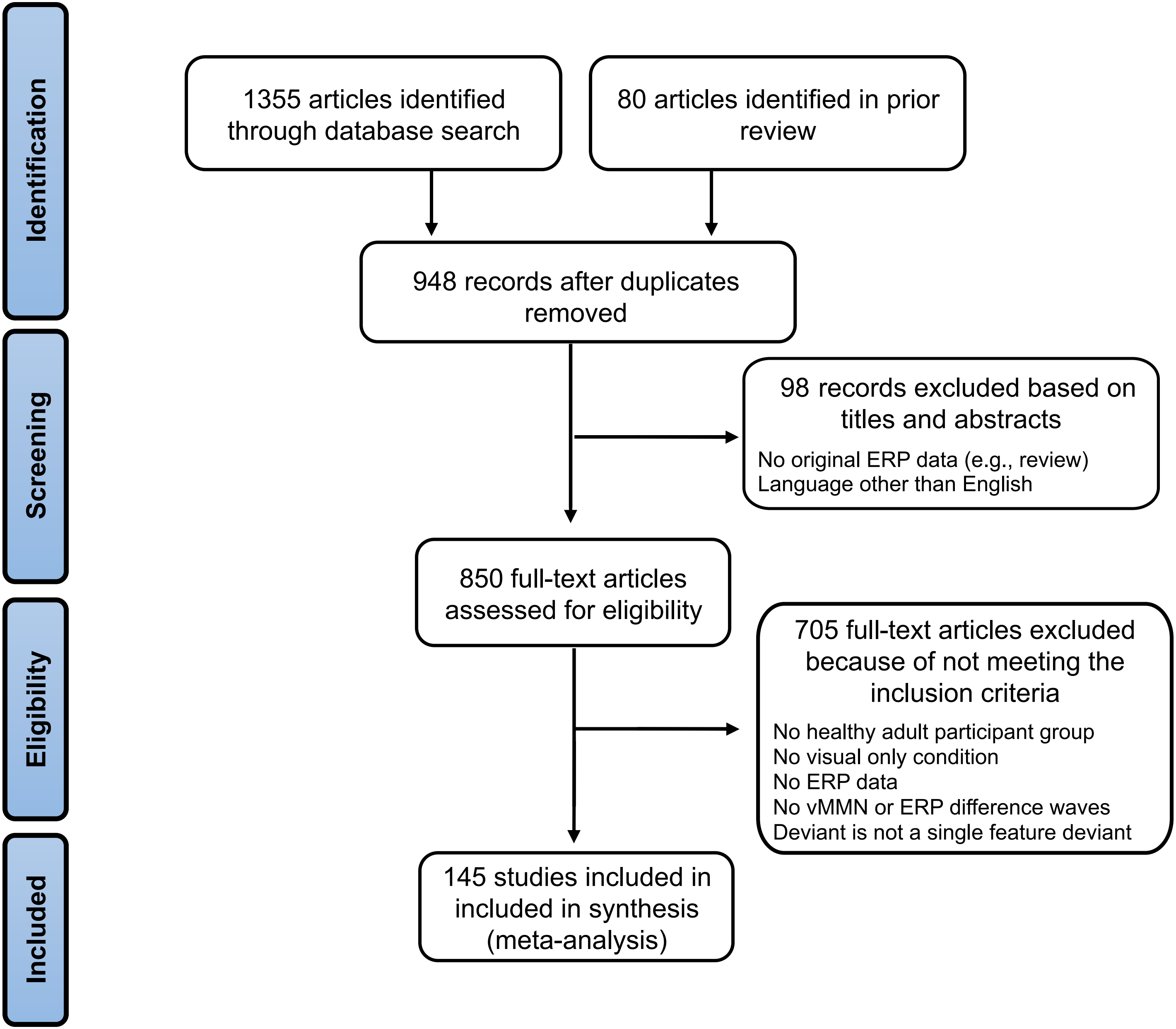
Identification
Screening
Eligibility
Included
1355 articles identified through database search
80 articles identified in prior review
948 records after duplicates removed
98 records excluded based on titles and abstracts
No original ERP data (e.g., review)
Language other than English
850 full-text articles assessed for eligibility
705 full-text articles excluded because of not meeting the inclusion criteria
No healthy adult participant group
No visual only condition
No ERP data
No vMMN or ERP difference waves
Deviant is not a single feature deviant
145 studies included in included in synthesis (meta-analysis)
