# Supplementary figures and images for: Predicting the unpredicted … brain response: A systematic review of the feature-related visual mismatch negativity (vMMN) and the experimental parameters that affect it
Source: PLoS One. 2025 Feb 27;20(2):e0314415. doi: 10.1371/journal.pone.0314415 (PMC11867396; doi:10.1371/journal.pone.0314415)

#### VI - Selective reporting (reporting bias: no effect size $1 \leq$ conditions)

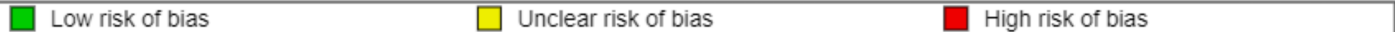[illegible]

Supplement: S2 Fig — Red: High risk * Indicates that the value/parameter/condition is absent from the study design; Yellow: Unclear risk * Indicates that the value was estimated rather than explicitly stated in the text; Green: Low risk * Indicates that these parameters were clearly stated in the studies. (PDF) [file pone.0314415.s005.pdf]
