# Supplementary material for: Predicting the unpredicted … brain response: A systematic review of the feature-related visual mismatch negativity (vMMN) and the experimental parameters that affect it
Source: PLoS One. 2025 Feb 27;20(2):e0314415. doi: 10.1371/journal.pone.0314415 (PMC11867396; doi:10.1371/journal.pone.0314415)
Supplement: S1 Data — Original 948 records returned with exclusion/inclusion metrics. (PDF) [file pone.0314415.s006.pdf]

	Study ID	English	Journal	SUMMARY	Visual only condition	ERP	SUMMARY	Human	Healthy adult	SUMMARY	vMMN data	Single feature	SUMMARY
1	Bitz_2009_Elektrophysiologisch	0	0	0	-	-	0	-	-	0	-	-	0
2	Plitzko_2006_P300 und Mismatch Ne	0	0	0	-	-	0	-	-	0	-	-	0
3	Souza_2019_Potencial evocado au	0	0	0	-	-	0	-	-	0	-	-	0
4	Holdefer_2015_Análise da latência	0	0	0	-	-	0	-	-	0	-	-	0
5	Eschrich_2006_Akustische EKP-Unter	0	0	0	-	-	0	-	-	0	-	-	0
6	Schuller_2018_Die Mismatch Negativ	0	0	0	-	-	0	-	-	0	-	-	0
7	Milotinský_2016_Vliv psilocybinu na	0	0	0	-	-	0	-	-	0	-	-	0
8	Shestopalova_2015_Topography of the Ev	0	0	0	-	-	0	-	-	0	-	-	0
9	An_2021_Cortical mapping of	1	0	0	-	-	0	-	-	0	-	-	0
10	Wu_2014_Delta and beta EEG o	1	0	0	-	-	0	-	-	0	-	-	0
11	Haigh_2017_Mismatch Negativity	1	0	0	-	-	0	-	-	0	-	-	0
12	Kelly_2019_Neural correlates of	1	0	0	-	-	0	-	-	0	-	-	0
13	Green_2009_Perception measureme	1	0	0	-	-	0	-	-	0	-	-	0
14	Mazer_2024_Autistic traits and	1	0	0	-	-	0	-	-	0	-	-	0
15	Hsiao_2009_Theta oscillation du	1	0	0	-	-	0	-	-	0	-	-	0
16	Hsiao_2010_Cortico-cortical pha	1	0	0	-	-	0	-	-	0	-	-	0
17	Butler_2012_Perceptual Measureme	1	0	0	-	-	0	-	-	0	-	-	0
18	Michie_2001_What has MMN reveale	1	0	0	-	-	0	-	-	0	-	-	0
19	Hughes_2001_Responses of human a	1	0	0	-	-	0	-	-	0	-	-	0
20	Javitt_2023_Cognitive Impairment	1	0	0	-	-	0	-	-	0	-	-	0
21	Kojima_2014_A stroke patient wit	1	0	0	-	-	0	-	-	0	-	-	0
22	Tome_2015_Mismatch negativity	1	0	0	-	-	0	-	-	0	-	-	0
23	Cong_2010_Evaluation and extra	1	0	0	-	-	0	-	-	0	-	-	0
24	File_2019_INVESTIGATING THE UN	1	0	0	-	-	0	-	-	0	-	-	0
25	O'Shea_2015_Refractoriness about	1	0	0	-	-	0	-	-	0	-	-	0
26	Czigler_2023_Opinion on the event	1	0	0	-	-	0	-	-	0	-	-	0
27	Mazer_2023_Systematic review an	1	0	0	-	-	0	-	-	0	-	-	0
28	Raggi_2022_Auditory mismatch ne	1	0	0	-	-	0	-	-	0	-	-	0
29	Fong_2020_Auditory mismatch ne	1	0	0	-	-	0	-	-	0	-	-	0
30	Jack_2014_Conscious and not-co	1	0	0	-	-	0	-	-	0	-	-	0
31	Sultson_2022_Corrigendum to "Hung	1	0	0	-	-	0	-	-	0	-	-	0
32	Hadid_2017_Visual mismatch nega	1	0	0	-	-	0	-	-	0	-	-	0
33	Tales_2009_Visual Mismatch nega	1	0	0	-	-	0	-	-	0	-	-	0
34	Näätänen_1988_Implications of ERP	1	0	0	-	-	0	-	-	0	-	-	0

35	Ritter_1995_The mismatch negativ	1	0	0	-	-	0	-	-	0	-	-	0
36	Kimura_2011_Visual mismatch nega	1	0	0	-	-	0	-	-	0	-	-	0
37	Kimura_2012_Visual mismatch nega	1	0	0	-	-	0	-	-	0	-	-	0
38	Onitsuka_2013_Review of neurophysi	1	0	0	-	-	0	-	-	0	-	-	0
39	Escera_2007_Role of mismatch neg	1	0	0	-	-	0	-	-	0	-	-	0
40	González_2017_Contributions of let	1	0	0	-	-	0	-	-	0	-	-	0
41	Ahmed_2005_A Study of Auditory	1	0	0	-	-	0	-	-	0	-	-	0
42	Escera_2007_The mismatch negativ	1	0	0	-	-	0	-	-	0	-	-	0
43	Chow_2023_Mismatch Negativity	1	0	0	-	-	0	-	-	0	-	-	0
44	Tsui_2000_Mismatch negativity	1	0	0	-	-	0	-	-	0	-	-	0
45	Czigler_2014_Visual mismatch nega	1	0	0	-	-	0	-	-	0	-	-	0
46	Javitt_2018_Mismatch negativity	1	0	0	-	-	0	-	-	0	-	-	0
47	Himberger_2018_Principles of Tempor	1	0	0	-	-	0	-	-	0	-	-	0
48	Maekawa_2012_Auditory and visual	1	0	0	-	-	0	-	-	0	-	-	0
49	Winkler_2012_Evidence from audito	1	0	0	-	-	0	-	-	0	-	-	0
50	Connor_1989_Attention, mismatch	1	0	0	-	-	0	-	-	0	-	-	0
51	Nguyen_2012_A Linguistic Relativ	1	0	0	-	-	0	-	-	0	-	-	0
52	Shtyrov_2007_Language in the mism	1	0	0	-	-	0	-	-	0	-	-	0
53	Berteau_2018_Modeling biophysical	1	0	0	-	-	0	-	-	0	-	-	0
54	Leung_2008_Examining the Pharma	1	0	0	-	-	0	-	-	0	-	-	0
55	Sulykos_2017_Visual mismatch nega	1	0	0	-	-	0	-	-	0	-	-	0
56	Czigler_2007_Visual mismatch nega	1	0	0	-	-	0	-	-	0	-	-	0
57	Isenstein_2023_Poster Session: Neur	1	0	0	-	-	0	-	-	0	-	-	0
58	Winkler_1993_Mismatch negativity:	1	0	0	-	-	0	-	-	0	-	-	0
59	Mangold_2021_The Stability of the	1	0	0	-	-	0	-	-	0	-	-	0
60	Alzaher_2020_Mismatch Negativity	1	0	0	-	-	0	-	-	0	-	-	0
61	Auvinen_2001_The detection of the	1	0	0	-	-	0	-	-	0	-	-	0
62	Schröger_2007_Mismatch negativity:	1	0	0	-	-	0	-	-	0	-	-	0
63	Näätänen_2019_Mismatch negativity:	1	0	0	-	-	0	-	-	0	-	-	0
64	Gené-Cos_1999_Possible roles for m	1	0	0	-	-	0	-	-	0	-	-	0
65	Näätänen_2012_The mismatch negativ	1	0	0	-	-	0	-	-	0	-	-	0
66	Näätänen_2009_The mismatch negativ	1	0	0	-	-	0	-	-	0	-	-	0
67	Näätänen_1995_The mismatch negativ	1	0	0	-	-	0	-	-	0	-	-	0
68	Garrido_2009_The mismatch negativ	1	0	0	-	-	0	-	-	0	-	-	0
69	Iso-Markku_2020_Twin studies on the	1	0	0	-	-	0	-	-	0	-	-	0
70	Jarkiewicz_2015_Can new paradigms br	1	0	0	-	-	0	-	-	0	-	-	0
71	Baldeweg_2007_ERP repetition effec	1	0	0	-	-	0	-	-	0	-	-	0

72	Kantrowitz_2012_Glutamatergic transm	1	0	0	-	-	0	-	-	0	-	-	0
73	Blacker_2021_Deficits in visual p	1	0	0	-	-	0	-	-	0	-	-	0
74	Schröger_2023_Markov chains as a p	1	0	0	-	-	0	-	-	0	-	-	0
75	Kalyakin_2010_Extraction of mismat	1	0	0	-	-	0	-	-	0	-	-	0
76	Schryver_2022_Mismatch Negativity	1	0	0	-	-	0	-	-	0	-	-	0
77	Mustonen_2007_Preattentive detecti	1	0	0	-	-	0	-	-	0	-	-	0
78	O'Reilly_2021_A critical review of	1	0	0	-	-	0	-	-	0	-	-	0
79	Paavilainen_2013_The mismatch-negativ	1	0	0	-	-	0	-	-	0	-	-	0
80	Kremláček_2016_Visual mismatch nega	1	0	0	-	-	0	-	-	0	-	-	0
81	Stefanics_2014_Visual mismatch nega	1	0	0	-	-	0	-	-	0	-	-	0
82	Kremlacek_2009_Magnocellular system	1	0	0	-	-	0	-	-	0	-	-	0
83	KREEGIPUU_2019_WHEN SUBJECTIVE IS T	1	0	0	-	-	0	-	-	0	-	-	0
84	Stefanics_2016_Mismatch negativity	1	0	0	-	-	0	-	-	0	-	-	0
85	Campbell_2006_The N1 hyprthesis of	1	0	0	-	-	0	-	-	0	-	-	0
86	Fotidzis_2020_Phonology, prosody,	1	0	0	-	-	0	-	-	0	-	-	0
87	Lecaignard_2023_Mismatch Negativity:	1	0	0	-	-	0	-	-	0	-	-	0
88	Pazo-Alvarez_2003_MMN in the visual mo	1	0	0	-	-	0	-	-	0	-	-	0
89	Astikainen_2022_Visual mismatch nega	1	0	0	-	-	0	-	-	0	-	-	0
90	André-Obadia_2018_Recommendations for	1	0	0	-	-	0	-	-	0	-	-	0
91	Beauchemin_2005_Statistical analysis	1	0	0	-	-	0	-	-	0	-	-	0
92	Millichamp_2018_A small step closer	1	0	0	-	-	0	-	-	0	-	-	0
93	Bissonnette_2022_Examining the Simple	1	0	0	-	-	0	-	-	0	-	-	0
94	Athanasopoulos_2009_The Whorfian mind: E	1	0	0	-	-	0	-	-	0	-	-	0
95	Matziorinis_2017_Towards a Non-Invasi	1	0	0	-	-	0	-	-	0	-	-	0
96	NONE,_2014_1st International Ai	1	0	0	-	-	0	-	-	0	-	-	0
97	Harnett_2020_ACNP 59th Annual Mee	1	0	0	-	-	0	-	-	0	-	-	0
98	Winkler_2007_Interpreting the mis	1	0	0	-	-	0	-	-	0	-	-	0
99	Hsu_2010_Memory-based mismatc	1	1	1	0	0	0	-	-	0	-	-	0
100	Van_2013_Alignment to natural	1	1	1	0	0	0	-	-	0	-	-	0
101	Shin_2009_Pre-Attentive Audito	1	1	1	0	0	0	-	-	0	-	-	0
102	Yabe_1998_Temporal window of i	1	1	1	0	0	0	-	-	0	-	-	0
103	Mill_2011_A Neurocomputational	1	1	1	0	0	0	-	-	0	-	-	0
104	Sauer_2023_Spectral and phase-c	1	1	1	0	0	0	-	-	0	-	-	0
105	Hsiao_2014_Temporo-Frontal Func	1	1	1	0	0	0	-	-	0	-	-	0
106	Lappe_2011_Cortical Plasticity	1	1	1	0	0	0	-	-	0	-	-	0
107	Araki_2020_A neural network mod	1	1	1	0	0	0	-	-	0	-	-	0
108	Kaiser_2001_Location changes enh	1	1	1	0	0	0	-	-	0	-	-	0

109	Oshima_2017_Alteration of Durati	1	1	1	0	0	0	-	-	0	-	-	0
110	Sauer_2017_Impairment in predic	1	1	1	0	0	0	-	-	0	-	-	0
111	Gaebler_2015_Auditory mismatch im	1	1	1	0	0	0	-	-	0	-	-	0
112	Lattner_2003_Dissociation of huma	1	1	1	0	0	0	-	-	0	-	-	0
113	Rosburg_2003_Left hemispheric dip	1	1	1	0	0	0	-	-	0	-	-	0
114	Sysoeva_2006_Pre-attentive repres	1	1	1	0	0	0	-	-	0	-	-	0
115	Takei_2009_Preattentive dysfunc	1	1	1	0	0	0	-	-	0	-	-	0
116	Inouchi_2003_Magnetic mismatch fi	1	1	1	0	0	0	-	-	0	-	-	0
117	Rosburg_2004_The dipole location	1	1	1	0	0	0	-	-	0	-	-	0
118	Møller_2019_Poorer auditory sens	1	1	1	0	0	0	-	-	0	-	-	0
119	Ulanov_2024_Regionally specific	1	1	1	0	0	0	-	-	0	-	-	0
120	Recasens_2017_Test-retest reliabil	1	1	1	0	0	0	-	-	0	-	-	0
121	Kujala_2004_Activation in the an	1	1	1	0	0	0	-	-	0	-	-	0
122	Yumoto_2005_Auditory imagery mis	1	1	1	0	0	0	-	-	0	-	-	0
123	Zakeri_2021_Using Mismatch Negat	1	1	1	0	0	0	-	-	0	-	-	0
124	Mikanmaa_2020_Neuromagnetic mismat	1	1	1	0	0	0	-	-	0	-	-	0
125	Hayakawa_2013_Anterior cingulate a	1	1	1	0	0	0	-	-	0	-	-	0
126	Dheerendra_2024_Intact mismatch nega	1	1	1	0	0	0	-	-	0	-	-	0
127	Dittmann-Balçar_2001_Dorsolateral prefron	1	1	1	0	0	0	-	-	0	-	-	0
128	López-Caballero_2023_Is source-resolved m	1	1	1	0	0	0	-	-	0	-	-	0
129	Ko_2012_Theta Oscillation Re	1	1	1	0	1	0	-	-	0	-	-	0
130	He_2021_Performance-dependen	1	1	1	0	1	0	-	-	0	-	-	0
131	He_2013_Effects of alcohol o	1	1	1	0	1	0	-	-	0	-	-	0
132	An_2021_Do Auditory Mismatch	1	1	1	0	1	0	-	-	0	-	-	0
133	Yu_2018_The Duration of Audi	1	1	1	0	1	0	-	-	0	-	-	0
134	Wei_2018_Written-Word Concret	1	1	1	0	1	0	-	-	0	-	-	0
135	Tse_2006_Preattentive timing	1	1	1	0	1	0	-	-	0	-	-	0
136	Lin_2014_A recently-discovere	1	1	1	0	1	0	-	-	0	-	-	0
137	Hsu_2023_The mismatch negativ	1	1	1	0	1	0	-	-	0	-	-	0
138	Liu_2018_One Way or Another:	1	1	1	0	1	0	-	-	0	-	-	0
139	Hsu_2011_TIME-FREQUENCY ANALY	1	1	1	0	1	0	-	-	0	-	-	0
140	Tse_2008_On the functional ro	1	1	1	0	1	0	-	-	0	-	-	0
141	Todd_2008_Deviant matters:: Du	1	1	1	0	1	0	-	-	0	-	-	0
142	Kane_1996_Event related potent	1	1	1	0	1	0	-	-	0	-	-	0
143	He_2020_Effects of visual at	1	1	1	0	1	0	-	-	0	-	-	0
144	Tada_2020_Translatability of S	1	1	1	0	1	0	-	-	0	-	-	0
145	Suda_2022_Prediction-Related F	1	1	1	0	1	0	-	-	0	-	-	0

146	KING_1995_ACOUSTIC FEATURES AN	1	1	1	0	1	0	-	-	0	-	-	0
147	Kask_2021_Perceptual Asymmetri	1	1	1	0	1	0	-	-	0	-	-	0
148	Fang_2021_Single dose testoste	1	1	1	0	1	0	-	-	0	-	-	0
149	Ells_2018_Alterations of compl	1	1	1	0	1	0	-	-	0	-	-	0
150	Fu_2016_A mismatch negativit	1	1	1	0	1	0	-	-	0	-	-	0
151	Yabe_2005_Transient gamma-band	1	1	1	0	1	0	-	-	0	-	-	0
152	Wang_2021_Test-retest reliabil	1	1	1	0	1	0	-	-	0	-	-	0
153	Wang_2019_Letter-sound integra	1	1	1	0	1	0	-	-	0	-	-	0
154	Valt_2023_Reduced magnetic mis	1	1	1	0	1	0	-	-	0	-	-	0
155	Todd_2001_Do loudness cues con	1	1	1	0	1	0	-	-	0	-	-	0
156	Suga_2016_Magnetoencephalograp	1	1	1	0	1	0	-	-	0	-	-	0
157	Choi_2013_Fronto-temporal inte	1	1	1	0	1	0	-	-	0	-	-	0
158	Hall_2009_Are auditory P300 an	1	1	1	0	1	0	-	-	0	-	-	0
159	Yu_2022_Distinct but integra	1	1	1	0	1	0	-	-	0	-	-	0
160	Tata_2005_Early phase of spati	1	1	1	0	1	0	-	-	0	-	-	0
161	Li_Effects of the cardiac cycle on auditory pro	1	1	1	0	1	0	-	-	0	-	-	0
162	Hall_2006_Genetic overlap betw	1	1	1	0	1	0	-	-	0	-	-	0
163	Hall_2006_Heritability and rel	1	1	1	0	1	0	-	-	0	-	-	0
164	Riel_2022_MMN and P3a Elicited	1	1	1	0	1	0	-	-	0	-	-	0
165	Todd_2024_Order effects in tas	1	1	1	0	1	0	-	-	0	-	-	0
166	Mu_2016_Pre-attentive dysfun	1	1	1	0	1	0	-	-	0	-	-	0
167	Yang_2016_Unsuccessful letter-	1	1	1	0	1	0	-	-	0	-	-	0
168	Tunc_2019_Predictive coding an	1	1	1	0	1	0	-	-	0	-	-	0
169	Riel_2023_Reduced duration mis	1	1	1	0	1	0	-	-	0	-	-	0
170	Yabe_1997_Temporal window of i	1	1	1	0	1	0	-	-	0	-	-	0
171	Alho_1992_Selective Attention	1	1	1	0	1	0	-	-	0	-	-	0
172	Thye_2018_The impact of atypic	1	1	1	0	1	0	-	-	0	-	-	0
173	Kane_1996_Event-related potent	1	1	1	0	1	0	-	-	0	-	-	0
174	Tunc_2019_Predictive coding an	1	1	1	0	1	0	-	-	0	-	-	0
175	Sugi_2007_Feature extraction f	1	1	1	0	1	0	-	-	0	-	-	0
176	Frey_2017_Changes in room acou	1	1	1	0	1	0	-	-	0	-	-	0
177	Grimm_2006_Mechanisms for detec	1	1	1	0	1	0	-	-	0	-	-	0
178	Colin_2002_Mismatch negativity	1	1	1	0	1	0	-	-	0	-	-	0
179	Dyson_2005_Effects of visual at	1	1	1	0	1	0	-	-	0	-	-	0
180	Gomes_2013_Automatic Processing	1	1	1	0	1	0	-	-	0	-	-	0
181	Fan_2023_Phonemic mismatch ne	1	1	1	0	1	0	-	-	0	-	-	0
182	Lovio_2009_Auditory discriminat	1	1	1	0	1	0	-	-	0	-	-	0

183	Glass_2008_Development of audit	1	1	1	0	1	0	-	-	0	-	-	0
184	Datta_2010_Electrophysiological	1	1	1	0	1	0	-	-	0	-	-	0
185	T_2021_Mismatch negativity	1	1	1	0	1	0	-	-	0	-	-	0
186	Löw_2019_Multifeature mismatc	1	1	1	0	1	0	-	-	0	-	-	0
187	Desai_2021_Phase 1 randomized s	1	1	1	0	1	0	-	-	0	-	-	0
188	Yao_2011_Preattentive process	1	1	1	0	1	0	-	-	0	-	-	0
189	Xiong_2019_Differential of Freq	1	1	1	0	1	0	-	-	0	-	-	0
190	Wiens_2017_Data on the auditory	1	1	1	0	1	0	-	-	0	-	-	0
191	Uhlén_2017_Using a multi-featur	1	1	1	0	1	0	-	-	0	-	-	0
192	Rinne_2005_Superior temporal an	1	1	1	0	1	0	-	-	0	-	-	0
193	Irwin_2018_Electrophysiological	1	1	1	0	1	0	-	-	0	-	-	0
194	Innes_2022_Modeling Distraction	1	1	1	0	1	0	-	-	0	-	-	0
195	Haden_2009_Timbre-independent e	1	1	1	0	1	0	-	-	0	-	-	0
196	Gao_2012_Integration of conso	1	1	1	0	1	0	-	-	0	-	-	0
197	Chien_2019_A generic deviance d	1	1	1	0	1	0	-	-	0	-	-	0
198	Cheng_2013_Effects of physiolog	1	1	1	0	1	0	-	-	0	-	-	0
199	CATTS_1995_BRAIN POTENTIAL EVID	1	1	1	0	1	0	-	-	0	-	-	0
200	Brima_2019_Auditory sensory mem	1	1	1	0	1	0	-	-	0	-	-	0
201	Brace_2021_The Brain Tracks Mul	1	1	1	0	1	0	-	-	0	-	-	0
202	Berti_2013_The role of auditory	1	1	1	0	1	0	-	-	0	-	-	0
203	Zou_2023_Full-form vs. combin	1	1	1	0	1	0	-	-	0	-	-	0
204	Yeark_2023_The impact of spatia	1	1	1	0	1	0	-	-	0	-	-	0
205	Jones_2016_ERPs Reveal the Time	1	1	1	0	1	0	-	-	0	-	-	0
206	Broyd_2016_Schizotypy and audit	1	1	1	0	1	0	-	-	0	-	-	0
207	Brima_2024_Assessing the integr	1	1	1	0	1	0	-	-	0	-	-	0
208	PIHKO_1995_BRAIN REACTS TO OCCA	1	1	1	0	1	0	-	-	0	-	-	0
209	Grimm_2004_Differential process	1	1	1	0	1	0	-	-	0	-	-	0
210	Barry_2008_Duration of auditory	1	1	1	0	1	0	-	-	0	-	-	0
211	Peter_2010_Effect of deviance d	1	1	1	0	1	0	-	-	0	-	-	0
212	Gomes_1999_Electrophysiological	1	1	1	0	1	0	-	-	0	-	-	0
213	Nowak_2016_Electrophysiological	1	1	1	0	1	0	-	-	0	-	-	0
214	Sun_2020_Hyper-activated brai	1	1	1	0	1	0	-	-	0	-	-	0
215	Rahne_2013_Influence of auditor	1	1	1	0	1	0	-	-	0	-	-	0
216	Lai_2011_Involuntary Processi	1	1	1	0	1	0	-	-	0	-	-	0
217	Raz_2001_Is pre-attentive pro	1	1	1	0	1	0	-	-	0	-	-	0
218	Fan_2018_Modulation of audito	1	1	1	0	1	0	-	-	0	-	-	0
219	Vuust_2011_New fast mismatch ne	1	1	1	0	1	0	-	-	0	-	-	0

220	Grimm_2005_Pre-attentive and at	1	1	1	0	1	0	-	-	0	-	-	0
221	Wolff_2008_The neural mechanism	1	1	1	0	1	0	-	-	0	-	-	0
222	Grimm_2012_Ultrafast tracking o	1	1	1	0	1	0	-	-	0	-	-	0
223	Rahne_2009_Visual cues release	1	1	1	0	1	0	-	-	0	-	-	0
224	Giard_1995_SEPARATE REPRESENTAT	1	1	1	0	1	0	-	-	0	-	-	0
225	Dietz_2014_Effective connectivi	1	1	1	0	1	0	-	-	0	-	-	0
226	David_2006_Dynamic causal model	1	1	1	0	1	0	-	-	0	-	-	0
227	Woods_1993_Intermodal selective	1	1	1	0	1	0	-	-	0	-	-	0
228	Sable_2007_Optical imaging of t	1	1	1	0	1	0	-	-	0	-	-	0
229	Knott_2012_Nicotine, auditory s	1	1	1	0	1	0	-	-	0	-	-	0
230	Duman_2011_Neurocognitive funct	1	1	1	0	1	0	-	-	0	-	-	0
231	Alain_2004_Aging: A Switch from	1	1	1	0	1	0	-	-	0	-	-	0
232	Alain_2002_Deficits in automati	1	1	1	0	1	0	-	-	0	-	-	0
233	Gaeta_1998_An event-related pot	1	1	1	0	1	0	-	-	0	-	-	0
234	Roeber_2003_Auditory distraction	1	1	1	0	1	0	-	-	0	-	-	0
235	Fisher_2018_Auditory Mismatch Ne	1	1	1	0	1	0	-	-	0	-	-	0
236	Jiao_2022_Test-retest reliabil	1	1	1	0	1	0	-	-	0	-	-	0
237	Hisagi_2010_Perception of a Japa	1	1	1	0	1	0	-	-	0	-	-	0
238	Fisher_2008_The right profile: M	1	1	1	0	1	0	-	-	0	-	-	0
239	Kujala_2007_Atypical pattern of	1	1	1	0	1	0	-	-	0	-	-	0
240	Cheour_2002_Electric brain respo	1	1	1	0	1	0	-	-	0	-	-	0
241	Tomé_2013_Mismatch negativity	1	1	1	0	1	0	-	-	0	-	-	0
242	Usui_2023_Longitudinal change	1	1	1	0	1	0	-	-	0	-	-	0
243	Wetzel_2011_Processing of novel	1	1	1	0	1	0	-	-	0	-	-	0
244	Wang_2016_Rubberband effect in	1	1	1	0	1	0	-	-	0	-	-	0
245	Troche_2010_Mental ability and t	1	1	1	0	1	0	-	-	0	-	-	0
246	Tang_2018_Precise theta burst	1	1	1	0	1	0	-	-	0	-	-	0
247	Sumner_2018_Neural plasticity is	1	1	1	0	1	0	-	-	0	-	-	0
248	Kompus_2015_Resting-state glutam	1	1	1	0	1	0	-	-	0	-	-	0
249	Jäncke_2012_Pre-attentive modula	1	1	1	0	1	0	-	-	0	-	-	0
250	Horton_2011_MMN responsivity to	1	1	1	0	1	0	-	-	0	-	-	0
251	Hisagi_2015_Neural measures of a	1	1	1	0	1	0	-	-	0	-	-	0
252	Hay_2015_Equivalent mismatch	1	1	1	0	1	0	-	-	0	-	-	0
253	Hansen_2022_Musicians show more	1	1	1	0	1	0	-	-	0	-	-	0
254	Gilley_2017_Spectral-temporal EE	1	1	1	0	1	0	-	-	0	-	-	0
255	Butler_2011_Common or Redundant	1	1	1	0	1	0	-	-	0	-	-	0
256	Bangel_2017_Aberrant brain respo	1	1	1	0	1	0	-	-	0	-	-	0

257	Althen_2013_Simple and complex a	1	1	1	0	1	0	-	-	0	-	-	0
258	List_2007_A mismatch negativit	1	1	1	0	1	0	-	-	0	-	-	0
259	Fisher_2006_Abnormality of misma	1	1	1	0	1	0	-	-	0	-	-	0
260	Fisher_2012_Alterations of misma	1	1	1	0	1	0	-	-	0	-	-	0
261	Escera_2002_An electrophysiologi	1	1	1	0	1	0	-	-	0	-	-	0
262	Zhao_2018_Automatic processing	1	1	1	0	1	0	-	-	0	-	-	0
263	Fisher_2012_Nicotine and the hal	1	1	1	0	1	0	-	-	0	-	-	0
264	Fisher_2011_Effects of auditory	1	1	1	0	1	0	-	-	0	-	-	0
265	D'Arcy_2004_Separating phonologi	1	1	1	0	1	0	-	-	0	-	-	0
266	Arnott_2002_Stepping out of the	1	1	1	0	1	0	-	-	0	-	-	0
267	Althen_2016_Middle latency respo	1	1	1	0	1	0	-	-	0	-	-	0
268	Wang_2022_Application of audit	1	1	1	0	1	0	-	-	0	-	-	0
269	Todd_2003_Association between	1	1	1	0	1	0	-	-	0	-	-	0
270	Mittag_2013_Audiovisual attentio	1	1	1	0	1	0	-	-	0	-	-	0
271	Kong_2023_Auditory Mismatch Ne	1	1	1	0	1	0	-	-	0	-	-	0
272	Todd_2000_Do perceived loudnes	1	1	1	0	1	0	-	-	0	-	-	0
273	Fisher_2011_Effects of deviant p	1	1	1	0	1	0	-	-	0	-	-	0
274	Hikita_2020_Estimation of freque	1	1	1	0	1	0	-	-	0	-	-	0
275	Gürses_2020_Evaluation of audito	1	1	1	0	1	0	-	-	0	-	-	0
276	Althen_2011_Fast Detection of Un	1	1	1	0	1	0	-	-	0	-	-	0
277	Tateno_2021_Features of Duration	1	1	1	0	1	0	-	-	0	-	-	0
278	Meiser_2024_High-density ear-EEG	1	1	1	0	1	0	-	-	0	-	-	0
279	Altman_2010_How does mismatch ne	1	1	1	0	1	0	-	-	0	-	-	0
280	Liasis_2000_Intracranial evidenc	1	1	1	0	1	0	-	-	0	-	-	0
281	Ding_2006_Mismatch Negativity	1	1	1	0	1	0	-	-	0	-	-	0
282	Fisher_2019_Mismatch negativity-	1	1	1	0	1	0	-	-	0	-	-	0
283	Kirmse_2008_Modulation of the mi	1	1	1	0	1	0	-	-	0	-	-	0
284	Mori_2021_Omission mismatch ne	1	1	1	0	1	0	-	-	0	-	-	0
285	Ylinen_2005_Phoneme quality and	1	1	1	0	1	0	-	-	0	-	-	0
286	Zaehle_2009_Pre-attentive Spectr	1	1	1	0	1	0	-	-	0	-	-	0
287	Zhou_2023_Short-Term Phonemic	1	1	1	0	1	0	-	-	0	-	-	0
288	Jiao_2021_The lateralization a	1	1	1	0	1	0	-	-	0	-	-	0
289	Troche_2009_Mental ability, P300	1	1	1	0	1	0	-	-	0	-	-	0
290	Meng_2021_Mismatch negativity	1	1	1	0	1	0	-	-	0	-	-	0
291	Ylinen_2006_Mismatch negativity	1	1	1	0	1	0	-	-	0	-	-	0
292	Hara_2012_Mismatch negativity	1	1	1	0	1	0	-	-	0	-	-	0
293	Dawson_2016_Quantity language sp	1	1	1	0	1	0	-	-	0	-	-	0

294	Bose_2023_Repetition-dependent	1	1	1	0	1	0	-	-	0	-	-	0
295	Wang_2015_Subjective present:	1	1	1	0	1	0	-	-	0	-	-	0
296	Aitake_2011_Sensory mismatch ind	1	1	1	0	1	0	-	-	0	-	-	0
297	Chennu_2013_Expectation and atte	1	1	1	0	1	0	-	-	0	-	-	0
298	Rinne_2000_Separate time behavi	1	1	1	0	1	0	-	-	0	-	-	0
299	Engstro_2021_Auditory event-relat	1	1	1	0	1	0	-	-	0	-	-	0
300	Halgren_1995_INTRACEREBRAL POTENT	1	1	1	0	1	0	-	-	0	-	-	0
301	Okazaki_2010_Neural substrate of	1	1	1	0	1	0	-	-	0	-	-	0
302	Xiong_2022_Automatic change det	1	1	1	0	1	0	-	-	0	-	-	0
303	Edalati_2021_Violation of rhythmi	1	1	1	0	1	0	-	-	0	-	-	0
304	Deouell_2006_Spatial location is	1	1	1	0	1	0	-	-	0	-	-	0
305	Demeter_2022_Reward magnitude enh	1	1	1	0	1	0	-	-	0	-	-	0
306	Brima_2024_Assessing the integr	1	1	1	0	1	0	-	-	0	-	-	0
307	Kasai_2002_Do high or low doses	1	1	1	0	1	0	-	-	0	-	-	0
308	Baker_2005_COMT Val108/158Met m	1	1	1	0	1	0	-	-	0	-	-	0
309	Bradlow_1999_Effects of lengthene	1	1	1	0	1	0	-	-	0	-	-	0
310	Froud_2012_Mismatch negativity	1	1	1	0	1	0	-	-	0	-	-	0
311	Criel_2023_Pre-to Postoperative	1	1	1	0	1	0	-	-	0	-	-	0
312	Chobert_2014_Twelve Months of Act	1	1	1	0	1	0	-	-	0	-	-	0
313	Urban_2007_Mismatch negativity	1	1	1	0	1	0	-	-	0	-	-	0
314	Wiens_2019_Visual perceptual lo	1	1	1	0	1	0	-	-	0	-	-	0
315	Wiens_2016_Visual task demands	1	1	1	0	1	0	-	-	0	-	-	0
316	Weigl_2016_Transcranial direct	1	1	1	0	1	0	-	-	0	-	-	0
317	Takei_2010_Preattentive dysfunc	1	1	1	0	1	0	-	-	0	-	-	0
318	Knott_2014_Modulation of audito	1	1	1	0	1	0	-	-	0	-	-	0
319	Jahshan_2012_Cross-diagnostic com	1	1	1	0	1	0	-	-	0	-	-	0
320	Impey_2015_Mismatch negativity	1	1	1	0	1	0	-	-	0	-	-	0
321	Impey_2017_Effects of an NMDA a	1	1	1	0	1	0	-	-	0	-	-	0
322	Impey_2017_Effects of transcran	1	1	1	0	1	0	-	-	0	-	-	0
323	Gomes_2000_Mismatch negativity	1	1	1	0	1	0	-	-	0	-	-	0
324	Dunn_2008_Mismatch negativity	1	1	1	0	1	0	-	-	0	-	-	0
325	Deouell_2000_Electrophysiological	1	1	1	0	1	0	-	-	0	-	-	0
326	Colin_2001_The mismatch negativ	1	1	1	0	1	0	-	-	0	-	-	0
327	Chung_2018_Beyond the real worl	1	1	1	0	1	0	-	-	0	-	-	0
328	Chobert_2012_Deficit in the preat	1	1	1	0	1	0	-	-	0	-	-	0
329	Bonetti_2018_Auditory sensory mem	1	1	1	0	1	0	-	-	0	-	-	0
330	Altmann_2013_Preattentive process	1	1	1	0	1	0	-	-	0	-	-	0

331	Knott_2015_An acute dose, rando	1	1	1	0	1	0	-	-	0	-	-	0
332	Jemel_2002_Mismatch negativity	1	1	1	0	1	0	-	-	0	-	-	0
333	Criel_2023_Aging and sex effect	1	1	1	0	1	0	-	-	0	-	-	0
334	Colin_2002_Electrophysiology of	1	1	1	0	1	0	-	-	0	-	-	0
335	Winkler_1999_Brain responses reve	1	1	1	0	1	0	-	-	0	-	-	0
336	Poulton_2022_Can You Hear What's	1	1	1	0	1	0	-	-	0	-	-	0
337	Wiens_2019_Cascade and no-repet	1	1	1	0	1	0	-	-	0	-	-	0
338	Xiao_2018_Detecting violation	1	1	1	0	1	0	-	-	0	-	-	0
339	Batista_2023_Domain-general but n	1	1	1	0	1	0	-	-	0	-	-	0
340	Nenonen_2003_Linguistic relevance	1	1	1	0	1	0	-	-	0	-	-	0
341	Röttger_2007_Mismatch negativity	1	1	1	0	1	0	-	-	0	-	-	0
342	Kasai_2002_No effect of gender	1	1	1	0	1	0	-	-	0	-	-	0
343	Horváth_2010_Omission mismatch ne	1	1	1	0	1	0	-	-	0	-	-	0
344	Czigler_1996_Preattentive auditor	1	1	1	0	1	0	-	-	0	-	-	0
345	Winkler_2005_Preattentive binding	1	1	1	0	1	0	-	-	0	-	-	0
346	Ilvonen_2001_The processing of so	1	1	1	0	1	0	-	-	0	-	-	0
347	Ilvonen_2004_The processing of sp	1	1	1	0	1	0	-	-	0	-	-	0
348	Vuust_2012_The sound of music:	1	1	1	0	1	0	-	-	0	-	-	0
349	Criel_2024_Long-term Functional	1	1	1	0	1	0	-	-	0	-	-	0
350	Colin_2009_Mismatch negativity	1	1	1	0	1	0	-	-	0	-	-	0
351	Iyer_2017_Mismatch negativity	1	1	1	0	1	0	-	-	0	-	-	0
352	Davalos_2003_Mismatch negativity	1	1	1	0	1	0	-	-	0	-	-	0
353	Henrich_2022_Predictive Processin	1	1	1	0	1	0	-	-	0	-	-	0
354	Zhou_2021_Processing neutral t	1	1	1	0	1	0	-	-	0	-	-	0
355	Revheim_2014_Reading Deficits in	1	1	1	0	1	0	-	-	0	-	-	0
356	Nenonen_2005_Speech-sound duratio	1	1	1	0	1	0	-	-	0	-	-	0
357	Shiga_2011_Temporal integration	1	1	1	0	1	0	-	-	0	-	-	0
358	Asano_2015_Temporal integration	1	1	1	0	1	0	-	-	0	-	-	0
359	Amenedo_2000_The accuracy of soun	1	1	1	0	1	0	-	-	0	-	-	0
360	Hermann_2018_Unexpected good outc	1	1	1	0	1	0	-	-	0	-	-	0
361	Daikoku_2023_Neural correlates of	1	1	1	0	1	0	-	-	0	-	-	0
362	Winkler_2006_Object representatio	1	1	1	0	1	0	-	-	0	-	-	0
363	Jahshan_2019_A randomized control	1	1	1	0	1	0	-	-	0	-	-	0
364	Halgren_1995_Intracerebral potent	1	1	1	0	1	0	-	-	0	-	-	0
365	Symonds_2020_Cognitive resources	1	1	1	0	1	0	-	-	0	-	-	0
366	Kalyakin_2009_Extraction of the mi	1	1	1	0	1	0	-	-	0	-	-	0
367	Hertrich_2007_Sequential audiovisu	1	1	1	0	1	0	-	-	0	-	-	0

368	Halliday_2014_Late, not early mism	1	1	1	0	1	0	-	-	0	-	-	0
369	Putkinen_2014_Enhanced development	1	1	1	0	1	0	-	-	0	-	-	0
370	Putkinen_2013_Informal musical act	1	1	1	0	1	0	-	-	0	-	-	0
371	Kärgel_2016_The effect of audito	1	1	1	0	1	0	-	-	0	-	-	0
372	Mohideen_2023_Relationship Between	1	1	1	0	1	0	-	-	0	-	-	0
373	Wanyan_2018_Influence of mental	1	1	1	0	1	0	-	-	0	-	-	0
374	Pinheiro_2017_Does emotion change	1	1	1	0	1	0	-	-	0	-	-	0
375	Höller_2011_Preserved oscillator	1	1	1	0	1	0	-	-	0	-	-	0
376	Heldmann_2019_Processing of Local	1	1	1	0	1	0	-	-	0	-	-	0
377	Getzmann_2013_Does age increase au	1	1	1	0	1	0	-	-	0	-	-	0
378	Getzmann_2015_ERP correlates of au	1	1	1	0	1	0	-	-	0	-	-	0
379	Döring_2016_Mismatch negativity:	1	1	1	0	1	0	-	-	0	-	-	0
380	Campbell_2015_Levels of attention	1	1	1	0	1	0	-	-	0	-	-	0
381	Barkaszi_2016_Extreme Environment	1	1	1	0	1	0	-	-	0	-	-	0
382	Baldeweg_1999_Impaired auditory fr	1	1	1	0	1	0	-	-	0	-	-	0
383	Cooray_2014_A mechanistic model	1	1	1	0	1	0	-	-	0	-	-	0
384	Hauke_2023_Aberrant hierarchica	1	1	1	0	1	0	-	-	0	-	-	0
385	Rinker_2007_Abnormal frequency d	1	1	1	0	1	0	-	-	0	-	-	0
386	Wong_2023_Adaptation and Misma	1	1	1	0	1	0	-	-	0	-	-	0
387	Wong_2024_Adaptation Patterns	1	1	1	0	1	0	-	-	0	-	-	0
388	Guille_2011_Interaction of estro	1	1	1	0	1	0	-	-	0	-	-	0
389	Kathmann_1999_Stability of the mis	1	1	1	0	1	0	-	-	0	-	-	0
390	Engström_2020_Computer-assisted re	1	1	1	0	1	0	-	-	0	-	-	0
391	Rutiku_2024_Assessing mismatch n	1	1	1	0	1	0	-	-	0	-	-	0
392	Yumoto_2005_Audiovisual phonolog	1	1	1	0	1	0	-	-	0	-	-	0
393	Näätänen_2004_Automatic time perce	1	1	1	0	1	0	-	-	0	-	-	0
394	Huang_2023_Dissecting Mismatch	1	1	1	0	1	0	-	-	0	-	-	0
395	Atkinson_2012_Duration Mismatch Ne	1	1	1	0	1	0	-	-	0	-	-	0
396	Nakajima_2021_Duration Mismatch Ne	1	1	1	0	1	0	-	-	0	-	-	0
397	Ochiai_2021_Effect of oxytocin n	1	1	1	0	1	0	-	-	0	-	-	0
398	Spackman_2007_Effects of stimulus	1	1	1	0	1	0	-	-	0	-	-	0
399	Zachau_2005_Extracting rules: ea	1	1	1	0	1	0	-	-	0	-	-	0
400	Schroger_1997_Fast preattentive pr	1	1	1	0	1	0	-	-	0	-	-	0
401	Takegata_2004_Hemispheric processi	1	1	1	0	1	0	-	-	0	-	-	0
402	Umbricht_2000_Ketamine-induced def	1	1	1	0	1	0	-	-	0	-	-	0
403	Morita_2022_Mismatch negativity	1	1	1	0	1	0	-	-	0	-	-	0
404	Herman_2020_Mismatch negativity	1	1	1	0	1	0	-	-	0	-	-	0

405	Schroger_1996_Mismatch response of	1	1	1	0	1	0	-	-	0	-	-	0
406	Getzmann_2014_Neuro-Behavioral Cor	1	1	1	0	1	0	-	-	0	-	-	0
407	Verleger_1991_ON THE REASONS FOR T	1	1	1	0	1	0	-	-	0	-	-	0
408	Takegata_2008_Parameter-specific m	1	1	1	0	1	0	-	-	0	-	-	0
409	McKenzie_2006_The independence of	1	1	1	0	1	0	-	-	0	-	-	0
410	Sendesen_2022_The mismatch negativ	1	1	1	0	1	0	-	-	0	-	-	0
411	Davids_2009_Towards neurophysiol	1	1	1	0	1	0	-	-	0	-	-	0
412	Lindín_2013_Mismatch negativity	1	1	1	0	1	0	-	-	0	-	-	0
413	Herman_2023_Mismatch negativity	1	1	1	0	1	0	-	-	0	-	-	0
414	Bendixen_2014_Prediction in the se	1	1	1	0	1	0	-	-	0	-	-	0
415	Schairer_2001_Source generators of	1	1	1	0	1	0	-	-	0	-	-	0
416	Ringer_2024_That sounds awful! D	1	1	1	0	1	0	-	-	0	-	-	0
417	Goerlich_2012_The Sound of Feeling	1	1	1	0	1	0	-	-	0	-	-	0
418	Verleger_1991_On the reasons for t	1	1	1	0	1	0	-	-	0	-	-	0
419	Bardsley_2014_Implementation of in	1	1	1	0	1	0	-	-	0	-	-	0
420	Heekeren_2008_Mismatch negativity	1	1	1	0	1	0	-	-	0	-	-	0
421	Rasser_2011_Gray matter deficits	1	1	1	0	1	0	-	-	0	-	-	0
422	Czamara_2011_Association of a Rar	1	1	1	0	1	0	-	-	0	-	-	0
423	Norton_2021_ERP mismatch negativ	1	1	1	0	1	0	-	-	0	-	-	0
424	Ceponiene_2002_Event-related potent	1	1	1	0	1	0	-	-	0	-	-	0
425	Corbera_2006_Impaired duration mi	1	1	1	0	1	0	-	-	0	-	-	0
426	Milovanov_2009_The role of musical	1	1	1	0	1	0	-	-	0	-	-	0
427	Ponton_2000_Maturation of the mi	1	1	1	0	1	0	-	-	0	-	-	0
428	Shinozaki_2002_The difference in Mi	1	1	1	0	1	0	-	-	0	-	-	0
429	Wild-Wall_2005_Maturation processes	1	1	1	0	1	0	-	-	0	-	-	0
430	Weckesser_2017_NMDA receptor modula	1	1	1	0	1	0	-	-	0	-	-	0
431	Thiebes_2017_Glutamatergic defici	1	1	1	0	1	0	-	-	0	-	-	0
432	SanMiguel_2008_When loading working	1	1	1	0	1	0	-	-	0	-	-	0
433	Salisbury_2017_Pitch and Duration M	1	1	1	0	1	0	-	-	0	-	-	0
434	Ruzzoli_2012_Sensory memory durin	1	1	1	0	1	0	-	-	0	-	-	0
435	Restuccia_2005_Attentional load of	1	1	1	0	1	0	-	-	0	-	-	0
436	Koistinen_2012_Effects of significa	1	1	1	0	1	0	-	-	0	-	-	0
437	Kaipio_2001_Fast vigilance decre	1	1	1	0	1	0	-	-	0	-	-	0
438	Fulham_2014_Mismatch negativity	1	1	1	0	1	0	-	-	0	-	-	0
439	Cooray_2016_The maturation of mi	1	1	1	0	1	0	-	-	0	-	-	0
440	Bühler_2017_Influence of dialect	1	1	1	0	1	0	-	-	0	-	-	0
441	Andersson_2008_Neuropsychological a	1	1	1	0	1	0	-	-	0	-	-	0

442	Fisher_2010_Light up and see: en	1	1	1	0	1	0	-	-	0	-	-	0
443	Jaramillo_1999_An event-related pot	1	1	1	0	1	0	-	-	0	-	-	0
444	Delussi_2024_Auditory mismatch ne	1	1	1	0	1	0	-	-	0	-	-	0
445	Matsuda_2013_Distinct pre-attenti	1	1	1	0	1	0	-	-	0	-	-	0
446	Osakabe_2020_Do tone duration cha	1	1	1	0	1	0	-	-	0	-	-	0
447	Hoshino_2023_Effect of the tempor	1	1	1	0	1	0	-	-	0	-	-	0
448	Akpınar_2016_Effects of pre-and p	1	1	1	0	1	0	-	-	0	-	-	0
449	Schaadt_2014_Gaining mismatch neg	1	1	1	0	1	0	-	-	0	-	-	0
450	Lawson_1981_Mismatch negativity	1	1	1	0	1	0	-	-	0	-	-	0
451	Winkler_1998_Mismatch negativity:	1	1	1	0	1	0	-	-	0	-	-	0
452	Sarasso_2022_Nice and easy: Misma	1	1	1	0	1	0	-	-	0	-	-	0
453	Sonnadara_2006_Occasional changes i	1	1	1	0	1	0	-	-	0	-	-	0
454	Salisbury_2020_Pitch and Duration M	1	1	1	0	1	0	-	-	0	-	-	0
455	Chládková_2013_Pre-attentive sensit	1	1	1	0	1	0	-	-	0	-	-	0
456	Paukkunen_2011_The effect of measur	1	1	1	0	1	0	-	-	0	-	-	0
457	Chládková_2015_When AA is long but	1	1	1	0	1	0	-	-	0	-	-	0
458	Saloranta_2020_Listen-and-repeat tr	1	1	1	0	1	0	-	-	0	-	-	0
459	Saloranta_2022_Listen-and-repeat tr	1	1	1	0	1	0	-	-	0	-	-	0
460	Miyanishi_2013_LORETA Current Sourc	1	1	1	0	1	0	-	-	0	-	-	0
461	Tiainen_2017_Mismatch negativity	1	1	1	0	1	0	-	-	0	-	-	0
462	Jaramillo_2000_Mismatch negativity	1	1	1	0	1	0	-	-	0	-	-	0
463	Aeberli_2023_Mismatch negativity	1	1	1	0	1	0	-	-	0	-	-	0
464	Yagcioglu_2006_The 'Franssen' illus	1	1	1	0	1	0	-	-	0	-	-	0
465	Micheyl_2003_The neurophysiologic	1	1	1	0	1	0	-	-	0	-	-	0
466	Fukushima_2010_Neural correlates of	1	1	1	0	1	0	-	-	0	-	-	0
467	Carminati_2018_Neurophysiological d	1	1	1	0	1	0	-	-	0	-	-	0
468	Salisbury_2020_Pitch and Duration M	1	1	1	0	1	0	-	-	0	-	-	0
469	Armstrong_2011_Neural markers of au	1	1	1	0	1	0	-	-	0	-	-	0
470	Nakamura_2011_Epidural auditory ev	1	1	1	0	1	0	-	-	0	-	-	0
471	Eskelund_2015_Face configuration a	1	1	1	0	1	0	-	-	0	-	-	0
472	Sanctis_2009_Right hemispheric co	1	1	1	0	1	0	-	-	0	-	-	0
473	Kalyakin_2008_Independent componen	1	1	1	0	1	0	-	-	0	-	-	0
474	Shestakova_2002_Involuntary attentio	1	1	1	0	1	0	-	-	0	-	-	0
475	Friederici_2002_Neural manifestation	1	1	1	0	1	0	-	-	0	-	-	0
476	Kalyakin_2007_Optimal digital filt	1	1	1	0	1	0	-	-	0	-	-	0
477	Schultheis_2022_Quantitative electro	1	1	1	0	1	0	-	-	0	-	-	0
478	Tervaniemi_1999_Test-retest reliabil	1	1	1	0	1	0	-	-	0	-	-	0

479	Kaganovich_2021_Event-related potent	1	1	1	0	1	0	-	-	0	-	-	0
480	Horvath_2013_Does sight predomina	1	1	1	0	1	0	-	-	0	-	-	0
481	Deouell_1998_Mismatch negativity	1	1	1	0	1	0	-	-	0	-	-	0
482	Campbell_2007_N1 and the mismatch	1	1	1	0	1	0	-	-	0	-	-	0
483	Cacciaglia_2019_Auditory predictions	1	1	1	0	1	0	-	-	0	-	-	0
484	Andres_2011_Congruency of audito	1	1	1	0	1	0	-	-	0	-	-	0
485	Altmann_2012_Allocentric or crani	1	1	1	0	1	0	-	-	0	-	-	0
486	Haumann_2023_Mismatch negativity	1	1	1	0	1	0	-	-	0	-	-	0
487	FrodlBauch_1997_Dipole localization	1	1	1	0	1	0	-	-	0	-	-	0
488	Rentzsch_2015_Auditory mismatch ne	1	1	1	0	1	0	-	-	0	-	-	0
489	Tervaniemi_2000_Effects of spectral	1	1	1	0	1	0	-	-	0	-	-	0
490	Schröger_2021_Encoding of determin	1	1	1	0	1	0	-	-	0	-	-	0
491	Takegata_1999_Independent processi	1	1	1	0	1	0	-	-	0	-	-	0
492	Cornell_2013_Inequality across co	1	1	1	0	1	0	-	-	0	-	-	0
493	Campbell_2007_N1 and mismatch-nega	1	1	1	0	1	0	-	-	0	-	-	0
494	Ruusuvirta_2016_Preattentive and att	1	1	1	0	1	0	-	-	0	-	-	0
495	Parmentier_2010_The Involuntary Capt	1	1	1	0	1	0	-	-	0	-	-	0
496	Bartolomeo_2019_Relationship of audi	1	1	1	0	1	0	-	-	0	-	-	0
497	Randazzo_2023_The Audiovisual Mism	1	1	1	0	1	0	-	-	0	-	-	0
498	Vollmer_2023_The Auditory Mismatc	1	1	1	0	1	0	-	-	0	-	-	0
499	Astikainen_2001_Somatosensory event-	1	1	1	0	1	0	-	-	0	-	-	0
500	Ruusuvirta_1996_Multiple-unit respon	1	1	1	0	1	0	-	-	0	-	-	0
501	Hirschfeld_2011_Effects of language	1	1	1	0	1	0	-	-	0	-	-	0
502	van Rhijn_2013_Can eye of origin se	1	1	1	0	1	0	-	-	0	-	-	0
503	Duda-Milloy_2019_A time-efficient mul	1	1	1	0	1	0	-	-	0	-	-	0
504	Petermann_2009_Statistical detectio	1	1	1	0	1	0	-	-	0	-	-	0
505	Joutsiniemi_1998_The mismatch negativ	1	1	1	0	1	0	-	-	0	-	-	0
506	Huotilainen_2006_Magnetoencephalograp	1	1	1	0	1	0	-	-	0	-	-	0
507	Giordano_2021_Mismatch negativity	1	1	1	0	1	0	-	-	0	-	-	0
508	Devrim-Üçok_2008_Mismatch negativity	1	1	1	0	1	0	-	-	0	-	-	0
509	Corcoran_2018_Developmental trajec	1	1	1	0	1	0	-	-	0	-	-	0
510	Fuentemilla_2008_Theta EEG oscillator	1	1	1	0	1	0	-	-	0	-	-	0
511	Cummings_2019_Effect of deviance d	1	1	1	0	1	0	-	-	0	-	-	0
512	Kropotov_1997_Intracranial mismatc	1	1	1	0	1	0	-	-	0	-	-	0
513	PAAVILAINEN_1989_MISMATCH NEGATIVITY	1	1	1	0	1	0	-	-	0	-	-	0
514	Paavilainen_2007_Preattentive detecti	1	1	1	0	1	0	-	-	0	-	-	0
515	PAAVILAINEN_1993_STIMULUS-DURATION AN	1	1	1	0	1	0	-	-	0	-	-	0

516	Pakarinen_2010_The mismatch negativ	1	1	1	0	1	0	-	-	0	-	-	0
517	Elhassanien_2020_Essential tremor: wh	1	1	1	0	1	0	-	-	0	-	-	0
518	Khodanovich_2009_Visual analog of mis	1	1	1	0	1	0	-	-	0	-	-	0
519	Bissonnette_2020_MMN-Indexed Auditory	1	1	1	0	1	0	-	-	0	-	-	0
520	Donaldson_2023_Mismatch negativity	1	1	1	0	1	0	-	-	0	-	-	0
521	Datta Ph D_2010_Native language expe	1	1	1	0	1	0	-	-	0	-	-	0
522	Stekelenburg_2009_Neural correlates of	1	1	1	0	1	0	-	-	0	-	-	0
523	Isenstein_2024_Intact Somatosensory	1	1	1	0	1	0	-	-	0	-	-	0
524	Donaldson_2020_Associations of mism	1	1	1	0	1	0	-	-	0	-	-	0
525	Szychowska_2017_Effects of sound pre	1	1	1	0	1	0	-	-	0	-	-	0
526	Shestopalova_2016_Hemispheric asymmetr	1	1	1	0	1	0	-	-	0	-	-	0
527	Szymanski_1999_Phonemes, intensity	1	1	1	0	1	0	-	-	0	-	-	0
528	Ullsperger_2006_When sound and pictu	1	1	1	0	1	0	-	-	0	-	-	0
529	Donaldson_2021_Mismatch negativity	1	1	1	0	1	0	-	-	0	-	-	0
530	Isenstein_2024_Probing the Neurophy	1	1	1	0	1	0	-	-	0	-	-	0
531	De Pascalis_2014_Mental ability and i	1	1	1	0	1	0	-	-	0	-	-	0
532	Kilpeläinen_1999_Reduced mismatch neg	1	1	1	0	1	0	-	-	0	-	-	0
533	Saint-Amour_2007_Seeing voices: High-	1	1	1	0	1	0	-	-	0	-	-	0
534	Korostenskaja_2010_Neuromagnetic eviden	1	1	1	0	1	0	-	-	0	-	-	0
535	De La Salle_2019_NMDA receptor antago	1	1	1	0	1	0	-	-	0	-	-	0
536	Korostenskaja_2007_Effects of NMDA rece	1	1	1	0	1	0	-	-	0	-	-	0
537	Paavilainen_2003_Evidence for the dif	1	1	1	0	1	0	-	-	0	-	-	0
538	Zion-Golumbic_2007_Representation of ha	1	1	1	0	1	0	-	-	0	-	-	0
539	Bruggemann_2013_Mismatch negativity	1	1	1	0	1	0	-	-	0	-	-	0
540	Paavilainen_2001_The additivity of th	1	1	1	0	1	0	-	-	0	-	-	0
541	Correa-Jaraba_2016_Involuntary capture	1	1	1	0	1	0	-	-	0	-	-	0
542	Korostenskaja_2008_The effect of methyl	1	1	1	0	1	0	-	-	0	-	-	0
543	Huttunen-Scott_2008_Mismatch negativity	1	1	1	0	1	0	-	-	0	-	-	0
544	Herrera-Diaz_2023_Tracking auditory mi	1	1	1	0	1	0	-	-	0	-	-	0
545	Chandrasekaran_2007_Experience-dependent	1	1	1	0	1	0	-	-	0	-	-	0
546	Evstigneeva_2010_Muscle contraction f	1	1	1	0	1	0	-	-	0	-	-	0
547	Aleksandrov_2016_Mismatch negativity	1	1	1	0	1	0	-	-	0	-	-	0
548	Stekelenburg_2018_Multisensory integra	1	1	1	0	1	0	-	-	0	-	-	0
549	Abalo-Rodríguez_2023_Pavlovian conditioni	1	1	1	0	1	0	-	-	0	-	-	0
550	Dittmann-Balcar_1999_Attention-dependent	1	1	1	0	1	0	-	-	0	-	-	0
551	Stekelenburg_2004_Illusory sound shift	1	1	1	0	1	0	-	-	0	-	-	0
552	Ruiz-Martínez_2020_Impaired P1 habituat	1	1	1	0	1	0	-	-	0	-	-	0

553	Shankarnarayan_2007_Mismatch negativity	1	1	1	0	1	0	-	-	0	-	-	0
554	Régio Brambilla_2022_mGluR5 binding chang	1	1	1	0	1	0	-	-	0	-	-	0
555	Quiroga-Martinez_2021_Enhanced mismatch	1	1	1	0	1	0	-	-	0	-	-	0
556	Studer-Eichenberger_2016_Statistical learning	1	1	1	0	1	0	-	-	0	-	-	0
557	del_2020_Abnormal Frequency M	1	1	1	0	1	0	-	-	0	-	-	0
558	Tervaniemi_1999_Pre-attentive discri	1	1	1	0	1	0	-	-	0	-	-	0
559	Hamilton_2018_Interactive effects	1	1	1	0	1	0	-	-	0	-	-	0
560	Chernyshev_2016_Distributed feature	1	1	1	0	1	0	-	-	0	-	-	0
561	Bernstein_2001_ELECTROPHYSIOLOGY OF	1	1	1	0	1	0	-	-	0	-	-	0
562	Cummings_2019_Mismatch Negativity	1	1	1	0	1	0	-	-	0	-	-	0
563	Kaipio_2016_Mismatch negativity	1	1	1	0	1	0	-	-	0	-	-	0
564	Sittiprapaporn_2012_An Effect of Attenti	1	1	1	0	1	0	-	-	0	-	-	0
565	Perry_2012_Brain mapping of the	1	1	1	0	1	0	-	-	0	-	-	0
566	Muller-Gass_2004_The effect of task d	1	1	1	0	1	0	-	-	0	-	-	0
567	Moberget_2007_Sensory prediction o	1	1	1	0	1	0	-	-	0	-	-	0
568	Deouell_2007_The frontal generato	1	1	1	0	1	0	-	-	0	-	-	0
569	Qi_2022_Evidence for predict	1	1	1	0	-	0	-	-	0	-	-	0
570	Ng_2014_Probing Interval Tim	1	1	1	0	-	0	-	-	0	-	-	0
571	Li_2023_Auditory event-relat	1	1	1	0	-	0	-	-	0	-	-	0
572	Ono_2013_Effects of regularit	1	1	1	0	-	0	-	-	0	-	-	0
573	May_2021_The Adaptation Model	1	1	1	0	-	0	-	-	0	-	-	0
574	Liu_2015_Cultural differences	1	1	1	0	-	0	-	-	0	-	-	0
575	Lin_2007_Plastic phase-lockin	1	1	1	0	-	0	-	-	0	-	-	0
576	Lee_2018_A tale of two sites:	1	1	1	0	-	0	-	-	0	-	-	0
577	Zou_2014_Discharge characteri	1	1	1	0	-	0	-	-	0	-	-	0
578	Lee_2012_Mismatch responses t	1	1	1	0	-	0	-	-	0	-	-	0
579	Kwon_2009_Preattentive Auditor	1	1	1	0	-	0	-	-	0	-	-	0
580	ALHO_1995_CEREBRAL GENERATORS	1	1	1	0	-	0	-	-	0	-	-	0
581	Frey_2019_Music training posit	1	1	1	0	-	0	-	-	0	-	-	0
582	Inaba_2020_Sound frequency depe	1	1	1	0	-	0	-	-	0	-	-	0
583	Prete_2022_The sound of silence	1	1	1	0	-	0	-	-	0	-	-	0
584	Nagai_2013_Auditory mismatch ne	1	1	1	0	-	0	-	-	0	-	-	0
585	Munka_2006_Examining task-depen	1	1	1	0	-	0	-	-	0	-	-	0
586	Lee_2017_Neural mechanisms of	1	1	1	0	-	0	-	-	0	-	-	0
587	Glass_2009_Event-related potent	1	1	1	0	-	0	-	-	0	-	-	0
588	Glass_2008_Auditory sensory mem	1	1	1	0	-	0	-	-	0	-	-	0
589	Zhong_2020_Comparison of sound	1	1	1	0	-	0	-	-	0	-	-	0

590	Sabri_2000_Mismatch negativity	1	1	1	0	-	0	-	-	0	-	-	0
591	Serra_1996_The H1-receptor anta	1	1	1	0	-	0	-	-	0	-	-	0
592	Mager_2005_Neurophysiological a	1	1	1	0	-	0	-	-	0	-	-	0
593	Müller_2002_Cortical activation	1	1	1	0	-	0	-	-	0	-	-	0
594	Lewald_2018_Cortical processing	1	1	1	0	-	0	-	-	0	-	-	0
595	Müller_2005_Deviance-repetition	1	1	1	0	-	0	-	-	0	-	-	0
596	Shen_2018_The somatosensory mi	1	1	1	0	-	0	-	-	0	-	-	0
597	Shen_2020_Body representation	1	1	1	0	-	0	-	-	0	-	-	0
598	Schall_2003_Functional neuroanat	1	1	1	0	-	0	-	-	0	-	-	0
599	Qiao_2013_Impaired pre-attenti	1	1	1	0	-	0	-	-	0	-	-	0
600	Nordby_1994_ERPS FOR INFREQUENT	1	1	1	0	-	0	-	-	0	-	-	0
601	Murphy_2013_Reduced duration mis	1	1	1	0	-	0	-	-	0	-	-	0
602	Mittag_2013_The neurophysiologic	1	1	1	0	-	0	-	-	0	-	-	0
603	Michie_2002_Duration mismatch ne	1	1	1	0	-	0	-	-	0	-	-	0
604	Michie_2000_Duration and frequen	1	1	1	0	-	0	-	-	0	-	-	0
605	Ludwig_2012_Age-related dissocia	1	1	1	0	-	0	-	-	0	-	-	0
606	Lauzon_2022_The relationship bet	1	1	1	0	-	0	-	-	0	-	-	0
607	Kujala_2010_Speech-feature discr	1	1	1	0	-	0	-	-	0	-	-	0
608	Kujala_1995_AUDITORY AND SOMATOS	1	1	1	0	-	0	-	-	0	-	-	0
609	Dickey_2008_Auditory processing	1	1	1	0	-	0	-	-	0	-	-	0
610	Kudo_2006_Comparison between m	1	1	1	0	-	0	-	-	0	-	-	0
611	Rue_2021_Contrast and Conflic	1	1	1	0	-	0	-	-	0	-	-	0
612	Sharma_2004_Effects of identific	1	1	1	0	-	0	-	-	0	-	-	0
613	Ponton_1997_Integrated mismatch	1	1	1	0	-	0	-	-	0	-	-	0
614	Müller_2002_Cortical activation	1	1	1	0	-	0	-	-	0	-	-	0
615	Froyen_2009_The long road to aut	1	1	1	0	-	0	-	-	0	-	-	0
616	Simoens_2007_Psychosocial stress	1	1	1	0	-	0	-	-	0	-	-	0
617	Molholm_2001_The detection of con	1	1	1	0	-	0	-	-	0	-	-	0
618	Milovan_2004_ERP study of pre-att	1	1	1	0	-	0	-	-	0	-	-	0
619	Mathiak_2005_Selective influences	1	1	1	0	-	0	-	-	0	-	-	0
620	Mathiak_2000_Encoding of temporal	1	1	1	0	-	0	-	-	0	-	-	0
621	Magno_2008_Are auditory-evoked	1	1	1	0	-	0	-	-	0	-	-	0
622	MacLean_2014_Temporo-frontal phas	1	1	1	0	-	0	-	-	0	-	-	0
623	Lui_2021_The critical role of	1	1	1	0	-	0	-	-	0	-	-	0
624	Lidji_2010_Early integration of	1	1	1	0	-	0	-	-	0	-	-	0
625	Lappe_2013_A beamformer analysi	1	1	1	0	-	0	-	-	0	-	-	0
626	Okazaki_2006_Change detection and	1	1	1	0	-	0	-	-	0	-	-	0

627	Carlyon_2009_Changes in the Perce	1	1	1	0	-	0	-	-	0	-	-	0
628	Vuust_2016_Comprehensive audito	1	1	1	0	-	0	-	-	0	-	-	0
629	Johnson_2006_Differential cortica	1	1	1	0	-	0	-	-	0	-	-	0
630	Pato_2002_Mismatch negativity	1	1	1	0	-	0	-	-	0	-	-	0
631	Poulton_2022_Can You Hear What’s	1	1	1	0	-	0	-	-	0	-	-	0
632	Nichols_2016_Neural correlates of	1	1	1	0	-	0	-	-	0	-	-	0
633	Mohebbi_2019_The potential role o	1	1	1	0	-	0	-	-	0	-	-	0
634	Lipponen_2019_Auditory-evoked pote	1	1	1	0	-	0	-	-	0	-	-	0
635	Smith_2015_The separate and com	1	1	1	0	-	0	-	-	0	-	-	0
636	Schirmer_2008_When vocal processin	1	1	1	0	-	0	-	-	0	-	-	0
637	Schirmer_2016_Detecting Temporal C	1	1	1	0	-	0	-	-	0	-	-	0
638	Light_2015_Validation of mismat	1	1	1	0	-	0	-	-	0	-	-	0
639	Lieder_2013_A neurocomputational	1	1	1	0	-	0	-	-	0	-	-	0
640	Lieder_2013_Modelling trial-by-t	1	1	1	0	-	0	-	-	0	-	-	0
641	Ludlow_2014_Auditory processing	1	1	1	0	-	0	-	-	0	-	-	0
642	Takegata_2001_Changes in acoustic	1	1	1	0	-	0	-	-	0	-	-	0
643	Hirose_2014_Changes in the durat	1	1	1	0	-	0	-	-	0	-	-	0
644	Jacobsen_2003_Measuring duration m	1	1	1	0	-	0	-	-	0	-	-	0
645	Orekhova_2014_Arousal and attentio	1	1	1	0	-	0	-	-	0	-	-	0
646	Lakatos_2020_The thalamocortical	1	1	1	0	-	0	-	-	0	-	-	0
647	Sonnadara_2006_Effects of spatial s	1	1	1	0	-	0	-	-	0	-	-	0
648	Sehatpour_2021_Deficits in Pre-atte	1	1	1	0	-	0	-	-	0	-	-	0
649	Sayfulina_2019_FEATURE BINDING IN V	1	1	1	0	-	0	-	-	0	-	-	0
650	Picton_2000_Mismatch negativity:	1	1	1	0	-	0	-	-	0	-	-	0
651	Oknina_2005_Frontal and temporal	1	1	1	0	-	0	-	-	0	-	-	0
652	Molholm_2005_The neural circuitry	1	1	1	0	-	0	-	-	0	-	-	0
653	Menning_2008_Reduced mismatch neg	1	1	1	0	-	0	-	-	0	-	-	0
654	Koshiyama_2017_Duration and frequen	1	1	1	0	-	0	-	-	0	-	-	0
655	Koshiyama_2018_Association between	1	1	1	0	-	0	-	-	0	-	-	0
656	Southwell_2018_Enhanced deviant res	1	1	1	0	-	0	-	-	0	-	-	0
657	Plumridge_2020_The Effect of Visual	1	1	1	0	-	0	-	-	0	-	-	0
658	MacGregor_2010_Listening to the sou	1	1	1	0	-	0	-	-	0	-	-	0
659	Ruusuvirta_2013_Auditory Cortical an	1	1	1	0	-	0	-	-	0	-	-	0
660	Scharinger_2017_Integrating speech i	1	1	1	0	-	0	-	-	0	-	-	0
661	Pfingst_2012_The fusion of unatte	1	1	1	0	-	0	-	-	0	-	-	0
662	Näätänen_2014_Mismatch negativity	1	1	1	0	-	0	-	-	0	-	-	0
663	Moberget_2008_Detecting violations	1	1	1	0	-	0	-	-	0	-	-	0

664	Miyajima_2011_Abnormal mismatch ne	1	1	1	0	-	0	-	-	0	-	-	0
665	Lewendon_2023_The MMN by another n	1	1	1	0	-	0	-	-	0	-	-	0
666	Lazarev_2018_Feature binding in a	1	1	1	0	-	0	-	-	0	-	-	0
667	Sculthorpe_2008_The influence of str	1	1	1	0	-	0	-	-	0	-	-	0
668	Parmentier_2009_The involuntary capt	1	1	1	0	-	0	-	-	0	-	-	0
669	Manousakis_2009_Quantum formalism to	1	1	1	0	-	0	-	-	0	-	-	0
670	Mahmoudian_2015_Alterations in audit	1	1	1	0	-	0	-	-	0	-	-	0
671	Kuchenbuch_2014_Audio-tactile integr	1	1	1	0	-	0	-	-	0	-	-	0
672	Vestergaard_2009_Auditory size-devian	1	1	1	0	-	0	-	-	0	-	-	0
673	Muller-Gass_2007_Evidence for the aud	1	1	1	0	-	0	-	-	0	-	-	0
674	Shestopalova_2012_Discrimination of au	1	1	1	0	-	0	-	-	0	-	-	0
675	Shestopalova_2021_PHASE COHERENCE OF T	1	1	1	0	-	0	-	-	0	-	-	0
676	Shestopalova_2015_Contextual effects o	1	1	1	0	-	0	-	-	0	-	-	0
677	Muller-Gass_2006_The effect of visual	1	1	1	0	-	0	-	-	0	-	-	0
678	Pulvermüller_2003_Automatic processing	1	1	1	0	-	0	-	-	0	-	-	0
679	Pattamadilok_2014_Unattentive speech p	1	1	1	0	-	0	-	-	0	-	-	0
680	Martínez-Montes_2013_Musical expertise an	1	1	1	0	-	0	-	-	0	-	-	0
681	López-Caballero_2016_Differential deviant	1	1	1	0	-	0	-	-	0	-	-	0
682	Grossheinrich_2010_Auditory sensory mem	1	1	1	0	-	0	-	-	0	-	-	0
683	Lopez-Caballero_2024_Computational Synapt	1	1	1	0	-	0	-	-	0	-	-	0
684	Quiroga-Martinez_2022_Enhanced mismatch	1	1	1	0	-	0	-	-	0	-	-	0
685	Erlbeck_2015_The event-related po	1	1	1	0	-	0	-	-	0	-	-	0
686	Grisoni_2012_The sound of actions	1	1	1	0	-	0	-	-	0	-	-	0
687	Wu_2024_EEG Functional Conne	1	1	1	1	0	0	-	-	0	-	-	0
688	Kogai_2011_Visual mismatch resp	1	1	1	1	0	0	-	-	0	-	-	0
689	Susac_2014_Magnetic source loca	1	1	1	1	0	0	-	-	0	-	-	0
690	Vuong_2005_The role of surface	1	1	1	1	0	0	-	-	0	-	-	0
691	Kurita_2023_Theta phase coherenc	1	1	1	1	0	0	-	-	0	-	-	0
692	Urakawa_2010_Cortical dynamics of	1	1	1	1	0	0	-	-	0	-	-	0
693	Urakawa_2017_Exogenously-driven p	1	1	1	1	0	0	-	-	0	-	-	0
694	Kadosh_2023_Fixation-related vis	1	1	1	1	0	0	-	-	0	-	-	0
695	Vinken_2017_Recent Visual Experi	1	1	1	1	-	0	-	-	0	-	-	0
696	Kim_2008_Language lateralizat	1	1	1	-	0	0	-	-	0	-	-	0
697	Adjedj_2018_Comparison of corona	1	1	1	-	0	0	-	-	0	-	-	0
698	Komatsu_2015_Mismatch negativity	1	1	1	-	1	0	-	-	0	-	-	0
699	Van_2023_Robust multisensory	1	1	1	1	1	1	0	0	0	-	-	0
700	Cheng_2023_Impaired pre-synapti	1	1	1	1	1	1	0	0	0	-	-	0

701	Attinger_2017_Visuomotor Coupling	1	1	1	1	1	1	0	0	0	-	-	0
702	Xia_2019_Bi-lateralized Whorf	1	1	1	1	1	1	1	0	0	-	-	0
703	KML_2013_Neurobiology of sens	1	1	1	1	1	1	1	0	0	-	-	0
704	Japaridze_2014_Altered information	1	1	1	1	1	1	1	0	0	-	-	0
705	Kaganovich_2021_Impaired Audiovisual	1	1	1	1	1	1	1	0	0	-	-	0
706	Liu_2010_The influence on cog	1	1	1	1	1	1	1	1	1	0	-	0
707	Wang_2004_The sequential proce	1	1	1	1	1	1	1	1	1	0	-	0
708	Beer_2004_Attention to motion	1	1	1	1	1	1	1	1	1	0	-	0
709	Yin_2018_The dysfunction of p	1	1	1	1	1	1	1	1	1	0	-	0
710	Zhang_2005_Distinctive conflict	1	1	1	1	1	1	1	1	1	0	-	0
711	Wetzel_2007_Cognitive control of	1	1	1	1	1	1	1	1	1	0	-	0
712	Hsu_2011_Arithmetic mismatch	1	1	1	1	1	1	1	1	1	0	-	0
713	Wright_2018_Luminance-polarity d	1	1	1	1	1	1	1	1	1	0	-	0
714	Chen_2017_Mismatch negativity	1	1	1	1	1	1	1	1	1	0	-	0
715	Javitt_2000_Deficits in auditory	1	1	1	1	1	1	1	1	1	0	-	0
716	Altmann_2009_Processing of Audito	1	1	1	1	1	1	1	1	1	0	-	0
717	Gibbons_2011_Percept-based and ob	1	1	1	1	1	1	1	1	1	0	-	0
718	Dieciuc_2017_Changing similarity:	1	1	1	1	1	1	1	1	1	0	-	0
719	Fuggetta_2015_An electrophysiologi	1	1	1	1	1	1	1	1	1	0	-	0
720	Schreiber_2012_Age effects on adjus	1	1	1	1	1	1	1	1	1	0	-	0
721	Friedman_2012_Differential relatio	1	1	1	1	1	1	1	1	1	0	-	0
722	Lasaponara_2018_EEG Correlates of Pr	1	1	1	1	1	1	1	1	1	0	-	0
723	Li_2018_Female Advantage in	1	1	1	1	1	1	1	1	1	1	0	0
724	Li_2022_Emotional bias of tr	1	1	1	1	1	1	1	1	1	1	0	0
725	Sun_2022_Neural correlates of	1	1	1	1	1	1	1	1	1	1	0	0
726	Liu_2017_Cultural immersion a	1	1	1	1	1	1	1	1	1	1	0	0
727	Wang_2023_Automatic processing	1	1	1	1	1	1	1	1	1	1	0	0
728	Zhao_2006_Visual mismatch nega	1	1	1	1	1	1	1	1	1	1	0	0
729	Wang_2022_Configural but Not F	1	1	1	1	1	1	1	1	1	1	0	0
730	Li_2012_Visual mismatch nega	1	1	1	1	1	1	1	1	1	1	0	0
731	Hu_2020_Visual mismatch nega	1	1	1	1	1	1	1	1	1	1	0	0
732	Li_2023_Impact of hazard typ	1	1	1	1	1	1	1	1	1	1	0	0
733	Wu_2017_Negative bias in exp	1	1	1	1	1	1	1	1	1	1	0	0
734	Arad_2019_A neuromarker of cli	1	1	1	1	1	1	1	1	1	1	0	0
735	Zhang_2018_Gender Differences i	1	1	1	1	1	1	1	1	1	1	0	0
736	Wei_2018_Early lexical proces	1	1	1	1	1	1	1	1	1	1	0	0
737	Wei_2019_Processing of task-i	1	1	1	1	1	1	1	1	1	1	0	0

738	Lyyra_2012_Explicit behavioral	1	1	1	1	1	1	1	1	1	1	0	0
739	Liu_2016_Automatic change det	1	1	1	1	1	1	1	1	1	1	0	0
740	Hesse_2017_Preattentive Process	1	1	1	1	1	1	1	1	1	1	0	0
741	Clery_2012_Electrophysiological	1	1	1	1	1	1	1	1	1	1	0	0
742	Singh_2020_Intraindividual Comp	1	1	1	1	1	1	1	1	1	1	0	0
743	Guo_2020_Pre-attentive dysfun	1	1	1	1	1	1	1	1	1	1	0	0
744	Szucs_2007_Electroencephalograp	1	1	1	1	1	1	1	1	1	1	0	0
745	Chang_2010_Dysfunction of proce	1	1	1	1	1	1	1	1	1	1	0	0
746	Blais_2016_ERP evidence for imp	1	1	1	1	1	1	1	1	1	1	0	0
747	File_2022_Automatic change det	1	1	1	1	1	1	1	1	1	1	0	0
748	Ding_2022_The automatic detect	1	1	1	1	1	1	1	1	1	1	0	0
749	Zhou_2015_An Optimized BCI Sys	1	1	1	1	1	1	1	1	1	1	0	0
750	Zeng_2022_Visual mismatch nega	1	1	1	1	1	1	1	1	1	1	0	0
751	Song_2018_The dysfunction of p	1	1	1	1	1	1	1	1	1	1	0	0
752	Müller_2010_Visual Object Repres	1	1	1	1	1	1	1	1	1	1	0	0
753	Ding_2019_Automatic change det	1	1	1	1	1	1	1	1	1	1	0	0
754	Deng_2020_Lexical processing i	1	1	1	1	1	1	1	1	1	1	0	0
755	Chen_2020_Consciousness modula	1	1	1	1	1	1	1	1	1	1	0	0
756	Tang_2013_Visual mismatch nega	1	1	1	1	1	1	1	1	1	1	0	0
757	Chen_2022_Visually Perceived N	1	1	1	1	1	1	1	1	1	1	0	0
758	Suzuki_2024_Contingency learning	1	1	1	1	1	1	1	1	1	1	0	0
759	Parise_2010_Processing faces in	1	1	1	1	1	1	1	1	1	1	0	0
760	Flynn_2009_Can illusory deviant	1	1	1	1	1	1	1	1	1	1	0	0
761	Zhang_2018_Sex and physiologica	1	1	1	1	1	1	1	1	1	1	0	0
762	Zeng_2022_Belief in biological	1	1	1	1	1	1	1	1	1	1	0	0
763	Wascher_2020_No effect of target	1	1	1	1	1	1	1	1	1	1	0	0
764	Wang_2013_Rapid extraction of	1	1	1	1	1	1	1	1	1	1	0	0
765	Vroomen_2014_A bias-free two-alte	1	1	1	1	1	1	1	1	1	1	0	0
766	Petro_2023_The role of attentio	1	1	1	1	1	1	1	1	1	1	0	0
767	Oxner_2019_Prediction errors in	1	1	1	1	1	1	1	1	1	1	0	0
768	Neuhaus_2013_Evidence for impaire	1	1	1	1	1	1	1	1	1	1	0	0
769	Beck_2021_Do categorical repre	1	1	1	1	1	1	1	1	1	1	0	0
770	Amado_2016_Does surprise enhanc	1	1	1	1	1	1	1	1	1	1	0	0
771	Ford_2022_The relationship bet	1	1	1	1	1	1	1	1	1	1	0	0
772	Zhang_2019_Superiority effect i	1	1	1	1	1	1	1	1	1	1	0	0
773	Kimura_2015_Top-down Control ove	1	1	1	1	1	1	1	1	1	1	0	0
774	Olivares_2003_Long-latency ERPs an	1	1	1	1	1	1	1	1	1	1	0	0

775	Files_2013_The visual mismatch	1	1	1	1	1	1	1	1	1	1	0	0
776	Stothart_2016_Neural correlates of	1	1	1	1	1	1	1	1	1	1	0	0
777	Menzel_2018_Visual mismatch nega	1	1	1	1	1	1	1	1	1	1	0	0
778	Khvostov_2021_Spatially intermixed	1	1	1	1	1	1	1	1	1	1	0	0
779	Müller_2013_Object-related regul	1	1	1	1	1	1	1	1	1	1	0	0
780	Kaffes_2023_Impact of emotional	1	1	1	1	1	1	1	1	1	1	0	0
781	Csikós_2024_Automatic Change Det	1	1	1	1	1	1	1	1	1	1	0	0
782	Sultson_2019_Hunger enhances auto	1	1	1	1	1	1	1	1	1	1	0	0
783	Ponton_2009_Mismatch negativity	1	1	1	1	1	1	1	1	1	1	0	0
784	Lacroix_2022_The predictive role	1	1	1	1	1	1	1	1	1	1	0	0
785	Czigler_2014_Asymmetry of automat	1	1	1	1	1	1	1	1	1	1	0	0
786	Czigler_2006_ERPs and deviance de	1	1	1	1	1	1	1	1	1	1	0	0
787	Csizmadia_2021_Older Adults Automat	1	1	1	1	1	1	1	1	1	1	0	0
788	Stefanics_2012_Automatic prediction	1	1	1	1	1	1	1	1	1	1	0	0
789	Kovarski_2017_Facial expression re	1	1	1	1	1	1	1	1	1	1	0	0
790	Kovarski_2021_Emotional visual mis	1	1	1	1	1	1	1	1	1	1	0	0
791	Astikainen_2013_Event-related potent	1	1	1	1	1	1	1	1	1	1	0	0
792	Stefanics_2012_Processing of unatte	1	1	1	1	1	1	1	1	1	1	0	0
793	Kreegipuu_2013_vMMN for schematic f	1	1	1	1	1	1	1	1	1	1	0	0
794	Csizmadia_2021_Older adults automat	1	1	1	1	1	1	1	1	1	1	0	0
795	Sayfulina_2020_Feature Binding in t	1	1	1	1	1	1	1	1	1	1	0	0
796	Athanasopoulos_2010_Perceptual shift in	1	1	1	1	1	1	1	1	1	1	0	0
797	Kovács-Bálint_2014_Automatic detection	1	1	1	1	1	1	1	1	1	1	0	0
798	Kecskés-Kovács_2013_Visual mismatch nega	1	1	1	1	1	1	1	1	1	1	0	0
799	Kecskés-Kovács_2013_Is it a face of a wo	1	1	1	1	1	1	1	1	1	1	0	0
800	Gaál_2017_When elderly outperf	1	1	1	1	1	1	1	1	1	1	0	0
801	Kimura_2010_Human visual system	1	1	1	1	1	1	1	1	1	1	0	0
802	Kimura_2018_Visual mismatch nega	1	1	1	1	1	1	1	1	1	1	0	0
803	Suzuki_2022_Proportion compatibi	1	1	1	1	1	1	1	1	1	1	0	0
804	Mao_2004_Event-related Potent	1	1	1	1	1	1	1	1	1	1	1	1
805	Liu_2008_Event-related potent	1	1	1	1	1	1	1	1	1	1	1	1
806	Wang_2014_Visual MMN elicited	1	1	1	1	1	1	1	1	1	1	1	1
807	Meng_2015_The automatic proces	1	1	1	1	1	1	1	1	1	1	1	1
808	Li_2016_Effect of mental fat	1	1	1	1	1	1	1	1	1	1	1	1
809	He_2014_Different effects of	1	1	1	1	1	1	1	1	1	1	1	1
810	Wang_2014_Automatic detection	1	1	1	1	1	1	1	1	1	1	1	1
811	Male_2020_The quest for the ge	1	1	1	1	1	1	1	1	1	1	1	1

812	Male_2023_Attention is require	1	1	1	1	1	1	1	1	1	1	1	1
813	Rowe_2020_Detecting (Un)seen C	1	1	1	1	1	1	1	1	1	1	1	1
814	M_2017_Short-term trained l	1	1	1	1	1	1	1	1	1	1	1	1
815	Yang_2020_Dysfunction of Pre-A	1	1	1	1	1	1	1	1	1	1	1	1
816	Male_2024_Orientation and cont	1	1	1	1	1	1	1	1	1	1	1	1
817	Cléry_2013_Electrophysiological	1	1	1	1	1	1	1	1	1	1	1	1
818	Smout_2019_Attention promotes t	1	1	1	1	1	1	1	1	1	1	1	1
819	Tugin_2016_Visual deviant stimu	1	1	1	1	1	1	1	1	1	1	1	1
820	Berti_2011_The attentional blin	1	1	1	1	1	1	1	1	1	1	1	1
821	Berti_2006_Visual distraction:	1	1	1	1	1	1	1	1	1	1	1	1
822	Durant_2017_Automatic detection	1	1	1	1	1	1	1	1	1	1	1	1
823	File_2020_Automatic change det	1	1	1	1	1	1	1	1	1	1	1	1
824	File_2019_Automatic detection	1	1	1	1	1	1	1	1	1	1	1	1
825	Froyen_2010_Exploring the role o	1	1	1	1	1	1	1	1	1	1	1	1
826	Tse_2021_Fronto-occipital mis	1	1	1	1	1	1	1	1	1	1	1	1
827	Durant_2018_Automatic detection	1	1	1	1	1	1	1	1	1	1	1	1
828	Dang_2023_Automatic sensory ch	1	1	1	1	1	1	1	1	1	1	1	1
829	Kaplan_2007_Late negativity in v	1	1	1	1	1	1	1	1	1	1	1	1
830	Suzuki_2024_The effects of posit	1	1	1	1	1	1	1	1	1	1	1	1
831	Zhang_2018_Perceptual expertise	1	1	1	1	1	1	1	1	1	1	1	1
832	Petro_2020_The effect of hand m	1	1	1	1	1	1	1	1	1	1	1	1
833	Urban_2008_Visual mismatch nega	1	1	1	1	1	1	1	1	1	1	1	1
834	Bottari_2014_Visual change detect	1	1	1	1	1	1	1	1	1	1	1	1
835	Maekawa_2011_Top-down and bottom-	1	1	1	1	1	1	1	1	1	1	1	1
836	Tales_2008_Visual mismatch nega	1	1	1	1	1	1	1	1	1	1	1	1
837	Flynn_2017_Visual mismatch nega	1	1	1	1	1	1	1	1	1	1	1	1
838	Schmitt_2021_Preattentive process	1	1	1	1	1	1	1	1	1	1	1	1
839	Kurita_2021_Involvement of visua	1	1	1	1	1	1	1	1	1	1	1	1
840	Langrová_2012_Gender Impact on Ele	1	1	1	1	1	1	1	1	1	1	1	1
841	Kimura_2019_Action-induced adjus	1	1	1	1	1	1	1	1	1	1	1	1
842	Hilger_2023_Intelligence and vis	1	1	1	1	1	1	1	1	1	1	1	1
843	Csikós_2023_Automatic detection	1	1	1	1	1	1	1	1	1	1	1	1
844	Molnár_2024_A comparison of visu	1	1	1	1	1	1	1	1	1	1	1	1
845	Kimura_2008_Attention switching	1	1	1	1	1	1	1	1	1	1	1	1
846	Kremlácek_2013_Visual mismatch nega	1	1	1	1	1	1	1	1	1	1	1	1
847	Czigler_2007_Backward masking and	1	1	1	1	1	1	1	1	1	1	1	1
848	Sulykos_2018_Automatic change det	1	1	1	1	1	1	1	1	1	1	1	1

849	Czigler_2019_Visual mismatch nega	1	1	1	1	1	1	1	1	1	1	1	1
850	Urakawa_2018_Enhancement of a gen	1	1	1	1	1	1	1	1	1	1	1	1
851	Sulykos_2015_Asymmetric effect of	1	1	1	1	1	1	1	1	1	1	1	1
852	Sulykos_2017_Visual mismatch nega	1	1	1	1	1	1	1	1	1	1	1	1
853	Intartaglia_2022_Visual cortex plasti	1	1	1	1	1	1	1	1	1	1	1	1
854	Henderson_2006_Is there a mismatch	1	1	1	1	1	1	1	1	1	1	1	1
855	Kojouharova_2019_Visual mismatch nega	1	1	1	1	1	1	1	1	1	1	1	1
856	Schlossmacher_2020_Differential effects	1	1	1	1	1	1	1	1	1	1	1	1
857	Schlossmacher_2022_Effects of awareness	1	1	1	1	1	1	1	1	1	1	1	1
858	Wu_2013_Human-computer inter	1	1	1	1	1	1	1	1	1	1	1	1
859	Li_2016_The magnitude of the	1	1	1	1	1	1	1	1	1	1	1	1
860	Y_1997_Mismatch negativity	1	1	1	1	1	1	1	1	1	1	1	1
861	Si_2014_Impairment in preatt	1	1	1	1	1	1	1	1	1	1	1	1
862	Mo_2011_Electrophysiological	1	1	1	1	1	1	1	1	1	1	1	1
863	Fu_2003_Event-related potent	1	1	1	1	1	1	1	1	1	1	1	1
864	Shi_2013_Visual mismatch nega	1	1	1	1	1	1	1	1	1	1	1	1
865	Nie_2022_Automatic detection	1	1	1	1	1	1	1	1	1	1	1	1
866	Nie_2020_Automatic detection	1	1	1	1	1	1	1	1	1	1	1	1
867	Qiu_2011_Impairment in proces	1	1	1	1	1	1	1	1	1	1	1	1
868	Yan_2017_Theta oscillations r	1	1	1	1	1	1	1	1	1	1	1	1
869	Mao_2005_Cognitive Impairment	1	1	1	1	1	1	1	1	1	1	1	1
870	Yang_2016_Gender differences i	1	1	1	1	1	1	1	1	1	1	1	1
871	Chen_2010_Differences in the n	1	1	1	1	1	1	1	1	1	1	1	1
872	Boll_2009_Distraction of task-	1	1	1	1	1	1	1	1	1	1	1	1
873	File_2017_Visual mismatch nega	1	1	1	1	1	1	1	1	1	1	1	1
874	Qian_2014_The visual mismatch	1	1	1	1	1	1	1	1	1	1	1	1
875	Nagai_2005_Mismatch negativity	1	1	1	1	1	1	1	1	1	1	1	1
876	Alho_1992_Intermodal selective	1	1	1	1	1	1	1	1	1	1	1	1
877	Hosak_2008_Mismatch negativity	1	1	1	1	1	1	1	1	1	1	1	1
878	Tales_1999_Mismatch negativity	1	1	1	1	1	1	1	1	1	1	1	1
879	Stagg_2004_Visual mismatch nega	1	1	1	1	1	1	1	1	1	1	1	1
880	Zhong_2015_Short−term trained l	1	1	1	1	1	1	1	1	1	1	1	1
881	Tomio_2012_Developmental change	1	1	1	1	1	1	1	1	1	1	1	1
882	Petro_2021_Automatic detection	1	1	1	1	1	1	1	1	1	1	1	1
883	Berti_2004_Distraction effects	1	1	1	1	1	1	1	1	1	1	1	1
884	Grimm_2009_Distraction in a vis	1	1	1	1	1	1	1	1	1	1	1	1
885	Hedge_2015_A frontal attention	1	1	1	1	1	1	1	1	1	1	1	1

886	Chang_2011_Dysfunction of preat	1	1	1	1	1	1	1	1	1	1	1	1
887	Tales_2006_Visual mismatch nega	1	1	1	1	1	1	1	1	1	1	1	1
888	Tales_2002_Age-related changes	1	1	1	1	1	1	1	1	1	1	1	1
889	Nyman_1990_Mismatch negativity	1	1	1	1	1	1	1	1	1	1	1	1
890	Noyce_2014_Oddball distractors	1	1	1	1	1	1	1	1	1	1	1	1
891	Müller_2012_Impact of lower-vs.	1	1	1	1	1	1	1	1	1	1	1	1
892	Jack_2017_Brain activity from	1	1	1	1	1	1	1	1	1	1	1	1
893	Kimura_2010_Top-down attention a	1	1	1	1	1	1	1	1	1	1	1	1
894	Kimura_2010_Human visual system	1	1	1	1	1	1	1	1	1	1	1	1
895	Wei_2002_A modified oddball p	1	1	1	1	1	1	1	1	1	1	1	1
896	Bodnár_2017_Automatic change det	1	1	1	1	1	1	1	1	1	1	1	1
897	Kimura_2015_Automatic prediction	1	1	1	1	1	1	1	1	1	1	1	1
898	Farkas_2015_Elementary sensory d	1	1	1	1	1	1	1	1	1	1	1	1
899	Kimura_2014_Voluntary action mod	1	1	1	1	1	1	1	1	1	1	1	1
900	Takács_2013_Oblique effect in vi	1	1	1	1	1	1	1	1	1	1	1	1
901	Kimura_2013_Task difficulty affe	1	1	1	1	1	1	1	1	1	1	1	1
902	Kimura_2009_Visual mismatch nega	1	1	1	1	1	1	1	1	1	1	1	1
903	Czigler_1990_Event–related potent	1	1	1	1	1	1	1	1	1	1	1	1
904	Czigler_2004_Visual change detect	1	1	1	1	1	1	1	1	1	1	1	1
905	Kimura_2006_Probability-independ	1	1	1	1	1	1	1	1	1	1	1	1
906	Czigler_2002_Memory-based detecti	1	1	1	1	1	1	1	1	1	1	1	1
907	Urakawa_2017_Involvement of the v	1	1	1	1	1	1	1	1	1	1	1	1
908	Maekawa_2009_Preattentive visual	1	1	1	1	1	1	1	1	1	1	1	1
909	Czigler_2006_Visual temporal wind	1	1	1	1	1	1	1	1	1	1	1	1
910	Amenedo_2007_Vertical asymmetries	1	1	1	1	1	1	1	1	1	1	1	1
911	Schmitt_2018_Preattentive and Pre	1	1	1	1	1	1	1	1	1	1	1	1
912	Woods_1992_Intermodal selective	1	1	1	1	1	1	1	1	1	1	1	1
913	Czigler_2010_Visual mismatch nega	1	1	1	1	1	1	1	1	1	1	1	1
914	Thierry_2009_Unconscious effects	1	1	1	1	1	1	1	1	1	1	1	1
915	Maekawa_2013_Altered visual infor	1	1	1	1	1	1	1	1	1	1	1	1
916	Sulykos_2011_One plus one is less	1	1	1	1	1	1	1	1	1	1	1	1
917	Maekawa_2005_Functional character	1	1	1	1	1	1	1	1	1	1	1	1
918	Sulykos_2014_Visual mismatch nega	1	1	1	1	1	1	1	1	1	1	1	1
919	Pesonen_2019_Long-term physical a	1	1	1	1	1	1	1	1	1	1	1	1
920	Sulykos_2013_Mismatch negativity	1	1	1	1	1	1	1	1	1	1	1	1
921	Czigler_2009_Unnoticed regularity	1	1	1	1	1	1	1	1	1	1	1	1
922	Stothart_2014_Double peaked P1 vis	1	1	1	1	1	1	1	1	1	1	1	1

923	Kuldkepp_2013_Unattended and atten	1	1	1	1	1	1	1	1	1	1	1	1
924	Clifford_2010_Color categories aff	1	1	1	1	1	1	1	1	1	1	1	1
925	Horimoto_2002_Mismatch negativity	1	1	1	1	1	1	1	1	1	1	1	1
926	Stothart_2013_Oscillatory characte	1	1	1	1	1	1	1	1	1	1	1	1
927	Cleary_2013_Investigating develo	1	1	1	1	1	1	1	1	1	1	1	1
928	Stothart_2013_Evoked potentials re	1	1	1	1	1	1	1	1	1	1	1	1
929	Kremláček_2006_Visual mismatch nega	1	1	1	1	1	1	1	1	1	1	1	1
930	Stefanics_2018_Visual mismatch and	1	1	1	1	1	1	1	1	1	1	1	1
931	Sysoeva_2015_From pre-attentive p	1	1	1	1	1	1	1	1	1	1	1	1
932	Stefanics_2011_Visual mismatch nega	1	1	1	1	1	1	1	1	1	1	1	1
933	Astikainen_2008_Visual mismatch nega	1	1	1	1	1	1	1	1	1	1	1	1
934	Kenemans_2010_Moderate alcohol dis	1	1	1	1	1	1	1	1	1	1	1	1
935	Kenemans_2003_Detection of visual	1	1	1	1	1	1	1	1	1	1	1	1
936	Astikainen_2004_The human brain proc	1	1	1	1	1	1	1	1	1	1	1	1
937	Bubrovszky_2011_Useful or not? How s	1	1	1	1	1	1	1	1	1	1	1	1
938	Maciejewska_2017_Visual Mismatch Nega	1	1	1	1	1	1	1	1	1	1	1	1
939	Pazo-Alvarez_2004_Effects of stimulus	1	1	1	1	1	1	1	1	1	1	1	1
940	Pazo-Alvarez_2004_Automatic detection	1	1	1	1	1	1	1	1	1	1	1	1
941	Berti_2009_Position but not col	1	1	1	1	1	1	1	1	1	1	1	1
942	Lorenzo-López_2004_Pre-attentive detect	1	1	1	1	1	1	1	1	1	1	1	1
943	Khodanovich_2010_A visual analog of m	1	1	1	1	1	1	1	1	1	1	1	1
944	Kimura_2018_Visual mismatch nega	1	1	1	1	1	1	1	1	1	1	1	1
945	O'Shea_2015_We make predictions	1	1	1	1	1	1	1	1	1	1	1	1
946	Berti_2018_Visual mismatch nega	1	1	1	1	1	1	1	1	1	1	1	1
947	Li_2017_The Pre-attentive Pr	1	1	1	1	1	1	1	1	1	1	1	1
948	Heslenfeld_2003_Visual mismatch nega	1	1	1	1	1	1	1	1	1	1	1	1
